# Prognostic prediction of dengue hemorrhagic fever in pediatric patients with suspected dengue infection: A multi-site study

**DOI:** 10.1371/journal.pone.0327360

**Published:** 2025-08-04

**Authors:** Myat Su Yin, Peter Haddawy, Panhavath Meth, Araya Srikaew, Chonnikarn Wavemanee, Saranath Lawpoolsri Niyom, Kanokwan Sriraksa, Wannee Limpitikul, Preedawadee Kittirat, Nasikarn Angkasekwinai, Oranich Navanukroh, Arunee Mapralub, Supansa Pakdee, Chotika Kaewpuak, Nattaya Tangthawornchaikul, Prida Malasit, Panisadee Avirutnan, Dumrong Mairiang

**Affiliations:** 1 Faculty of ICT, Mahidol University, Nakhon Pathom, Thailand; 2 Bremen Spatial Cognition Center, University of Bremen, Bremen, Germany; 3 Faculty of Tropical Medicine, Mahidol University, Bangkok, Thailand; 4 Pediatric Department, Khon Kaen Hospital, Khon Kaen, Thailand; 5 Pediatric Department, Songkhla Hospital, Songkhla, Thailand; 6 Department of Medicine, Faculty of Medicine, Siriraj Hospital, Mahidol University, Bangkok, Thailand; 7 Department of Preventive and Social Medicine, Faculty of Medicine Siriraj Hospital, Mahidol University, Bangkok, Thailand; 8 Siriraj Center of Research Excellence in Dengue and Emerging Pathogens, Faculty of Medicine Siriraj Hospital, Mahidol University, Bangkok, Thailand; 9 Medical Biotechnology Research Unit, National Center for Genetic Engineering and Biotechnology, National Science and Technology Development Agency, Pathum Thani, Thailand; 10 Division of Dengue Hemorrhagic Fever Research, Faculty of Medicine Siriraj Hospital, Mahidol University, Bangkok, Thailand; Universitas Padjadjaran, INDONESIA

## Abstract

Dengue virus (DENV) infection is a major global health problem. While DENV infection rarely results in serious complications, the more severe illness dengue hemorrhagic fever (DHF) has a significant mortality rate due to the associated plasma leakage that may lead to hypovolemic shock. Proper care thus requires identifying patients with DHF among those with suspected dengue so that they can be provided with adequate and prompt fluid replacement. In this study we used seventeen years of pediatric patient data from a prospective cohort study in two hospitals in Thailand to develop models to predict DHF among patients with suspected dengue infection. We produced models for a general hospital setting and for a primary care unit setting lacking lab facilities. The best model using combined data from both hospitals achieved an AUC of 0.90 for the general hospital setting and 0.79 for the primary care unit setting. We then investigated the generalizability of the models by training models with data from one hospital and testing them with data from the other. For some models, we found a significant reduction in performance. Possible sources of this are differences in how attributes are defined or measured and differences in the hematological parameters of the two patient populations. We conclude that while high accuracy prediction of DHF is possible, care must be taken when applying DHF predictive models from one clinical setting to another.

## Introduction

Dengue virus (DENV) infection is a major global health problem, affecting 390 million people annually [1]. Most symptomatic cases develop into dengue fever, which rarely results in serious complications, but less than 5% of patients will develop plasma leakage, signifying the more severe illness, dengue hemorrhagic fever (DHF) [2]. If DHF patients do not receive adequate and prompt fluid replacement, they may experience hypovolemic shock known as dengue shock syndrome (DSS), which has a significant fatality rate [3]. Given the large number of DENV infections in many countries, determining which patients have a high likelihood of DHF is an essential form of triage for hospital resource management.

Identification of DF and DHF patients from among the patients with suspected DENV infection is difficult in practice. Additionally, accurately diagnosing severe dengue (SD) or DHF requires expertise and specialized training. To help physicians diagnose DHF and SD in patients with suspected DENV infection, the World Health Organization released guidelines for DHF in 1997 and for SD in 2009 [3,4]. A systematic review by Horstick *et al*. showed that dengue classification using the 1997 WHO guidelines demonstrated a sensitivity of 24.8–89.9% and a specificity of 25–100% to accurately diagnose DHF cases [5]. The classification using the 2009 guidelines achieved a sensitivity of 59–98% and a specificity of 41–99% in diagnosing SD cases [5]. Horstick and his team also emphasized the importance of medical training and adapting the classification guidelines to local settings to ensure accurate identification of severe cases by physicians [5]. However, training physicians to treat dengue requires time and resources. In addition, the spread of DENV infections to new regions, where physicians lack experience in treating DENV-infected patients, is potentially exacerbated by climate change, which makes currently nonepidemic countries more favorable for mosquito vectors [6]. Prognostic prediction models could help identify severe cases using clinical and laboratory data collected from patients. The models could help healthcare personnel promptly and reliably identify SD or DHF cases to provide appropriate supportive care.

Several studies have developed statistical or machine learning models to predict severe cases in dengue patients. Phakhounthong *et al* [7] built a classification and regression tree to predict the development of severe dengue fever among pediatric patients with confirmed dengue infection. Their models have a moderate classification accuracy of 64.1%, a sensitivity of 60.5%, and a specificity of 65%. Lee and co-authors constructed a simple decision tree to identify potential adult DHF cases using only three variables: clinical hemorrhage history, serum urea, and total serum protein [8]. However, the accuracy was only 48.1%. Pongpan and team developed a scoring system that predictively stratified pediatric dengue patients into DF, DHF and DSS with an overall classification accuracy of 60.7 % [9]. In addition to these cross-sectional models, research has examined the use of longitudinal models. Since defervescence or plasma leakage usually occurs between 5 and 7 days after the onset of fever [3,4], patient data could be collected repeatedly in the form of a short longitudinal series from the first hospital visit to the critical day. A model or an algorithm that could utilize repeatedly measured features may have better performance compared to a cross-sectional model. Park and co-authors employed structural equation modeling that utilized short longitudinal data to develop predictive models for DF, DHF, and DSS for Thai pediatric patients with suspected DENV infection [10]. AUCs of the predictive model for DHF were in the range of 0.81 to 0.91 and 0.61 to 0.87 for study days 1 to 3 and illness days 2 to 5, respectively [10].

To accurately classify severe cases, laboratory tests, such as platelet count, white blood cell count, and liver function test, are often required [3,4]. However, DENV is primarily endemic in low- and middle-income countries where accessing laboratory tests may be difficult and expensive. When developing prognostic models for dengue patients, consideration should be given to settings with limited resources. Carrasco and colleagues developed generalized linear models using demographic, clinical, and laboratory variables of Singaporean adult dengue patients [11]. The models achieved specificities of 27% and 29% for a resource-limited setting (excluding laboratory tests) and a well-resourced setting, respectively [11].

In this study, we developed gradient boosting models for the two-class problem of predicting whether or not a patient will develop DHF, using a dataset of 17 years of short time series data on pediatric patients with suspected dengue from two hospitals in Thailand. In addition, we developed models for two clinical scenarios: a general hospital with full laboratory facilities and inpatient measurements and a primary care unit without laboratory facilities. The models achieve high accuracy, with an AUC ranging from 0.62 to 0.78 in primary care unit settings and from 0.78 to 0.90 in general hospital settings, thus outperforming previously published results on DHF prediction [7,8]. An important question for any ML application to clinical decision support is the extent to which the model performance holds across clinical sites. We evaluated the generalizability of this model by training on data from one hospital and testing on data from another. In some cases, this resulted in a significant decline in performance, which could be attributed to inconsistencies in attribute definitions or measurements, as well as variations in hematologic characteristics between the two populations of patients.

A key benefit of the models presented in this study is their ability to predict the prognosis of dengue (DHF) rather than diagnose the severity. The WHO 1997, 2009, and 2011 guidelines are only applicable during the critical phase, which usually occurs around the time of defervescence, when plasma leakage and severe organ involvement are already apparent. Conversely, our models are designed to provide prognostic information earlier, before or around the critical phase, when these clinical signs have not yet developed or are still difficult for clinicians to detect. This supports earlier identification of patients at risk.

This paper extends the preliminary results reported in a previous conference paper [12] in several ways. We perform a comprehensive statistical analysis of the data. We use a more robust model training approach and perform a more extensive model evaluation, including results for six prediction models and accuracy as a function of time to defervescence. Finally, we provide an extensive analysis of model generalizability, including factors that hamper this.

## Materials and methods

### Data

Data were obtained from a prospective cohort study conducted between 2001 and 2017, involving 1,845 pediatric patients under 15 years of age with suspected DENV infection. Patients enrolled in the cohort study were mostly already hospitalized as their signs or symptoms were deemed significant by doctors to potentially progress to DHF. The study was carried out at Khon Kaen Hospital (KK) in Khon Kaen province, northeastern Thailand, and Songkhla Hospital (SK) in Songkhla province, southern Thailand. Both hospitals are tertiary referral and teaching centers staffed by trained pediatricians. KK, a 1,000-bed regional hospital, is located in a tropical savanna climate with a rainy season that lasts from May to October. SK, a 500-bed general hospital, is in a tropical monsoon climate with a rainy season from July to January [13–15].

Patients under 15 years of age with an axillary temperature greater than 38 °C, fever lasting up to 4 days, signs of dengue infection, and no localized infection were included. Standardized data collection protocols were used at both hospitals, with slight variability in measurements possible. Data were managed using REDCap tools [16] hosted by the Research Data Management Unit, Faculty of Medicine, Siriraj Hospital, Mahidol University. Data collection focused on the period prior to defervescence (days of fever 3–7) [2], targeting patients in the early stages of infection. Daily chest radiographs or ultrasounds were performed to detect pleural effusion, a marker of plasma leakage in DHF.

DENV infection was confirmed using nested RT-PCR [17] and IgM/IgG capture ELISA [18], following the 1997 WHO dengue guidelines [19]. ELISA analyzed paired samples from the acute and convalescent phases (approximately 14 days apart). PCR and ELISA provided information on dengue serotypes and distinguished between primary and secondary infections (see S8 File). Diagnostic criteria for DHF included increases in hematocrit greater than 20%, ascites, and hypoalbuminemia. To focus on early-stage detection, data collected after the day of defervescence were excluded because plasma leakage, the outcome of interest, had already occurred by that point. The severity of the disease was independently classified by three physicians based on clinical, laboratory, and imaging findings. The final classifications into dengue (DF), dengue hemorrhagic fever (DHF), or other febrile illnesses(OFI) required the agreement by at least two physicians.

The final combined multisite data set included 5,037 study days from 1,733 patients, distributed as follows: 150 OFI cases, 766 DF cases, and 817 DHF cases. At the individual site level, Songkhla Hospital (SK) reported 68 OFI cases, 423 DF cases, and 403 DHF cases, while Khon Kaen Hospital (KK) reported 82 OFI cases, 343 DF cases, and 414 DHF cases. Since the primary objective of this study was to identify DHF patients among those with suspected DENV infection, OFI and DF patients were combined into a Non-DHF group. Data for this study were obtained from hospitals that had screened patients for suspected dengue using their standard procedures, so the inclusion of OFI patients reflects the normal imprecision of that screening process.

The features in the dataset can be grouped into four categories: demographics and baseline physical examination/interview (9 features), daily vital signs (17 features), daily physical examination/interview (20 features), and daily laboratory check-up measures (17) Table 1. The demographic and baseline characteristics of the physical examination were obtained once upon admission. Physical examination/interview and laboratory checkup were obtained daily. Vital signs were measured multiple times per day. These features were supplemented with calculated ratios, ranges, and average values for quantitative features.

**Table 1:** Data dictionary.

Feature name	Type	Frequency of measurement
Demographics and baseline physical examination/interview		
Day of fever	Categorical	Derived
Bleeding (On Admission)	Binary	Once on admission
Height	Numeric	Once on admission
Japanese Encephalitis Vaccine Status	Binary	Once on admission
Quality of tourniquet (On Admission)	Numeric	Once on admission
Quantity of tourniquet (On Admission)	Categorical	Once on admission
Sex	Binary	Once on admission
Upper Respiratory Tract Infection (URI, On Admission)	Binary	Once on admission
Daily physical examination/interview		
Weight	Numeric	Once daily
Abdominal circumference	Numeric	Once daily
Abdominal pain	Binary	Once daily
Bleeding (Daliy examination)	Binary	Once daily
Bruising	Binary	Once daily
Rash	Binary	Once daily
Diarrhea	Binary	Once daily
Itching	Binary	Once daily
Limbal conjunctival injection	Binary	Once daily
Liver tenderness	Binary	Once daily
Liver size	Numeric	Once daily
Lymph node	Binary	Once daily
Maculopapular rash	Binary	Once daily
Mental status	Categorical	Once daily
Non-productive cough	Binary	Once daily
Quality of tourniquet (Daliy examination)	Numeric	Once daily
Quantity of tourniquet (Daliy examination)	Categorical	Once daily
Presence of rash	Binary	Once daily
Spontaneous petechiae	Binary	Once daily
URI (Daily examination)	Binary	Once daily
Diastolic blood pressure	Numeric	Multiple times daily
Average fingerstick hematocrit	Numeric	Multiple times daily
Maximum fingerstick hematocrit	Numeric	Multiple times daily
Minimum fingerstick hematocrit	Numeric	Multiple times daily
Fingerstick hematocrit range	Numeric	Multiple times daily
Fluid intake and output differences	Numeric	Multiple times daily
Daily maximum fluid intake and output differences	Numeric	Multiple times daily
Minimum pulse pressure	Numeric	Multiple times daily
Average pulse rate	Numeric	Multiple times daily
Maximum pulse rate	Numeric	Multiple times daily
Minimum pulse rate	Numeric	Multiple times daily
Pulse rate range	Numeric	Multiple times daily
Systolic blood pressure	Numeric	Multiple times daily
Average daily body temperature	Numeric	Multiple times daily
Maximum daily body temperature	Numeric	Multiple times daily
Minimum daily body temperature	Numeric	Multiple times daily
Body temperature range	Numeric	Multiple times daily
Daily laboratory checkup measures		
Albumin to Globulin ratio	Numeric	Derived
Atypical lymphocytes	Numeric	Once daily
Band neutrophils	Numeric	Once daily
Basophils	Numeric	Once daily
Eosinophils	Numeric	Once daily
HCT (laboratory)	Numeric	Once daily
Albumin	Numeric	Once daily
Alanine Aminotransferase (ALT)	Numeric	Once daily
Aspartate Aminotransferase (AST)	Numeric	Once daily
Protein	Numeric	Once daily
Lymphocyte	Numeric	Once daily
Monocytes count	Numeric	Once daily
Platelet count	Numeric	Once daily
Polymorphonuclear Leukocytes (PMN)	Numeric	Once daily
AST/platelet ratio	Numeric	Derived
AST/ALT ratio	Numeric	Derived
White Blood Count (WBC)	Numeric	Once daily

In dengue-endemic countries, primary care units (PCUs) are often the first point of care for most patients. It is thus important to build and assess prognostic models for these settings. In Thailand’s PCUs, laboratory tests, imaging, and molecular diagnostics are unavailable at the point of care. We thus simulated the PCU setting by creating datasets in which these features were removed. Thai clinicians were consulted to ensure that the selected features were realistic and could be collected in such settings.

### Exploratory data analysis

There were 909 males and 824 females, and the median age of the patients was ten years. The numerical variables of symptoms and biological parameters are summarized using medians and interquartile ranges (IQR) in Table 2. In general, patients were admitted the third day after fever onset (median = 3, IQR = (2,4)), for a median stay of 3 days (IQR = (2,4)). Next, to determine the odds ratio of the variables, a univariate logistic regression analysis was performed, treating each potential factor as an independent variable and the final DHF diagnosis status as the dependent variable. The confidence intervals for the odds ratios were calculated for each predictor. The odds ratios for numeric variables with 95% confidence interval are shown in Table 2. This analysis allowed for the evaluation of the association between each potential factor and the severity of dengue infection. All statistical tests in exploratory data analysis were two-sided, with a significance level of p ≤ 0.05 considered statistically significant, indicating that the observed results were unlikely to have occurred by chance alone. Statistical analyses were performed with R programming language (version 4.1.2). The analysis scripts are available at https://github.com/si-medbif/dengue_dx_ai.

**Table 2:** Numeric variables from symptoms and biological parameters with medians (inter-quartile ranges) and odds ratios for DHF and Non-DHF categories.

Description	Unit	Non-DHF	DHF	Odds Ratio
Age	year	10 (7, 12)	11 (8, 13)	1.10 (1.08, 1.13)
Height	cm	135 (119, 147)	140 (124, 153)	1.02 (1.01, 1.02)
Daily blood pressure				
Systolic	mmHg	98 (90, 100)	98 (90, 100)	1.00 (1.00, 1.01)
Diastolic	mmHg	60 (55, 60)	60 (56, 60)	1.02 (1.01, 1.02)
Daily pulse pressure				
Minimum	mmHg	31 (30, 40)		
Daily fingertip hematocrit				
Minimum	%	37 (35, 39)	38 (35, 40)	1.10 (1.08, 1.12)
Maximum	%	39 (36, 41)	41 (38, 44)	1.18 (1.16, 1.20)
Average	%	38 (36, 40)	40 (37, 42)	1.16 (1.14, 1.18)
Range	%	2 (0, 3)	3 (1, 5)	1.28 (1.24, 1.31)
Fluid intake and output				
Difference between fluid intake and output	ml	250 (0, 700)	500 (0, 1100)	1.00 (1.00, 1.00)
Daily maximum difference between fluid intake and output	ml	270 (0, 485)	370 (0, 630)	1.00 (1.00, 1.00)
Daily pulse rate				
Minimum	beats/minute	88 (80, 96)	86 (80, 96)	0.99 (0.99, 1.00)
Maximum	beats/minute	108 (98, 116)	108 (98, 116)	1.00 (1.00, 1.01)
Average	beats/minute	97 (90, 105)	97 (89, 105)	1.00 (0.99, 1.00)
Range	beats/minute	18 (12, 26)	20 (12, 28)	1.01 (1.01, 1.02)
Daily body temperature				
Minimum	°C	36.9 (36.5, 37.5)	37.0 (36.5, 37.8)	1.27 (1.19, 1.36)
Maximum	°C	38.9 (38.2, 39.5)	39.2 (38.5, 39.8)	1.25 (1.18, 1.33)
Average	°C	37.9 (37.4, 38.4)	38.1 (37.5, 38.7)	1.37 (1.27, 1.47)
Range	°C	1.8 (1.2, 2.4)	1.9 (1.2, 2.5)	1.04 (0.98, 1.11)
Abdominal circumference	cm	55.0 (50.0, 63.0)	58.5 (52.0, 64.0)	1.02 (1.01, 1.02)
Liver size	cm	0.0 (0.0, 1.0)	0.5 (0.0, 2.0)	1.40 (1.33, 1.48)
body weight	kg	27.2 (20.8, 39.6)	31.6 (22.9, 43.0)	1.01 (1.01, 1.02)
Venipuncture hematocrit	%	36.2 (34.0, 38.9)	38.0 (35.5, 40.9)	1.13 (1.11, 1.14)
Atypical lymphocyte count*	cells/mm^3^	52 (0, 158)	59 (0, 163)	37.21 ( 9.45, 146.95)
Band cell count*	cells/mm^3^	0 (0, 165)	0 (0, 128)	101.29 ( 13.34, 774.87)
Basophil count*	cells/mm^3^	0 (0, 96)	0 (0, 100)	0.24 (0.01, 4.05)
Eosinophil count*	cells/mm^3^	0 (0, 131)	0 (0.00, 128)	0.00 (0.00, 0.01)
Lymphocyte count*	cells/mm^3^	108 (0, 168)	104 (0, 195)	0.00 (0.00, 0.00)
Monocyte count*	cells/mm^3^	80 (0, 163)	76 (0, 153)	0.00 (0.00, 0.00)
Polymorphonuclear Leukocyte count*	cells/mm^3^	108 056, 190)	108 (0, 187)	0.00 (0.00, 0.12)
White blood cell count*	x1000 cells/mm^3^	3.5 (3.4, 3.6)	3.5 (3.3, 3.6)	0.39 (0.30, 0.51)
Albumin	g/dL	3.5 (3.3, 3.8)	3.3 (3.0, 3.6)	0.30 (0.26, 0.34)
ALT	IU/L	37 (26, 57)	51 (36, 83)	1.01 (1.01, 1.01)
AST	IU/L	63 (42, 99)	103 (67, 181)	1.00 (1.00, 1.01)
Total protein	g/dL	6.7 (6.3, 7)	6.4 (5.9, 6.8)	0.50 (0.46, 0.54)
Platelet count	x1000 cells/mm^3^	130 (91, 179)	76 (42, 119)	0.99 (0.98, 0.99)
Albumin:Globulin ratio	-	0.53 (0.50, 0.56)	0.53 (0.49, 0.56)	0.12 (0.04, 0.33)
AST:Platelet ratio	(IU/L)/( x1000 cells/mm^3^)	0.49 (0.25, 1.08)	1.56 (0.65, 3.95)	1.38 (1.33, 1.42)
AST:ALT ratio	-	1.70 (1.25, 2.35)	2.00 (1.54, 2.65)	1.32 (1.24, 1.40)
Day of illness	day	4 (3, 5)	4 (3, 5)	0.91 (0.88, 0.95)

Note: AST: Aspartate Transaminase, ALT: Alanine Transaminase.

*Data are presented as median (min, max).

**Fig 1:**
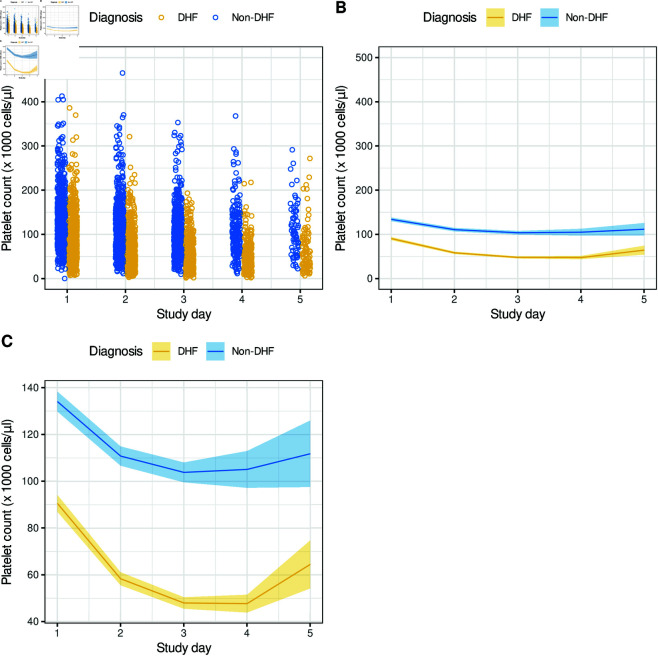
Time course of “Platelet count” variable from study day one to five in two groups (DHF and Non-DHF). The data are shown as raw values (A) and means by group by study day (B) with the shaded areas representing 95% confident intervals of the means. The mean values (B) are also zoomed in to show the differences and trajectories of the two groups (C).

**Fig 2:**
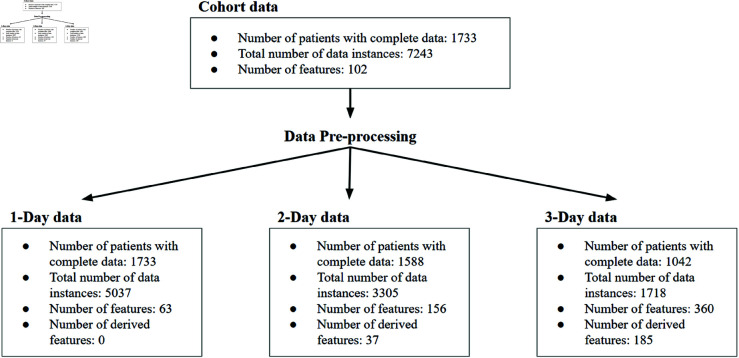
Overview of the dataset.

Exploratory data analysis revealed that some quantitative features, such as Platelet count, differ between DHF and Non-DHF patients not only in their values at specific points in time but also in the patterns of their trajectories over time. Fig 1 shows the time course of the Platelet count variable from study day one to day five. Time courses for other variables are provided in S1 and S2 Files (study day alignment) and S9 and S10 Files (fever day alignment). In both alignments, the trajectories are distinct for the two categories. For each quantitative feature, we thus added estimates of the slope, using the difference between two study day values and the second derivative based on three study day values. Examples of such derivatives include the differences between maximum temperature between study days 1 and 2, and the acceleration or deceleration of differences in platelet counts from study days 1, 2 and 3. We also added the slope and the Y-intercept of the least-squares fit to three study-day values. After generating these additional features, there were 76, 179 and 391 characteristics per patient for study days 1, 2, and 3, respectively (Fig 2). Qualitative features (S2 File) were represented as binary, i.e., 0 and 1.

### Models and settings

First, we discretized continuous variables from symptoms and biological parameters using entropy-based discretization [20], which selects splits that minimize the entropy of the partitions relative to the target class. Our experiments found this to improve the accuracy of the model. This technique can be helpful when attribute values have very long tails. Examples of the resulting intervals after discretization for four variables (Abdominal circumference, Weight, Maximum daily body temperature, Platelet count variables) are shown in Table 3. The number of bins for discretization in this study was determined on the basis of experimental results, with the chosen value set at nine bins. The odd ratios of the discretized variables are provided in S3-1 Table.

**Table 3:** Example of interval of features after discretization.

Abdominal circumference	body weight	Max- daily body temperature	Platelet count
(-inf, 44.75]	(-inf, 12.55]	(-inf, 37.15]	(-inf, 8.5]
(44.75, 46.25]	(12.55, 13.15]	(37.15, 38.35]	(8.5, 21.5]
(46.25, 47.625]	(13.15, 13.95]	(38.35, 38.95]	(21.5, 40.5]
(47.625, 48.25]	(13.95, 18.05]	(38.95, 39.05]	(40.5, 44.5]
(48.25, 58.008]	(18.05, 28.35]	(39.05, 39.65]	(44.5, 69.5]
(58.008, 59.091]	(28.35, 28.55]	(39.65, 40.65]	(69.5, 104.25]
(59.091, 59.865]	(28.55, 28.75]	(40.65, 40.85]	(104.25, 129.75]
(59.865, 63.25]	(28.75, 34.95]	(40.85, 40.95]	(129.75, 180.75]
(63.25, inf]	(34.95, inf]	(40.95, inf]	(180.75, inf]

Predicting the progression of DHF involves distinguishing between different patient outcomes, which amounts to a classification problem. The data contains labeled outcomes (OFI, DF and DHF), making classification an appropriate choice for modeling the progression. We constructed models using various algorithms, including Random Forest, Light Gradient Boosting Machine (LightGBM) [21], and Support Vector Machine. Among these, LightGBM demonstrated superior performance, leading to its selection for the classification task. LightGBM is a gradient-boosting framework optimized for decision tree-based models. It employs a gradient-based approach to iteratively refine predictions, focusing on key performance areas.

Patient features (physical examination, interview, lab values) are available for each day from the initial dengue diagnosis until the patient recovers or develops DHF. Thus, there is an increasing amount of information about the patient over time that predictive modeling should take advantage of. Thus, we developed a different model for each study day, taking into account the information that would be available on that day. The Day 1 model (1-Day) used only the admission-day data of each patient. Additional data from each study day was added for the models on day 2 (2-day) and on day 3 (3-day). The number of patients on study days 4 and 5 was insufficient to train the models, so we did not create models for those study days. We developed prognostic models for two clinical settings: resource-limited primary care units (PCU) with only clinical data and well-resourced general hospitals (GH) with clinical, laboratory and inpatient ward data.

Feature selection is a critical step in model development, as too many features can lead to overfitting and make models impractical, particularly when patient data must be manually entered. To address this, we calculated feature importance scores that quantify each feature’s contribution to the model. Nested cross-validation was employed to ensure unbiased feature importance estimates while avoiding overfitting during hyperparameter tuning. An outer cross-validation loop simulated independent training and testing, while inner cross-validation optimized hyperparameters and selected the best model. Feature importance was calculated using the LightGBM gain method[22], which measures how much each feature improves the accuracy of the model when the decision tree nodes are split. Gain values were averaged across the outer folds to identify features that consistently improved model performance. The top 30 features were selected as a candidate set based on their importance scores and a greedy forward selection approach was applied to determine the optimal subset of features. Features were incrementally added in importance order until no further improvement in model performance was observed. This process retained only the most critical features. The study physicians then reviewed the final set of selected characteristics to assess their clinical relevance in patients with dengue.

**Fig 3:**
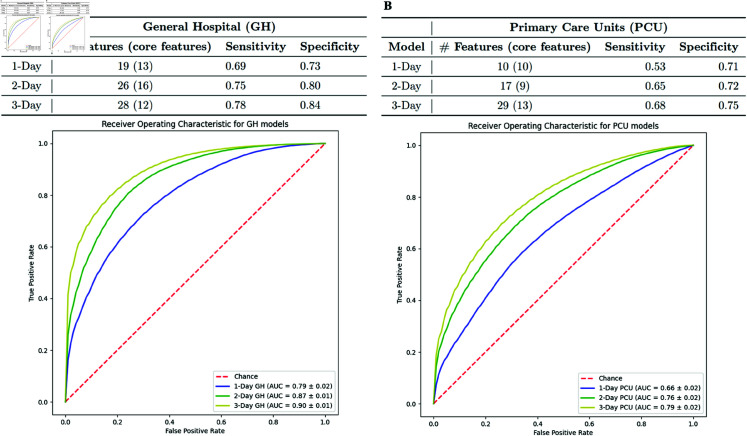
Performance results of the models trained with combined multi-site data A. General Hospital (GH) scenario B. Primary care unit (PCU) scenario.

The selected features included both core and derived features, with derived features dynamically computed from the core features. Core features are commonly collected variables in PCU and GH clinical settings. For example, in the 1-Day (PCU) model shown in Fig 3, ‘19(13)’ indicates that 19 features were included, 13 of which are core features and 6 derived. In practice, collecting the 13 core features would be sufficient to make predictions on the day of admission.

The final models included 10 to 29 features (9-16 core) for the PCU setting and 19 to 28 features (12-16 core) for the GH setting, as shown in Fig 3. Detailed lists of core features for models 1, 2, and 3 in both settings are provided in S6 File.

To evaluate model performance for the combined multisite dataset, we applied stratified bootstrapping with a 70/30 training-testing split, repeated 1,000 times to ensure robust results. The area under the receiver operating characteristic curve (AUC) was the primary evaluation metric, as it reflects the model’s ability to distinguish between classes across thresholds. Values above 0.7 are considered acceptable, while values exceeding 0.8 or 0.9 indicate strong performance. AUC correlates indirectly with the accuracy, as higher AUC values correspond to better accuracy at optimal thresholds. Sensitivity and specificity were reported alongside the AUC to provide a more detailed understanding of the performance of the model. Sensitivity is crucial in medical diagnostics to minimize missed cases, reducing the risk of delayed treatment or disease progression. Specificity focuses on reducing false positives, which is typically less critical in such applications.

### Generalizability study

Model accuracy may differ for clinical settings or geographic regions beyond those from which the original data came [23]. It is of great importance to understand such variability if we wish to be able to create models that can be widely disseminated and effectively used in diverse settings. We explored this in our context of DHF prediction by building a model using data from each of our two hospitals and then evaluating the model using the data from the other hospital to simulate a deployment of the models in a new geographic region. Since the data was collected in a prospective study, with the same data collection protocol followed in both hospitals, any variability would be due only to how features were defined and measured and to variations in patient populations.

### Ethics statement

The study was approved by the Institutional Review Board (IRB) and the Ethics Committee for Human Subjects at the Faculty of Medicine, Siriraj Hospital, Mahidol University, Bangkok, Thailand (approval number 167/2565(IRB3)). The requirement for informed consent was waived. The study retrieved the fully anonymized data from the pediatric dengue clinical cohort study. The cohort study was approved by the Institutional Review Board (IRB) and the Ethics Committee for Human Subjects at the Faculty of Medicine, Siriraj Hospital, Mahidol University, Bangkok, Thailand (approval number 156/2002, 349/2007 and 632/2016); the Ethical Review Committee for Research in Human Subjects of the Ministry of Public Health (ECMOPH), Nonthaburi, Thailand (approval number 92/2007); the Ethical Review Committee for Research in Human Subjects of Khon Kaen Hospital, Khon Kaen, Thailand (approval number KE60108); and the Ethical Review Committee for Research in Human Subjects of Songkhla Hospital, Songkhla, Thailand (approval number 11/256). Written informed consents were obtained from the parents of the cohort study participants. Furthermore, participants who were seven years of age or older gave written assents to the cohort study. Participants and parents were also informed that the anonymized data and specimens would be utilized in future studies with the approval of the appropriate ethical committees.

## Results

### LightGBM models

The dataset includes a small number of OFI patients whose symptoms often resemble those of DF, making it important to assess their impact on classification tasks. To address this, we performed a sensitivity analysis to evaluate the effects of including and excluding OFI patients. Excluding OFI patients from the combined multisite dataset resulted in a classification task of DF versus DHF (766 DF vs. 817 DHF). Including 150 OFI patients alongside 766 DF instances created a Non-DHF category (916 Non-DHF vs. 817 DHF), shifting the focus to distinguishing Non-DHF from DHF.

Table 4 presents the AUCs for models with and without OFI cases. In the Non-DHF vs. DHF setting, models show a consistent increase in AUC from the 1-Day to 3-Day models, with GH consistently outperforming PCU. A similar trend is observed in the DF vs. DHF setting when OFI cases are excluded. For the remainder of this paper, we focus on the Non-DHF versus DHF classification task.

**Table 4:** AUCs of models with OFI cases (Non-DHF vs. DHF classification) and without OFI cases (DF vs. DHF classification) trained and tested on the combined multi-site dataset.

Model	Scenario	Non-DHF vs DHF	DF vs DHF
1-Day	GH	0.79 (0.76, 0.82)	0.76 (0.73, 0.80)
	PCU	0.66 (0.62, 0.70)	0.65 (0.61, 0.69)
2-Day	GH	0.87 (0.84, 0.89)	0.85 (0.82, 0.88)
	PCU	0.76 (0.72, 0.79)	0.74 (0.70, 0.79)
3-Day	GH	0.90 (0.87, 0.93)	0.88 (0.85, 0.91)
	PCU	0.79 (0.75, 0.83)	0.78 (0.73, 0.82)

Table 5 presents the features and their importance scores for the 1-Day GH and 1-Day PCU settings from the combined multisite data set. The 2-Day and 3-Day models include 21% to 41% derived features, primarily based on slope and second derivative calculations (see Tables 6 and 7). Detailed lists of selected features categorized into five groups (four from the co-hort study and one additional group for derived features), are provided in S4 File. These lists cover models from both GH and PCU settings across combined multisite and individual-site datasets. Table 8 shows the union of the top five features of the study day 1, 2, and 3 models for the multi-site dataset for both the PCU and the GH settings. The selected features align with clinically significant markers for monitoring the progression and severity of DHF.

**Table 5:** Features with importance scores from General Hospital (GH) and Primary Care Unit (PCU) scenarios from study Day 1 (1-Day) model.

	1-Day General Hospital (GH)		1-Day Primary Care Unit (PCU)	
	Features	Score	Features	Score
1	AST/platelet ratio	3564.43	Abdominal Circumference*	1972.24
2	Platelet count*	2793.55	Liver size*	1855.83
3	Day of fever	1549.00	Average daily body temperature	1608.69
4	Maximum fingerstick hematocrit*	1547.54	Age	1561.18
5	Lymphocyte	1346.88	Abdominal pain*	1199.53
6	Abdominal Circumference*	1327.5	Quantity of tourniquet (on admission)	906.72
7	Average daily body temperature	1270.54	Day of fever	885.75
8	Fingerstick hematocrit range*	1230.53	Sex	761.07
9	Albumin	1153.95	body weight	752.89
10	Daily max fluid intake and output differences*	843.57	Minimum daily pulse pressure	725.83
11	AST	784.43		
12	Maximum daily body temperature	708.95		
13	Protein	660.03		
14	Age	644.94		
15	Minimum daily body temperature	590.09		
16	Fluid intake and output differences*	574.50		
17	ALT	572.65		
18	AST/ALT ratio	568.35		
19	Liver size*	549.34		

Note: Aspartate Transaminase (AST), and Alanine Transaminase (ALT) are liver enzymes

*Variables are in accordance with WHO Dengue fever diagnosis guidelines

**Table 6:** Features with importance scores from General Hospital (GH) and Primary Care Unit (PCU) scenarios from study Day 2 model.

	2-Day General Hospital(GH)		2-Day Primary Care Unit (PCU)	
	Features	Score	Features	Score
1	Platelet count* (Day2)	3185.02	Liver size (Day2)*	1220.81
2	AST/platelet ratio (Day2)*	1766.62	Average daily body temperature (Day2)	1199.61
3	Average daily body temperature (Day2)*	1461.14	Abdominal pain (Day2)*	1151.84
4	Platelet count (diff. between Day 2-1)*	990.94	Abdominal Circumference (diff. between Day 1-2)*	1075.6
5	Maximum fingerstick hematocrit (Day2)*	931.21	Age	1071.27
6	Fingerstick hematocrit range (Day2)*	924.52	Average daily body temperature (Day1)	865.81
7	Day of fever	778.57	Abdominal Circumference (Day1)*	812.12
8	Albumin (Day2)	734.44	Abdominal Circumference(Day2)*	735.95
9	Lymphocyte (Day2)	698.56	body weight (diff. between Day 1-2)	705.28
10	Liver size (Day2)*	659.7	Day of fever	650.15
11	Fluid intake and output differences (Day2)*	646.6	Liver size (diff. between Day 1-2)*	635.81
12	Fingerstick hematocrit range (Day1)*	514.26	Minimum daily pulse pressure (diff. between Day 1-2)	615.78
13	AST/ALT ratio (Day1)	493.58	body weight (Day1)	595.51
14	PMN (Day1)	452.28	Average daily body temperature (diff. between Day 1-2)	580.9
15	Average fingerstick hematocrit (diff. between Day 1-2)*	448.1	Average daily pulse pressure (Day2)	504.41
16	AST/platelet ratio (diff. between Day 1-2)*	446.54	Quantity of tourniquet (Daily examination) (Day2)	402.2
17	Maximum daily body temperature (Day2)	434.42	Sex	390.55
18	ALT (Day1)	423.71		
19	Age	415.11		
20	Protein_(Day2)	402.57		
21	Maximum fingerstick hematocrit range (diff. between Day 1-2)*	395.55		
22	Lymphocyte (Day1)	340.89		
23	Minimum daily body temperature (Day2)	304.56		
24	Sex	278		
25	HCT (laboratory) (Day2)*	266.72		
26	Abdominal pain (Day2)*	242.11		

Note: Aspartate Transaminase (AST), and Alanine Transaminase (ALT) are liver enzymes

*Variables are in accordance with WHO Dengue fever diagnosis guidelines

**Table 7:** Features with importance scores from General Hospital (GH) and Primary Care Unit (PCU) scenarios from Study Day 3 model.

	3-Day General Hospital (GH)		3-Day Primary Care Unit(PCU)	
	Features	Score	Features	Score
1	Platelet count (Day3)*	1899.3	Abdominal pain (Day3)*	870.65
2	AST/platelet ratio (Slope)*	1122.18	Average daily body temperature (Day2)	613.23
3	Platelet count (Slope)*	816.86	Liver size_Day3*	612.53
5	Fingerstick hematocrit range (Day3)*	406.62	Age	478.3
6	Fluid intake and output differences_Day3*	396.19	body weight (Slope)	456.05
7	Liver size (Day3)*	381.02	Liver size (Slope)*	447.54
8	Maximum fingerstick hematocrit (Slope)*	377.76	Minimum daily pulse pressure (Day3)	369.47
9	WBC (Slope)	370.05	Average daily body temperature (Intercept)	358.4
10	Daily max fluid intake and output differences (Day3)*	366.18	Abdominal Circumference (Day1)*	347.39
11	Maximum daily body temperature_Day3	330.81	Day of fever	338.96
12	Average daily body temperature (Day3)	328.15	Minimum daily blood pressure (Systolic) (Slope)	338.34
13	Albumin (Day3)	323.7	Abdominal Circumference (diff. between Day 1-3)*	322.5
14	Daily max fluid intake and output differences (diff. between Day 2-3)*	295.52	Average daily body temperature (Day3)	318.32
15	Average daily body temperature (Day2)	271.75	body weight (diff. between Day 2-3)	306.93
16	Average daily body temperature (Day1)	264.15	Average daily body temperature (Day1)	297.25
17	Average fingerstick hematocrit (Slope)*	258.06	Minimum daily pulse pressure (Day2)	284.07
18	Abdominal Circumference (Slope)*	252.33	Abdominal Circumference (Intercept)*	278.02
19	HCT (laboratory) (Slope)*	244.11	Average daily body temperature (diff. between Day 2-3)	264.48
20	AST/ALT ratio (Slope)	243.51	Quantity of tourniquet (Daily examination) (Day1)	255.86
21	Albumin (diff. between Day 2-3)	227.67	body weight (diff. between Day 1-3)	250.42
22	Platelet count (Day2)*	219.44	Quantity of tourniquet (on admission)	244.26
23	Platelet count (diff. between Day 2-3)*	210.44	Minimum daily pulse pressure (Intercept)	237.15
24	Protein (Day3)	207.31	Abdominal Circumference (Day2)*	212.25
25	Minimum fingerstick hematocrit (Day3)*	201.26	Quantity of tourniquet (Daily examination) (Day2)	208.93
26	Albumin (Slope)	184.07	Abdominal Circumference (diff. between Day 1-2)*	208.9
27	Minimum daily body temperature (Day3)	169.82	Average daily body temperature (Slope)	191.93
28	Protein (Slope)	121	Injected conjunctivae (Day2)	172.38
29			Quality of tourniquet (Daily examination) (Day1)	110.65

Note: Aspartate Transaminase (AST), and Alanine Transaminase (ALT) are liver enzymes

*Variables are in accordance with WHO Dengue fever diagnosis guidelines

**Table 8:** Top five features obtained from the union of the feature set of study day 1, 2, and 3 models.

General Hospital (GH)	Primary Care Unit (PCU)
AST/Platelet Ratio	Abdominal Circumference
Platelet count	Liver Size
Maximum fingerstick hematocrit	Average daily body temperature
Average daily body temperature	Age
Fingerstick hematocrit range	Abdominal Pain
Albumin	Weight

For the PCU setting where laboratory variables are not available, the features in Table 8 consist primarily of the findings of the physical examination and the observable symptoms. Abdominal circumference and daily weight act as surrogate indicators of fluid retention, a key factor in severe dengue caused by plasma leakage. These measures are valuable when direct assessments, such as intake and output monitoring, are not feasible. Liver size, associated with hepatomegaly, reflects disease severity and its impact on organ function. Abdominal pain and liver tenderness are common in DHF cases. Age is another important feature, as older patients are more prone to secondary infections related to increased disease severity. Average body temperature, elevated in DHF cases, particularly near the day of defervescence, highlights the systemic immune response during critical stages.

In the GH setting, the selected features include laboratory-based variables. The AST/platelet ratio, often elevated in DHF, reflects liver stress and changes in blood composition. Platelet count, typically reduced in DHF, highlights thrombocytopenia and increased vascular permeability. Hematocrit measures, such as maximum daily fingerstick hematocrit and HCT range, indicate plasma leakage, with increases in hematocrit that exceed 20% from baseline serving as a marker of severe cases. Average body temperature remains a key feature, reflecting the elevated fever associated with DHF near defervescence. Albumin, often reduced in DHF, reflects plasma leakage and capillary permeability, helping to assess fluid loss in GH settings.

### Model performance

To classify Non-DHF versus DHF, we trained models on the multisite dataset and evaluated their performance using the multi-site test set and using the test sets from the individual hospitals.

Table 9 presents the performance of models trained on the multi-site dataset, evaluated using AUC (with 95% confidence intervals) over the multi-site test set, SK-Hospital test set, and KK-Hospital test set. GH models consistently have higher AUC values than the PCU models for each day. This is not surprising since the GH models have lab values available. Performance improves in both settings as the number of study days increases, with the largest improvements occurring between the 1-Day and 2-Day models. For example, the GH model improves from an AUC of 0.79 on the 1-Day multisite test set to 0.90 on the 3-Day test set. A similar trend is observed in PCU models.

**Table 9:** Performance results of the models trained with combined multi-site data, tested with combined multi-site, SK-Hospital, KK-Hospital data.

		Train: combined multi-site		
Model	Scenario	Test: combined multi-site	Test: SK-Hospital	Test: KK-Hospital
1-Day	GH	0.79 (0.76, 0.82)	0.76 (0.70, 0.81)	0.83 (0.78, 0.87)
	PCU	0.66 (0.62, 0.70)	0.62 (0.56, 0.67)	0.70 (0.65, 0.75)
2-Day	GH	0.87 (0.84, 0.89)	0.87 (0.83, 0.90)	0.86 (0.83, 0.90)
	PCU	0.76 (0.72, 0.79)	0.75 (0.70, 0.80)	0.77 (0.71, 0.82)
3-Day	GH	0.90 (0.87, 0.93)	0.91 (0.87, 0.94)	0.89 (0.84, 0.93)
	PCU	0.79 (0.75, 0.83)	0.79 (0.73, 0.84)	0.79 (0.73, 0.85)

Examining the performance of the multisite models in the separate hospital datasets shows that the performance in the SK-Hospital is lower than in the KK-Hospital by approximately 10% on day 1 for the GH and PCU settings. The performance for the other days differs only slightly if at all. Fig 3 shows the number of features, as well as the sensitivity, specificity, and ROC curves, for the 1- to 3-day models in the PCU and GH settings.

**Fig 4:**
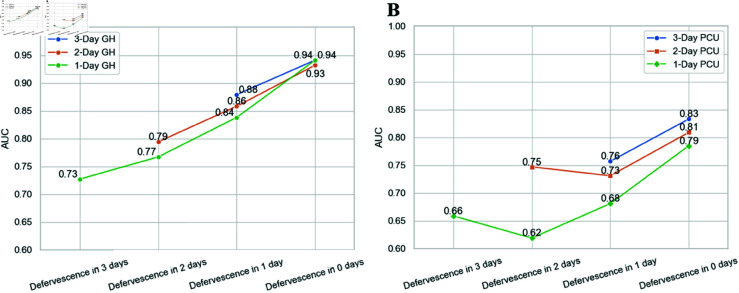
Evaluation of models with test data aligned by the day of defervescence in GH (A) and PCU (B) settings. Defervescence in 0 days means that the day of defervescence is the same as the day the prediction is made, whereas Defervescence in x days means that the prediction is done x days before defervescence.

In addition to evaluating the models using test sets organized by the study day, which is the day since presenting for medical care, an evaluation was performed considering the time relative to the day of defervescence, which is the day on which plasma leakage occurs in patients with DHF [24]. The objective of this analysis was to evaluate whether the prediction accuracy was influenced by the timing of data collection relative to the onset of defervescence (Fig 4). Thus, we split the test dataset into groups based on the number of days from defervescence. We note that information about defervescence was not included in the models. The figure illustrates how the models perform with data collected at varying intervals before the day of defervescence. The models do not contain or rely on any information on the actual day of defervescence.

Since there were very few patients who had their data collected earlier than three days before defervescence, the analyses were performed starting with defervescence in 3 days. The 1-day models need only one day of data, so they gave us four predictions: for defervesencen in 3 days, 2 days, 1 day, and 0 days. The 2-day models gave us three predictions and the 3-day models gave us two predictions.

For the GH setting, the accuracies of 1-Day, 2-Day and 3-Day models are similar for predictions on the day of defervescence (AUC values range from 0.93 to 0.94). The AUC values decrease and diverge as the timing of the data collection relative to the day of defervescence increases. The AUC of the 1-Day model drops to 0.72 for data collected three days before defervesence. On the other hand, for the PCU setting, 1-Day, 2-Day and 3-Day models have quite different AUC values even on the day of defervescence, with the 3-Day model being most accurate (0.83) and the 1-Day model the least (0.66). The AUC of the 3-Day model drops to 0.62 for a time horizon of 3 days. Prediction in both the GH and the PCU settings is more difficult for patients in the early days of their illness. Since defervescence typically occurs three to seven days after fever onset [24], applying our models to patients on the first or second day of fever may lead to low accuracy. This is likely due to limited clinical differentiation early in the disease. During the first days, patients with DF and DHF often present with similar features, making it difficult to distinguish between the two. However, these differences become more pronounced around the day of defervescence. For example, platelet nadir and hemoconcentration (hematocrit increased more than 20% above baseline) are generally observed at this stage.

### Generalizability

For predictive models to be useful in the prognosis of severity of dengue, they must work in real-world settings and contribute to better patient outcomes. A key factor in this is generalizability, which refers to the ability of a model to perform well at a site or on a population other than that from which the training data was taken. In this study, data were collected from two geographically distinct sites in Thailand, where standardized protocols for patient care and data collection were followed. However, differences in genetic background and slight variations in clinical measurements could lead to discrepancies between data sets.

To evaluate generalizability, models trained on data from one hospital were tested on the other, alternating test sets during bootstrapping. This analysis was performed for all three study day models in both GH and PCU settings. The results in Table 10 show that models trained for a given hospital consistently perform better on the test set for that hospital than do models trained for the other hospital. For the SK-hospital test set, the models trained using SK-hospital data all perform better than the models trained using KK-hospital data. The same pattern holds for the KK-hospital test set.

**Table 10:** AUC values of models trained on data from each of the two hospitals and tested on data from the same hospital and from the other hospital.

Model	Scenario	Test: SK-hospital		Test: KK-hospital	
		Train: SK-hospital	Train: KK-hospital	Train: KK-hospital	Train: SK-hospital
1-Day	GH	0.75 (0.70, 0.79)	0.74 (0.68, 0.79)	0.83 (0.78, 0.87)	0.80 (0.75, 0.84)
	PCU	0.62 (0.56, 0.68)	0.57 (0.50, 0.63)	0.72 (0.66, 0.78)	0.62 (0.55, 0.68)
2-Day	GH	0.85 (0.81, 0.89)	0.83 (0.79, 0.88)	0.86 (0.81, 0.90)	0.84 (0.80, 0.88)
	PCU	0.73 (0.68, 0.78)	0.67 (0.60, 0.73)	0.77 (0.72, 0.81)	0.71 (0.65, 0.76)
3-Day	GH	0.90 (0.86, 0.94)	0.86 (0.81, 0.91)	0.89 (0.85, 0.93)	0.86 (0.81, 0.91)
	PCU	0.78 (0.72, 0.84)	0.76 (0.70, 0.83)	0.76 (0.69, 0.82)	0.74 (0.67, 0.81)

Statistical significance of the difference in AUC values between models trained and tested on the same hospital versus models trained on one and tested on the other was performed using a one-tailed Mann-Whitney U test. This tests whether the AUCs of the same site training/testing are significantly higher than the AUCs of the cross site training/testing. Table 11 show the significance of the differences in AUCs between columns one and two and between columns three and four in Table 10. All differences in performance are significant.

**Table 11:** P-values for the differences in AUCs between same-site and cross-site training/testing using the one-tailed Mann-Whitney U test.

Model	Scenario	Test SK-Hospital	Test KK-Hospital
		Train: SK-Hospital Vs. Train: KK-Hospital	Train: KK-Hospital Vs. Train: SK-Hospital
1-Day	GH	< 0.001	< 0.001
	PCU	< 0.001	< 0.001
2-Day	GH	< 0.001	< 0.001
	PCU	< 0.001	< 0.001
3-Day	GH	< 0.001	< 0.001
	PCU	< 0.001	< 0.001

## Discussion

The prognosis of dengue in its early stages is challenging, emphasizing the need for timely identification and management to prevent complications. The initial phase of fever, typically spanning days 1 to 4 of illness, is commonly called the febrile phase. Sangkaew *et al*. [25] identified several factors associated with the febrile stage progressing to DHF, including age, sex, nutritional status, weight, vomiting, abdominal pain and tenderness, bleeding manifestations, platelet count, hematocrit levels, aminotransferase levels, immune status, and specific serotypes.

Our findings are consistent with theirs regarding abdominal pain and clinical fluid retention, which are inferred in our models through proxy variables such as fluid intake-output differences, abdominal circumference, and liver size. The study also highlighted clinical signs such as vomiting, abdominal pain and tenderness, bleeding, and clinical fluid accumulation as significantly correlated with a higher risk of DHF.

One of the top-ranked features in our study, platelet count, aligns with findings from previous studies using the 1997 WHO classification for pediatric patients. Aminotransferase levels (AST and ALT) were also identified as important features, confirming their well-established link to DHF as noted by Sangkaew *et al*. While we classified patient severity based on the 1997 WHO guidelines [3], several features identified in our models also correspond to the 2009 WHO guidelines for severe dengue warning signs [4]. For example, in the 1-Day GH model (Table 5), four of the eight WHO 2009 warning signs emerged as significant predictors: hepatomegaly (indicated by liver size), clinical fluid accumulation (captured by fluid intake-output differences), rising hematocrit levels (HCT), and a rapid drop in platelet count. These results suggest that plasma leakage is a critical factor in dengue progression, though its significance is interpreted differently under the 1997/2011 and 2009 WHO guidelines.

The 1997/2011 WHO guidelines treat plasma leakage as the main criterion for diagnosing or classifying DHF. Early detection and monitoring are essential in these cases to guide fluid replacement and prevent hypovolemic shock. In contrast, the 2009 WHO guidelines broaden the criteria, treating plasma leakage as one of several warning signs for severe dengue (SD) rather than a defining feature. This approach classifies patients with other warning signs, even in the absence of plasma leakage, as having dengue with warning signs, which requires close monitoring and medical attention.

These differences shape how the models are applied in clinical settings. In facilities following the 2009 WHO guidelines, plasma leakage is less central to predictions, as the broader criteria rely on a wider range of warning signs. While the models include features such as abdominal pain and liver enlargement, which align with the 2009 guidelines, these signs do not necessarily indicate plasma leakage. In this context, the models act as a supportive tool to complement clinical evaluations. For facilities using the 1997/2011 WHO guidelines, where plasma leakage is the key trigger for intervention, the models provide early predictions that help clinicians monitor and treat patients promptly. By providing early identification of plasma leakage, the models can supplement clinical decision-making and improve patient care, particularly in settings where plasma leakage is critical for managing severe cases.

The cross-site generalizability of learned medical decision support models is an understudied issue, which is important for the practical deployment of such models since the accuracy of a model may differ for clinical settings or geographic regions beyond those from which the original data came [26–28]. In their review, Low and colleagues [29] highlighted the need for multicenter studies for developing prediction models that could strengthen the generalizability of the study findings. Yet, McDermott and colleagues found that only approximately 23% of ML-based healthcare papers used multiple datasets [26]. Inadequate model generalizability often hinders the practical implementation of machine learning and deep learning models in real clinical settings.

Data from different clinical sites can differ due to differences in how clinical features are defined or collected and due to differences in patient populations [30]. Here, we examined whether group-level performance of prognostic models varies significantly when applied to hospitals or geographies different from the ones in which they are developed. Although our data came from a prospective cohort study in which the same data collection protocol was used at both hospital sites, a comparison of the feature rankings of the models for the two hospitals shows significant differences, as shown in Tables 12 and 13. These differences occur mainly in two types of features. The first is features that are somewhat subjective in nature or that are objective, but that may be defined or measured differently at different clinical sites, such as Abdominal circumference (Table 12: rank 3 for KK, rank 7 for SK; Table 13: rank 1 for KK, rank 6 for SK), Bleeding (Table 13: rank 10 for KK, rank 21 for SK), and Abdominal pain (Table 12: rank 20 for SK, not present for KK; Table 13: rank 3 for SK, rank 16 for KK). The other is hematological parameters, some of which appear in features for SK-Hospital but not for KK-Hospital. These include Monocyte (rank 23) and Band (rank 29) (Table 12).

**Table 12:**
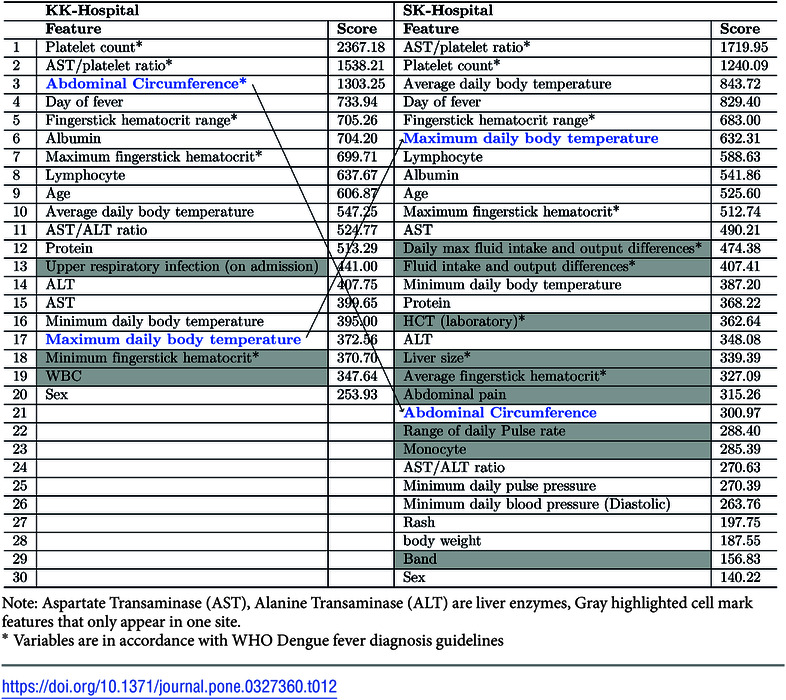
Selected features with feature importance scores for the 1-Day(GH) models trained with the two site-specific datasets.

Note: Aspartate Transaminase (AST), Alanine Transaminase (ALT) are liver enzymes, Gray highlighted cell mark features that only appear in one site.

*Variables are in accordance with WHO Dengue fever diagnosis guidelines

**Table 13:**
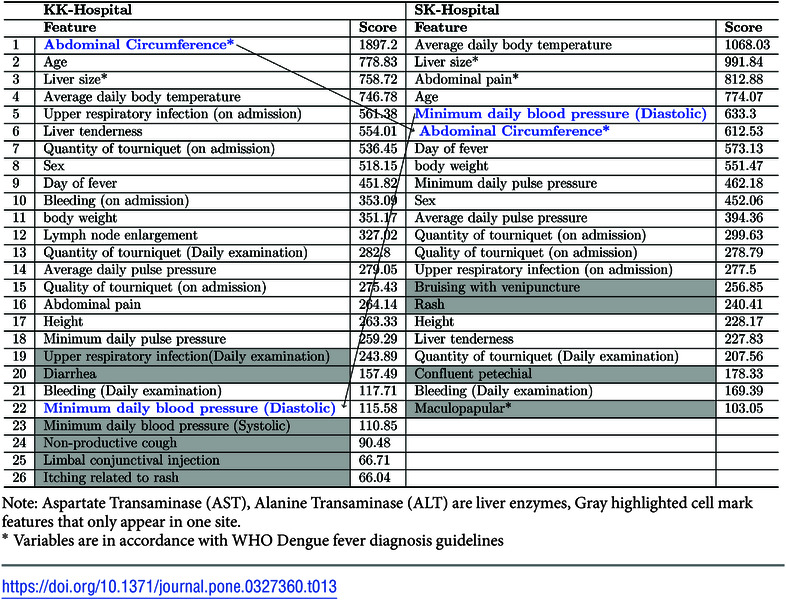
Selected features with feature importance scores for the 1-Day(PCU) models trained with the two site-specific datasets.

Note: Aspartate Transaminase (AST), Alanine Transaminase (ALT) are liver enzymes, Gray highlighted cell mark features that only appear in one site.

*Variables are in accordance with WHO Dengue fever diagnosis guidelines

Variations in features between our study sites are likely influenced by subjective factors from physical examinations and interview responses, as well as differences in measurement techniques and equipment. For instance, SK-Hospital used a digital sphygmomanometer, while KK-Hospital employed an analog one for measuring blood pressure, resulting in more detailed measurements at SK. Standardizing operating procedures and conducting routine refresher training can help minimize such deviations.

The differences in hematological parameters, specifically the levels of Monocyte and Band (Table 12), are unlikely to result from measurement or procedural inconsistencies. Instead, they may be linked to genetic differences between the two populations. For instance, the prevalence of thalassemia carriers varies significantly across Thailand, with over 30% of Northeastern Thais and approximately 10% of Southern Thais being hemoglobin E carriers [31]. Hemoglobin E and β-thalassemia carriers may pass on severe hemoglobin E/β-thalassemia to their offspring. Additionally, α-thalassemia is more prevalent in northern Thailand compared to the southern region [32].

Thalassemia has been associated with altered WBC functionality, including impaired vitality and function of neutrophils [33], lymphocytes [34–36], and monocytes [37]. A study on β-thalassemia carriers reported significantly reduced WBC levels, potentially reflecting a diminished inflammatory response, which might explain its protective effect against cardiovascular disease [38]. Dysfunctional innate immune responses, including impaired inflammation, have been linked to severe dengue or dengue hemorrhagic fever [39]. These observations suggest that differences in Monocyte and Band levels in response to dengue infection in this study may relate to impaired WBC function in thalassemia carriers. Given these observations, participants’ genomes in this study are being sequenced as part of the Genomics Thailand project (https://genomicsthailand.com/). This analysis aims to identify genetic factors, such as thalassemia traits or other variables, that might explain the variation in WBC responses to dengue infection.

The differences in features selected for our prognostic models align with findings by Pongpan and colleagues, who reported significant regional variability in clinical data. They also observed reduced accuracy when applying a scoring system developed in a prior study [9] to patients from different regions. This reduced accuracy was attributed to differences in patient severity between the development and validation datasets, highlighting the impact of population variability on model performance.

Differences in laboratory features due to population background (such as the prevalence of thalassemia traits discussed above) are significant and must be accounted for in clinical analysis and model development. However, inconsistencies in features derived from vital signs and physical examinations might arise due to differences in measurement or evaluation procedures. Standardization and training can help reduce these inconsistencies and improve data reliability across sites.

Our study utilized a dataset collected over 17 years, including virus isolation for all cases. This dataset spans multiple outbreaks, includes all four dengue serotypes, and distinguishes between primary and secondary infections. Its long-term nature provides a unique opportunity to analyze trends and patterns that are challenging to capture in studies with shorter time frames.

Using this dataset, we developed predictive models based on data from children under 15 years old, collected at two hospitals in Thailand, where dengue is prevalent among pediatric populations [3,40,41]. The models aim to identify patients at risk of developing DHF early in their illness, supporting clinicians in allocating limited resources effectively in dengue-endemic areas. Predictive tools for dengue are typically designed for well-resourced settings, but there is increasing interest in tools suitable for resource-limited environments such as primary care units [42,43]. PCUs are particularly relevant in dengue-endemic countries, as they are often the first point of care for most patients. In this study we thus developed models for both GH and PCU settings. Designed without reliance on laboratory-based variables, the PCU models are practical for outpatient care, especially during seasonal outbreaks that strain healthcare systems.

To ensure applicability across different settings, we assessed the models in both resource-limited and general hospital environments. By combining long-term data with evaluations across multiple sites, this study offers reliable and adaptable results compared to studies confined to shorter or single-setting datasets. The models perform comparably to or better than those in existing studies that address similar challenges.

Despite this limitation, the models provide significant value in settings with limited access to experienced clinicians or advanced diagnostic tools. For example, in countries with emerging dengue outbreaks, such as Pakistan, where the case fatality rate was 0.25% in 2022 [44], these tools could assist in early detection and intervention. Even in Thailand, where the fatality rate in cases is below 0. 1% [45], the models could support healthcare providers in rural or understaffed areas and help newly trained personnel recognize severe cases.

Although the models focus on practical features that are suitable for resource-limited settings, we also considered additional diagnostic tools, such as NS1 testing and serology, which are often recommended for improving early dengue detection. However, these were not included in our study due to several practical and contextual limitations. The NS1 rapid test was not available in Thailand at the start of the study in 2001, and older samples that could have been suitable for NS1 testing were depleted due to usage in other studies. Furthermore, NS1 testing has inherent limitations: its sensitivity ranges from 55% to 82% [46], leading to a risk of false negatives, and it typically provides only binary results (positive/negative) without quantification, which limits its utility for severity prediction. Although NS1 levels are associated with disease severity [47], its practical application in predicting outcomes remains constrained. Timing is another consideration. Paranavitane *et al*. [48] found an association between NS1 detection on days 5–6 of illness and severe dengue. However, our study focuses on fever subsidence and plasma leakage, which generally occur between days 3–7 [2], with patients enrolled on day 4 or earlier. As a result, these findings may not be directly applicable to our cohort.

Similarly, serology, particularly the detection of secondary infections, is strongly associated with DHF but presents practical challenges. Accurate serology testing requires convalescent samples collected one to two weeks after fever onset, using anti-dengue IgG and IgM capture ELISA [18]. Since this timing falls after defervescence or plasma leakage, serology cannot reliably predict these outcomes within the scope of our study. Our findings have clinical relevance, given that many dengue cases in Thailand and neighboring Southeast Asian regions occur in children [3,40,41]. However, this includes only patients seeking medical attention; our study does not include those with milder diseases or asymptomatic infections. With only a Thai pediatric population covered, this study was ethnographically homogenous; applying the findings in clinical settings to ethnically diverse populations, adult patients, or different regions may not be appropriate.

## Conclusions and future work

Regions with high dengue transmission often experience seasonal outbreaks that can overwhelm healthcare capacities. An early prediction tool to identify patients at risk of progressing to DHF could optimize the use of limited hospital resources in dengue-endemic areas. Our predictive models, developed for resource-limited settings, do not require laboratory-based variables, making them suitable for facilities with minimal resources or inexperienced staff. These models can aid outpatient fever management and inpatient monitoring, though their generalizability needs careful consideration. Further research is necessary to address variability in clinical data and patient sub-populations, ensuring broader applicability.

In our study, we developed models using standard machine learning methods and features commonly available in clinical settings, derived from a prospective study. The manuscript includes exploratory data analysis, detailed data set statistics, and a list of features along with their importance scores. These resources support replication and encourage researchers to build their own models using similar data. To address data variability between regions, we evaluated the generalizability of sites across between two hospitals. The results are particularly relevant for the development of prediction models for children with suspected dengue infections. We plan to deploy these models as a mobile application for bedside use, starting with manual testing and later integrating the tool with electronic medical records.

Although our study used complete data for prediction, real-world scenarios may require inference at different times, often before all information is available. Developing models that handle partial data effectively is a priority for future work. In addition, our goal is to develop explainable models to improve the interpretability of computational processes and support clinical decision-making.

## Supporting information

S1 File Time course of the numerical variable from study day one to five in two groups (DHF and Non-DHF). (PDF)

S2 File Time course of the binary variable from study day one to three in two groups (DHF and Non-DHF). (PDF)

S3 File Odd ratio. (PDF)

S4 File Features in five categories. (PDF)

S5 File Feature selected with feature importance scores for the 2-Day (GH), 3-Day (GH), 2-Day (PCU) and 3-Day (PCU) models trained with the two site-specific datasets. (PDF)

S6 File Core features for all models in combined multisite. (PDF)

S7 File Results for generalization experiments. (PDF)

S8 File The patient distribution by PCR and ELISA results. (PDF)

S9 File Time course of numerical variable from two to seven days after fever onset in two groups (DHF and Non-DHF). (PDF)

S10 File Time course of binary variable from two to seven days after fever onset in two groups (DHF and Non-DHF). (PDF)

S11 File Numeric variables from symptoms and biological parameters with medians (interquartile ranges) and odds ratios comparing DHF and Non-DHF categories, presented through day-by-day analysis. (PDF)

S12 File Numeric variables from symptoms and biological parameters with medians (interquartile ranges) and odds ratios comparing Non-DSS and DSS categories, presented through day-by-day analysis. (PDF)
