# Correction: Prognostic prediction of dengue hemorrhagic fever in pediatric patients with suspected dengue infection: A multi-site study

**DOI:** 10.1371/journal.pone.0344487

**Published:** 2026-03-09

**Authors:** Myat Su Yin, Peter Haddawy, Panhavath Meth, Araya Srikaew, Chonnikarn Wavemanee, Saranath Lawpoolsri Niyom, Kanokwan Sriraksa, Wannee Limpitikul, Preedawadee Kittirat, Nasikarn Angkasekwinai, Oranich Navanukroh, Arunee Mapralub, Supansa Pakdee, Chotika Kaewpuak, Nattaya Tangthawornchaikul, Prida Malasit, Panisadee Avirutnan, Dumrong Mairiang

In the Ethics Statement section, there is an error in the fourth sentence. The correct sentence is: The cohort study was approved by the Institutional Review Board (IRB) and Ethics Committee for Human Subjects at the Faculty of Medicine Siriraj Hospital, Mahidol University, Bangkok, Thailand (approval number 156/2002, 349/2550(EC1) and 632/2559 (EC2)) the Ethical Review Committee for Research in Human Subjects of the Ministry of Public Health (ECMOPH), Nonthaburi, Thailand (approval number 92/2550); the Ethical Review Committee for Research in Human Subjects of Khon Kaen Hospital, Khon Kaen, Thailand (approval number KE60108); and the Ethical Review Committee for Research in Human Subjects of Songkhla Hospital, Songkhla, Thailand (approval number 11/256).
