# Supplementary material for: Prognostic prediction of dengue hemorrhagic fever in pediatric patients with suspected dengue infection: A multi-site study
Source: PLoS One. 2025 Aug 4;20(8):e0327360. doi: 10.1371/journal.pone.0327360 (PMC12321061; doi:10.1371/journal.pone.0327360)
Supplement: S1 File — (PDF) [file pone.0327360.s001.pdf]

Supplement file 1

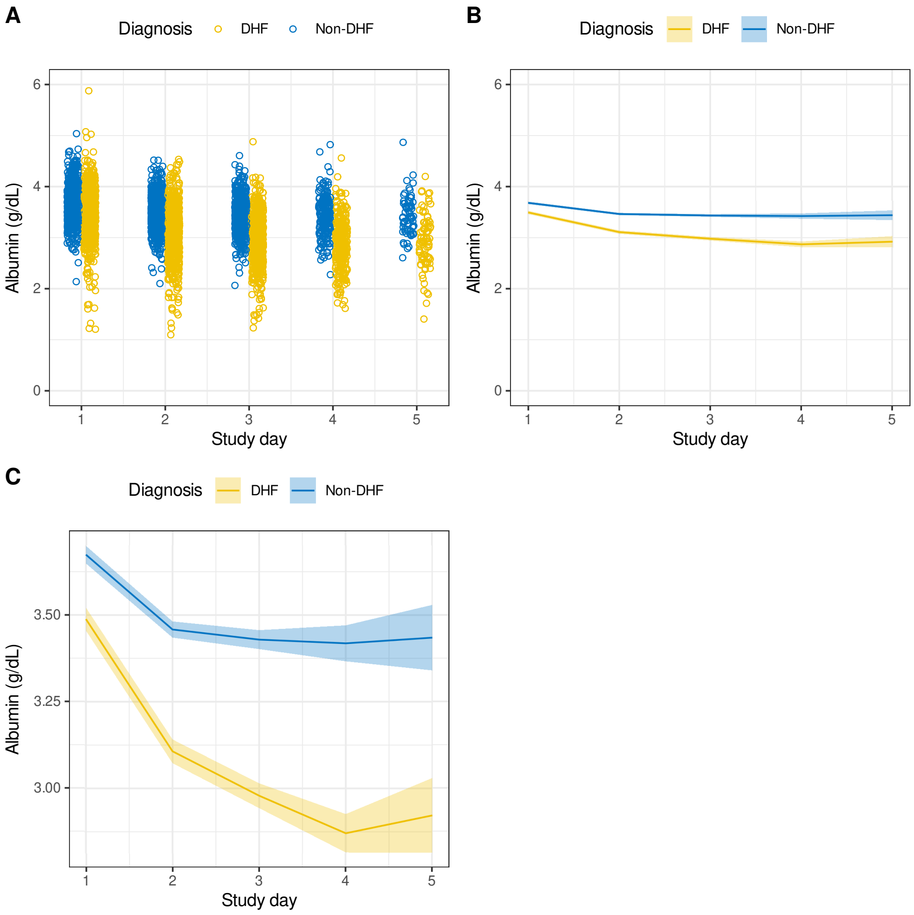

Fig. S1-2: Time course of `Albumin` variable from study day one to five in two groups (DHF and Non-DHF). The data are shown as raw values (A) and means by group by study day (B) with the shaded areas representing 95% confident intervals of the means. The mean values (B) are also zoomed in to show the difference and trajectories two groups (C).

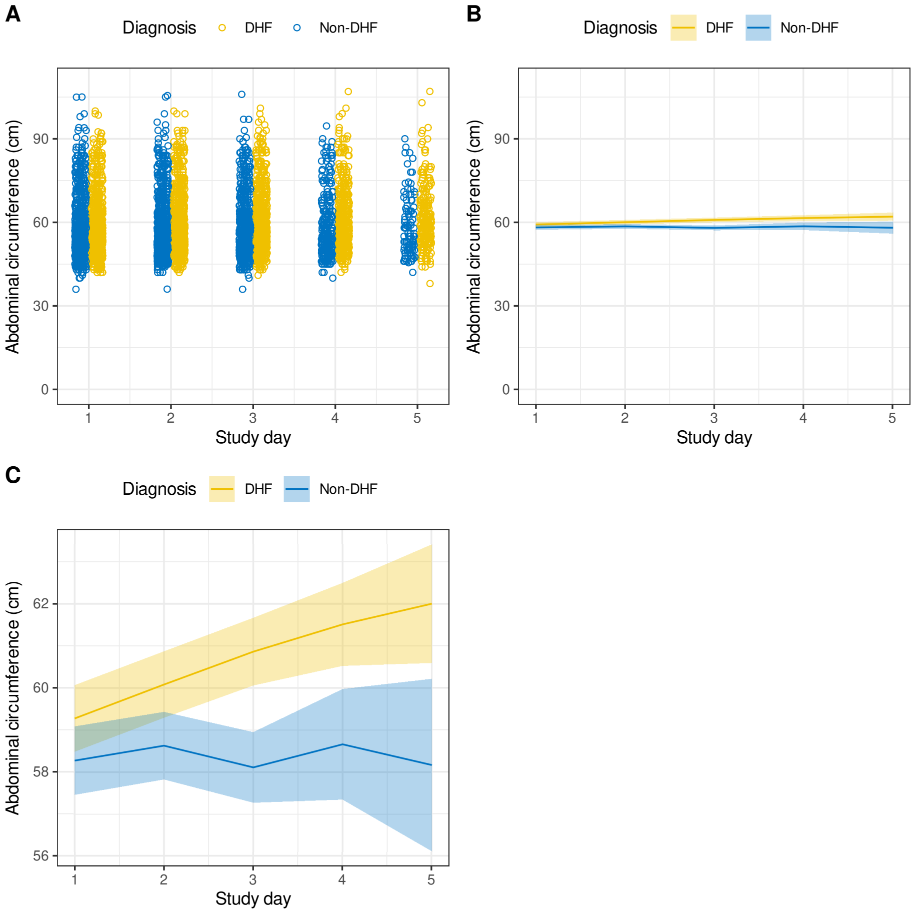

Fig. S1-1: Time course of `Abdominal Circumference` variable from study day one to five in two groups (DHF and Non-DHF). The data are shown as raw values (A) and means by group by study day (B) with the shaded areas representing 95% confident intervals of the means. The mean values (B) are also zoomed in to show the difference and trajectories two groups (C).

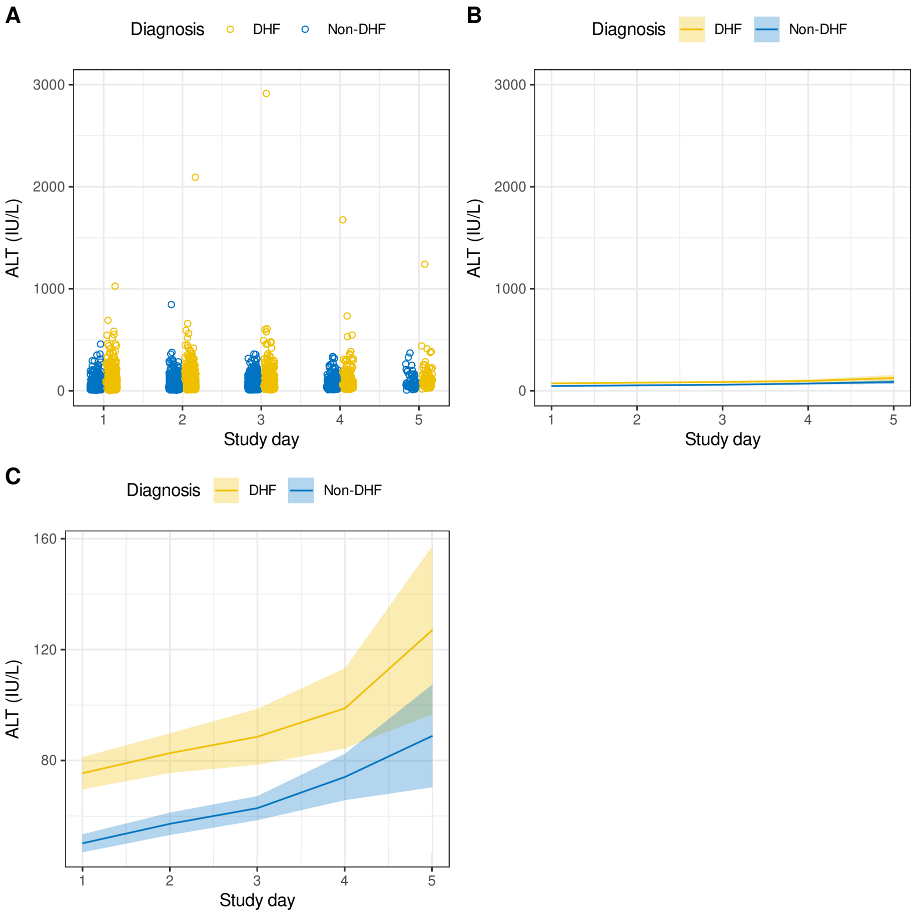

Fig. S1-3: Time course of `ALT` variable from study day one to five in two groups (DHF and Non-DHF). The data are shown as raw values (A) and means by group by study day (B) with the shaded areas representing 95% confident intervals of the means. The mean values (B) are also zoomed in to show the difference and trajectories two groups (C).

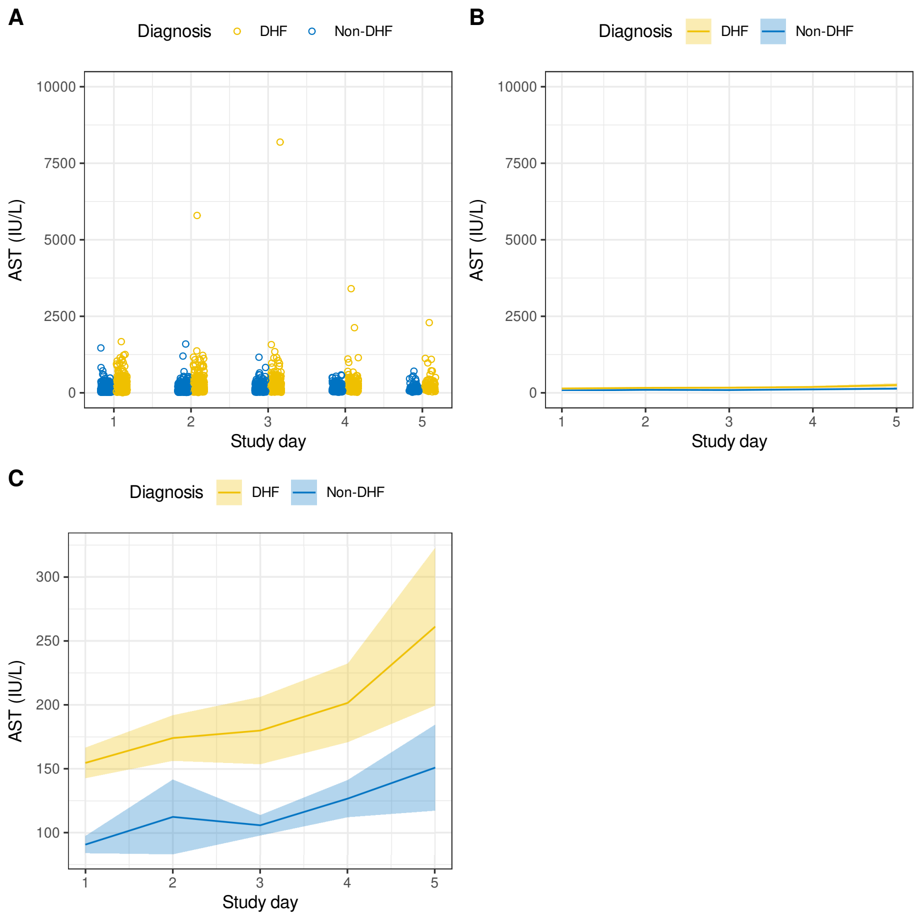

Fig. S1-4: Time course of `AST` variable from study day one to five in two groups (DHF and Non-DHF). The data are shown as raw values (A) and means by group by study day (B) with the shaded areas representing 95% confident intervals of the means. The mean values (B) are also zoomed in to show the difference and trajectories two groups (C).

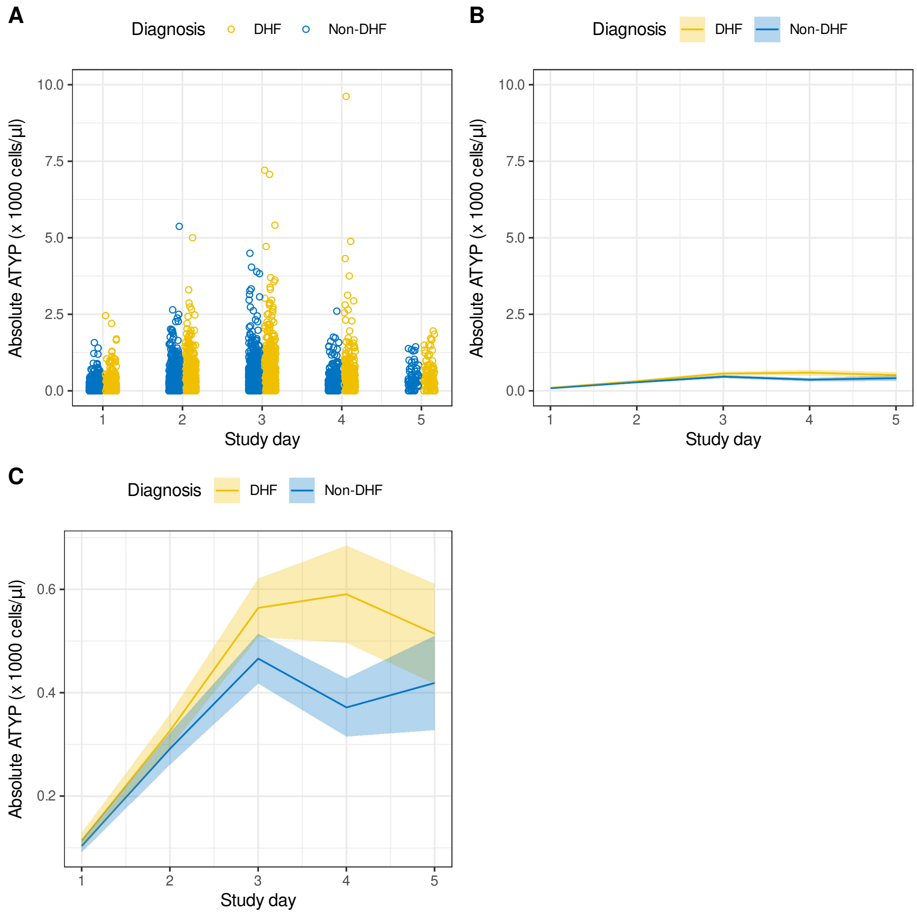

Fig. S1-5: Time course of `Atypical lymphocyte` variable from study day one to five in two groups (DHF and Non-DHF). The data are shown as raw values (A) and means by group by study day (B) with the shaded areas representing 95% confident intervals of the means. The mean values (B) are also zoomed in to show the difference and trajectories two groups (C).

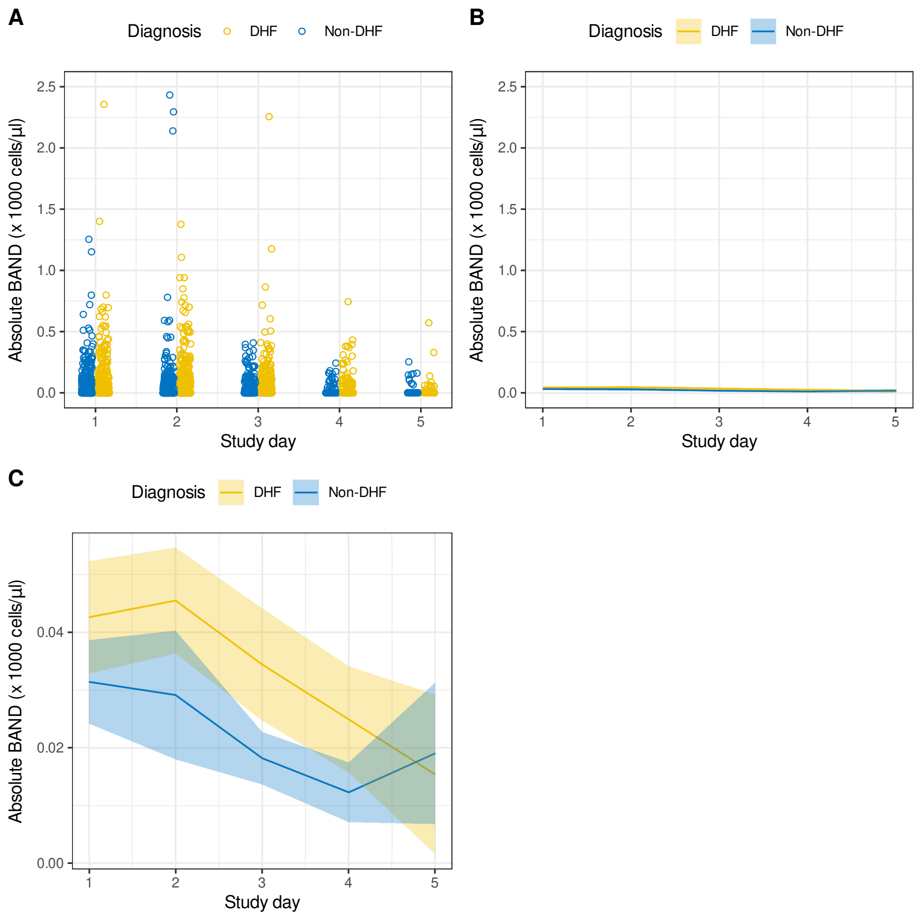

Fig. S1-6: Time course of `BAND` variable from study day one to five in two groups (DHF and Non-DHF). The data are shown as raw values (A) and means by group by study day (B) with the shaded areas representing 95% confident intervals of the means. The mean values (B) are also zoomed in to show the difference and trajectories two groups (C).

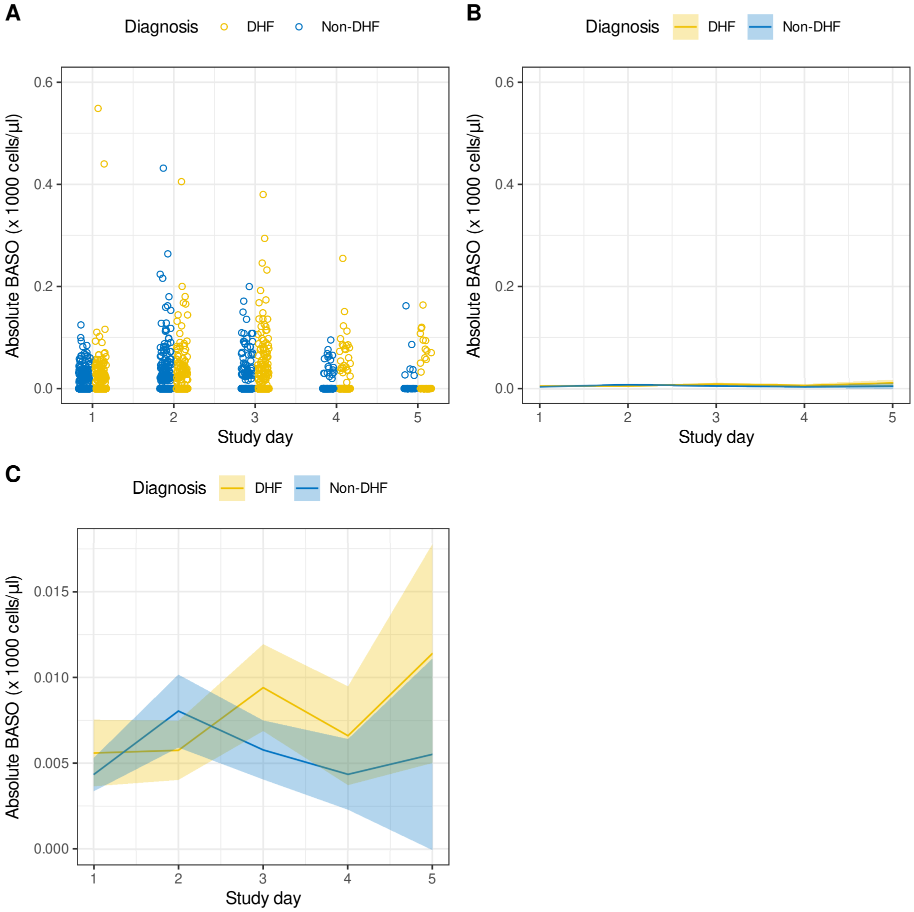

Fig. S1-7: Time course of `Basophil` variable from study day one to five in two groups (DHF and Non-DHF). The data are shown as raw values (A) and means by group by study day (B) with the shaded areas representing 95% confident intervals of the means. The mean values (B) are also zoomed in to show the difference and trajectories two groups (C).

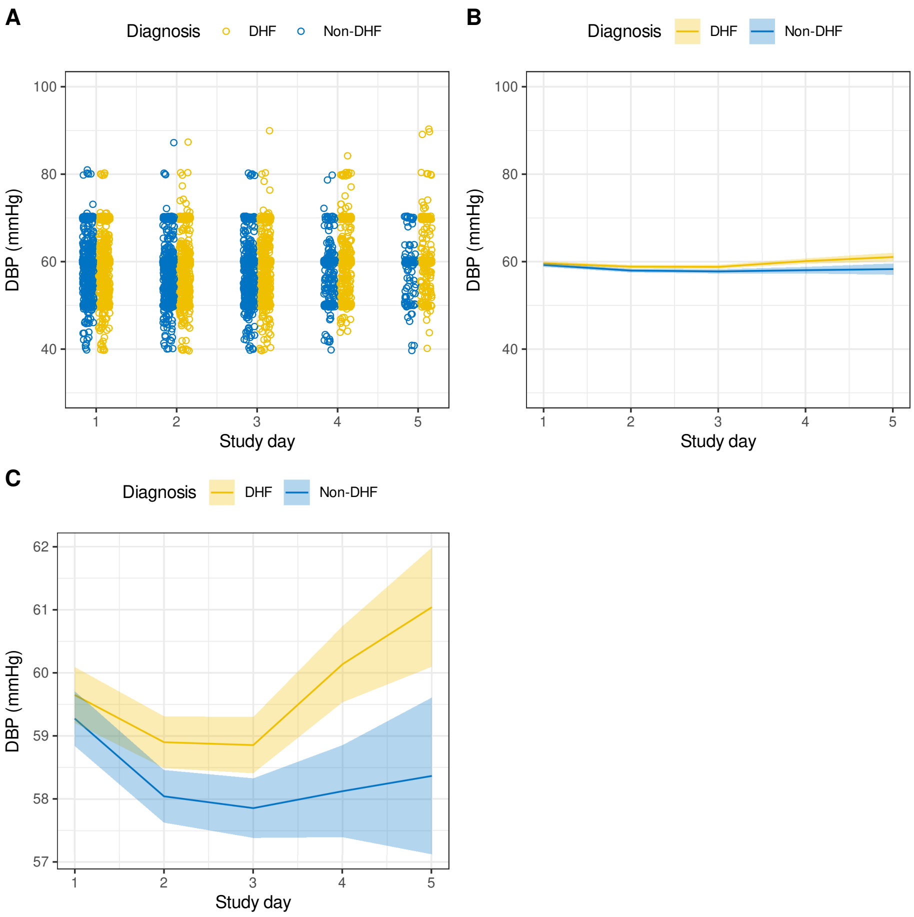

Fig. S1-8: Time course of `Minimum daily blood Pressure (Diastolic)` variable from study day one to five in two groups (DHF and Non-DHF). The data are shown as raw values (A) and means by group by study day (B) with the shaded areas representing 95% confident intervals of the means. The mean values (B) are also zoomed in to show the difference and trajectories two groups (C).

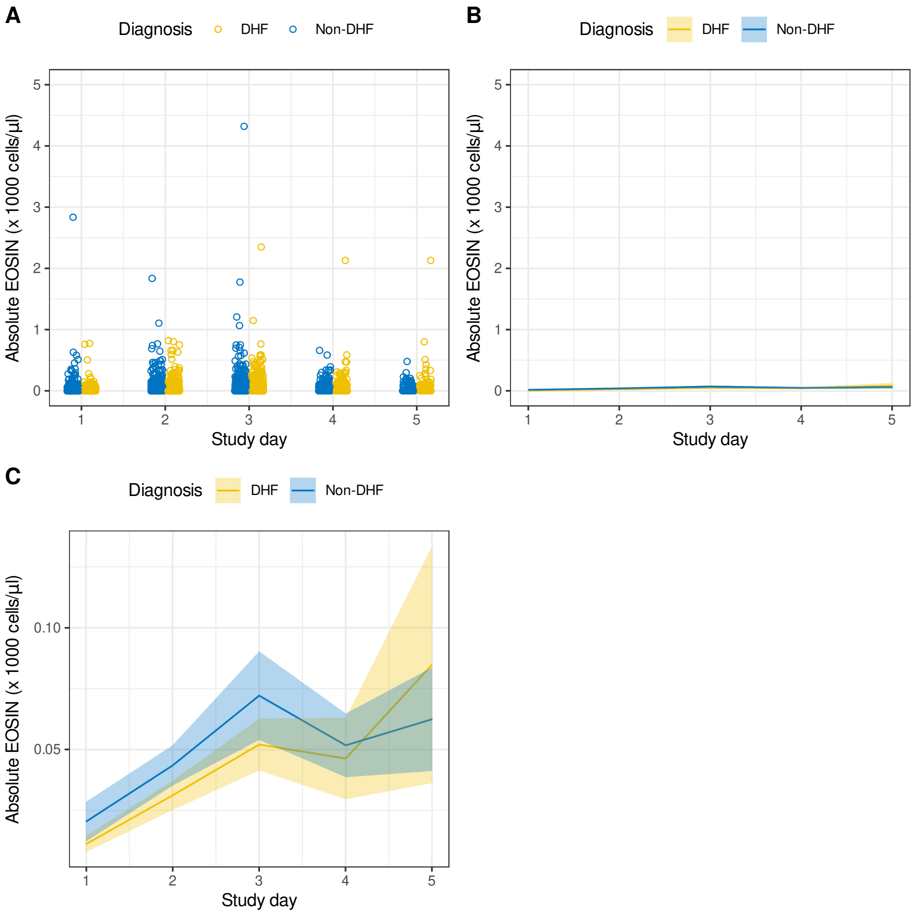

Fig. S1-9: Time course of `Eosinophil` variable from study day one to five in two groups (DHF and Non-DHF). The data are shown as raw values (A) and means by group by study day (B) with the shaded areas representing 95% confident intervals of the means. The mean values (B) are also zoomed in to show the difference and trajectories two groups (C).

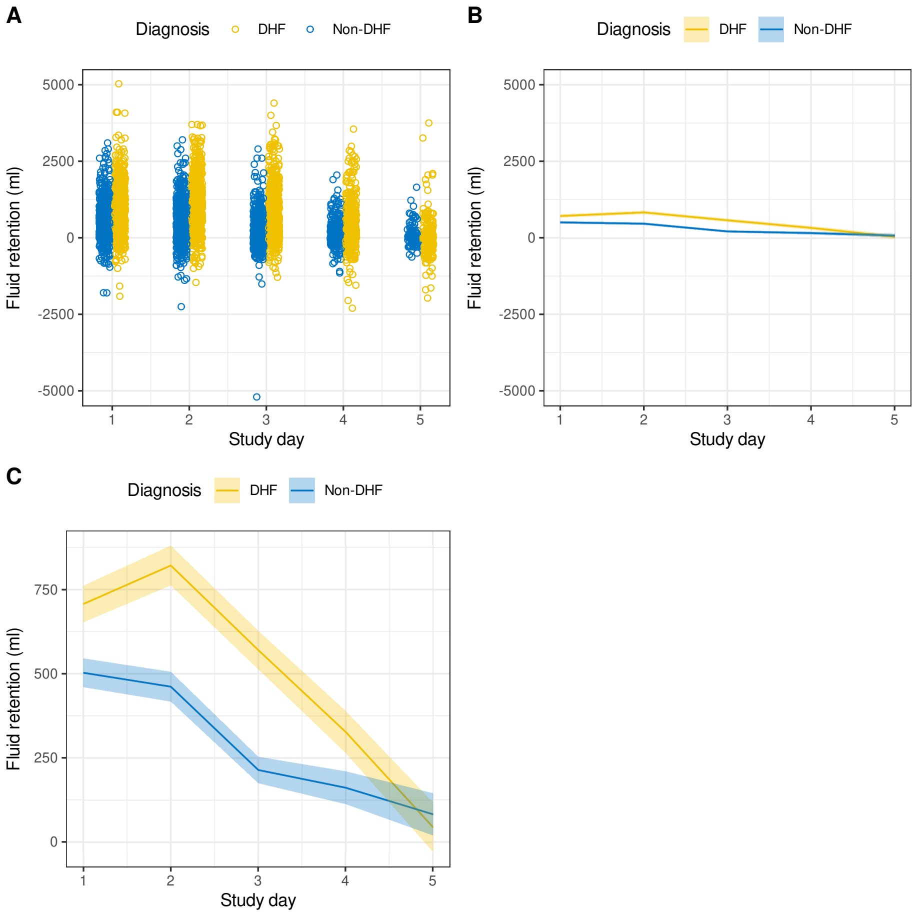

Fig. S1-10: Time course of `Daily maximum difference between intake and output` variable from study day one to five in two groups (DHF and Non-DHF). The data are shown as raw values (A) and means by group by study day (B) with the shaded areas representing 95% confident intervals of the means. The mean values (B) are also zoomed in to show the difference and trajectories two groups (C).

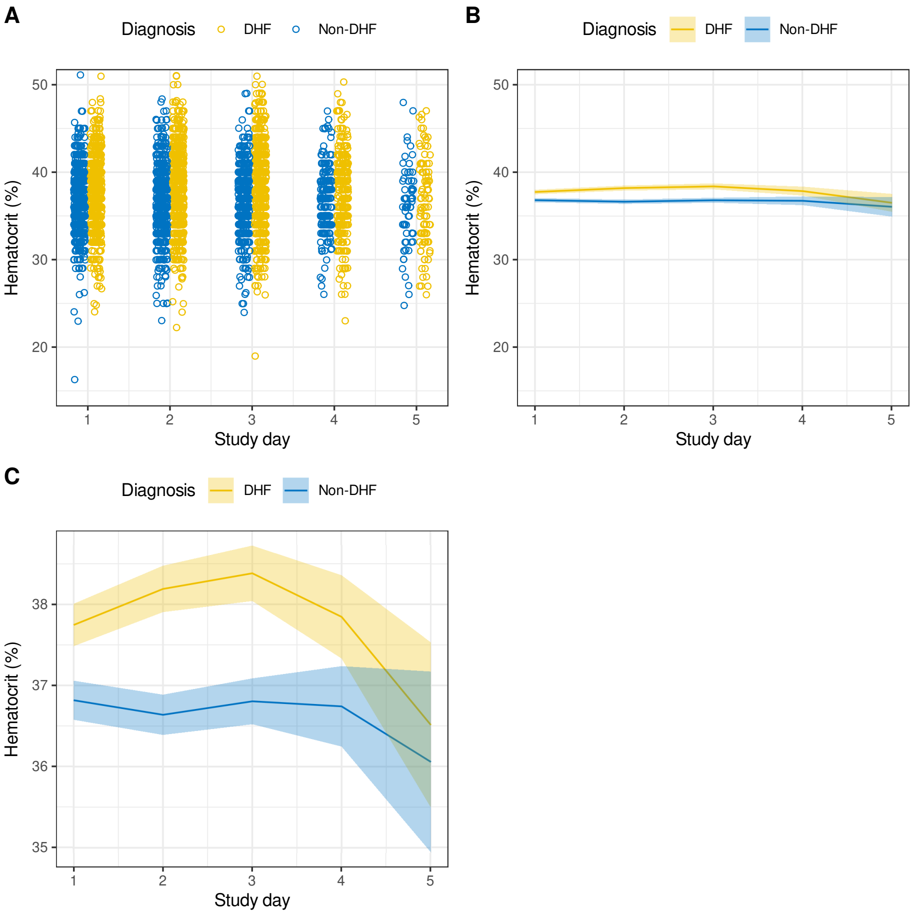

Fig. S1-11: Time course of `HCT (Lab)` variable from study day one to five in two groups (DHF and Non-DHF). The data are shown as raw values (A) and means by group by study day (B) with the shaded areas representing 95% confident intervals of the means. The mean values (B) are also zoomed in to show the difference and trajectories two groups (C).

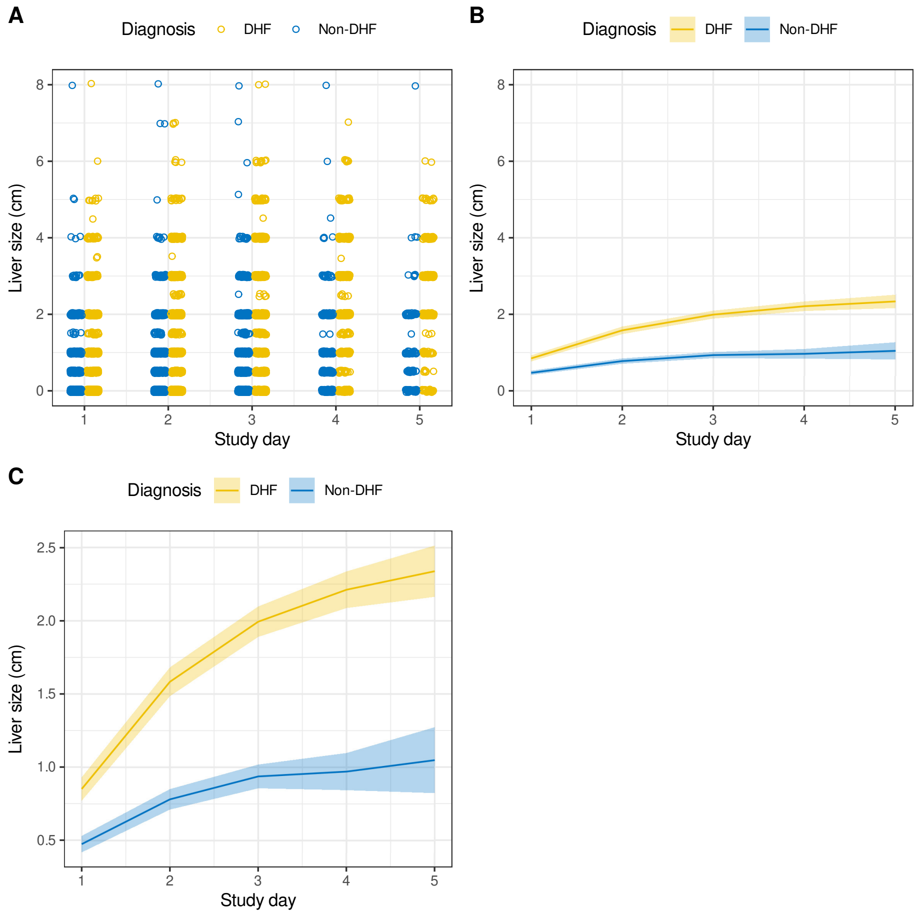

Fig. S1-12: Time course of `liver size` variable from study day one to five in two groups (DHF and Non-DHF). The data are shown as raw values (A) and means by group by study day (B) with the shaded areas representing 95% confident intervals of the means. The mean values (B) are also zoomed in to show the difference and trajectories two groups (C).

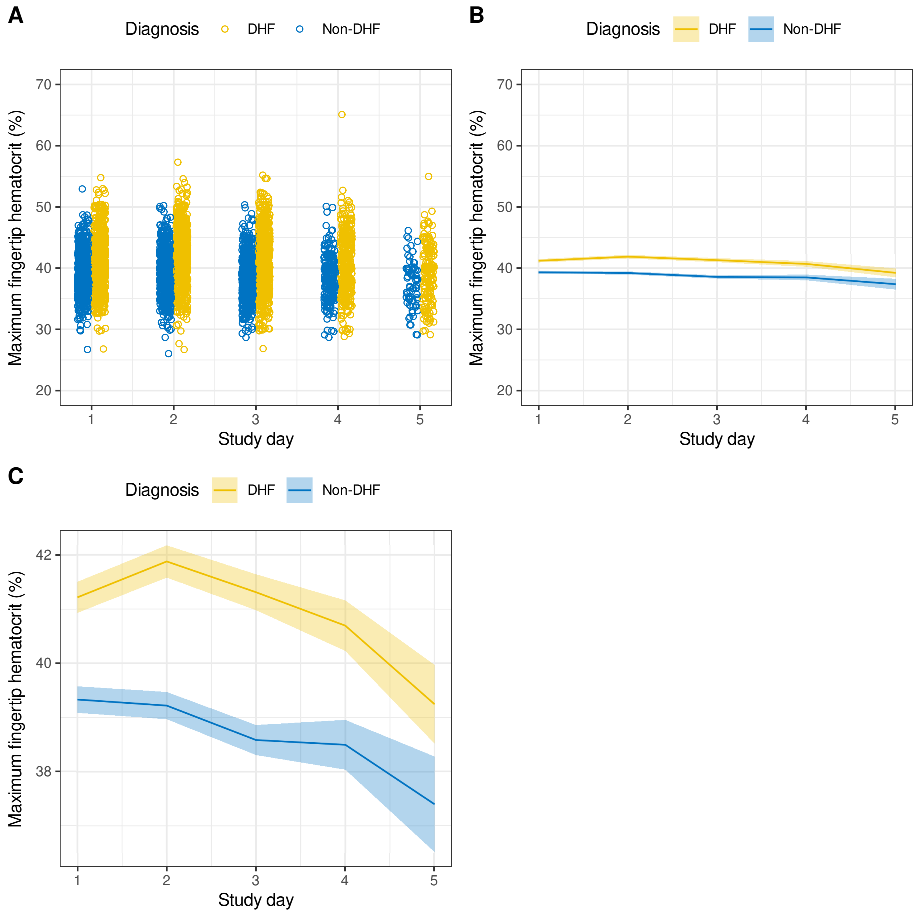

Fig. S1-13: Time course of `Maximum fingerstick hematocrit` variable from study day one to five in two groups (DHF and Non-DHF). The data are shown as raw values (A) and means by group by study day (B) with the shaded areas representing 95% confident intervals of the means. The mean values (B) are also zoomed in to show the difference and trajectories two groups (C).

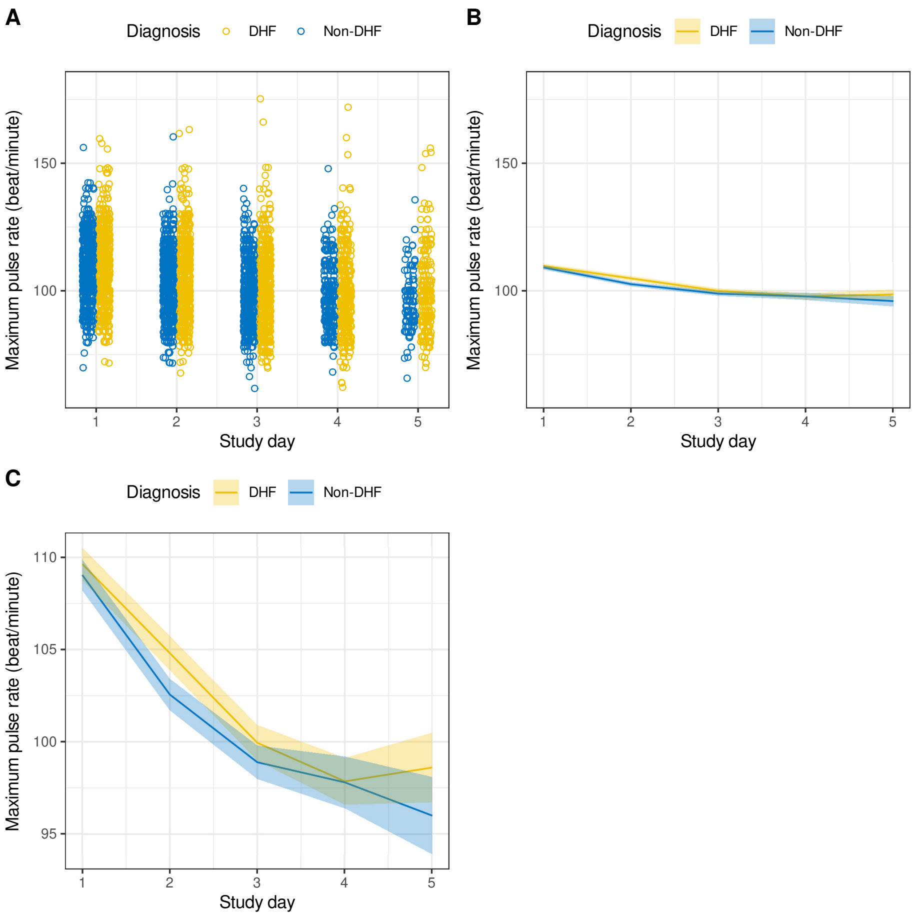

Fig. S1-14: Time course of `Maximum daily Pulse rate` variable from study day one to five in two groups (DHF and Non-DHF). The data are shown as raw values (A) and means by group by study day (B) with the shaded areas representing 95% confident intervals of the means. The mean values (B) are also zoomed in to show the difference and trajectories two groups (C).

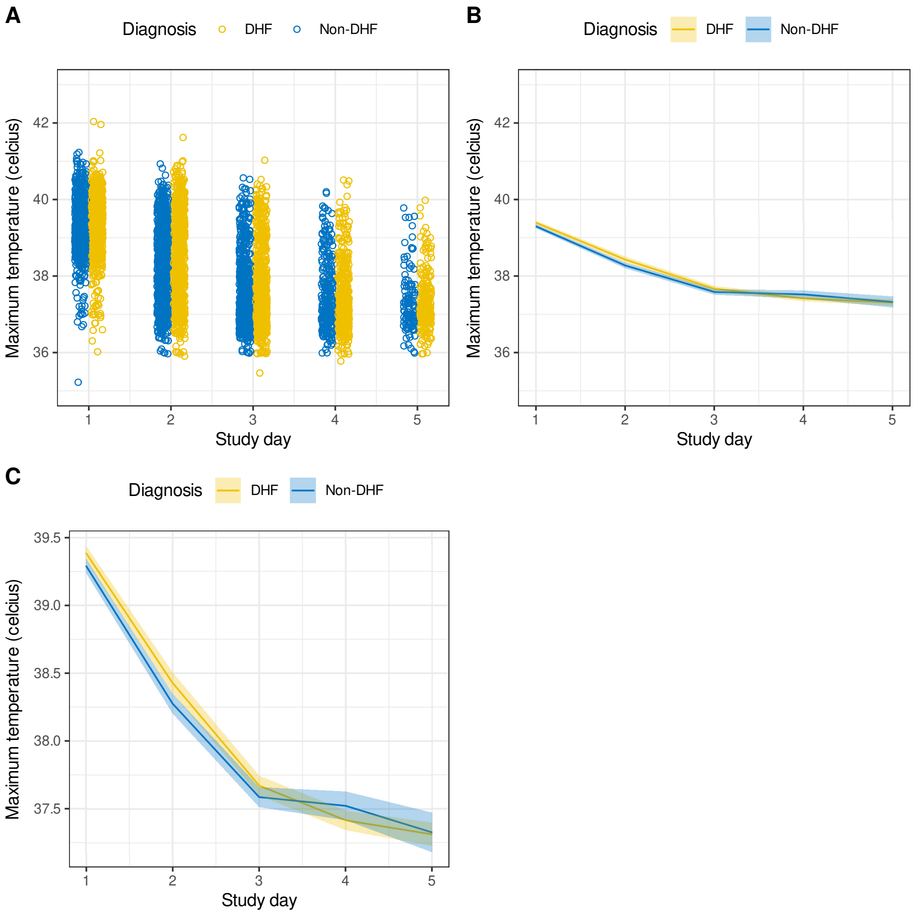

Fig. S1-15: Time course of `Maximum daily body temperature` variable from study day one to five in two groups (DHF and Non-DHF). The data are shown as raw values (A) and means by group by study day (B) with the shaded areas representing 95% confident intervals of the means. The mean values (B) are also zoomed in to show the difference and trajectories two groups (C).

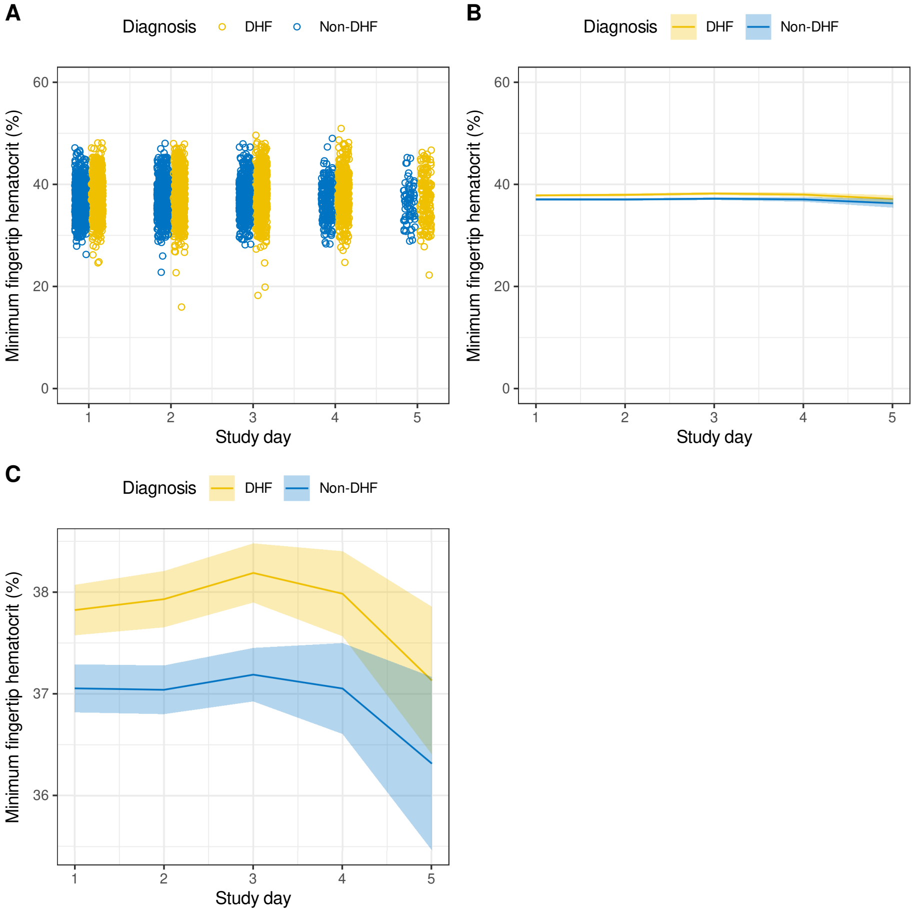

Fig. S1-16: Time course of `Minimum fingerstick hematocrit` variable from study day one to five in two groups (DHF and Non-DHF). The data are shown as raw values (A) and means by group by study day (B) with the shaded areas representing 95% confident intervals of the means. The mean values (B) are also zoomed in to show the difference and trajectories two groups (C).

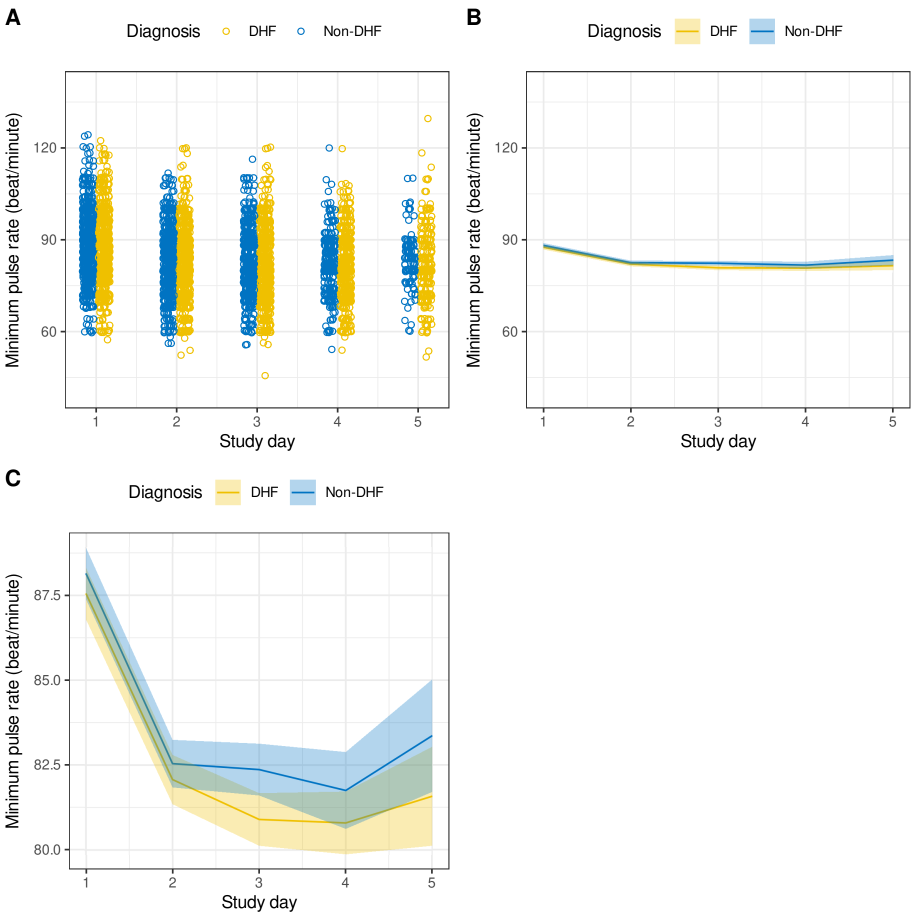

Fig. S1-17: Time course of `Minimum daily Pulse rate` variable from study day one to five in two groups (DHF and Non-DHF). The data are shown as raw values (A) and means by group by study day (B) with the shaded areas representing 95% confident intervals of the means. The mean values (B) are also zoomed in to show the difference and trajectories two groups (C).

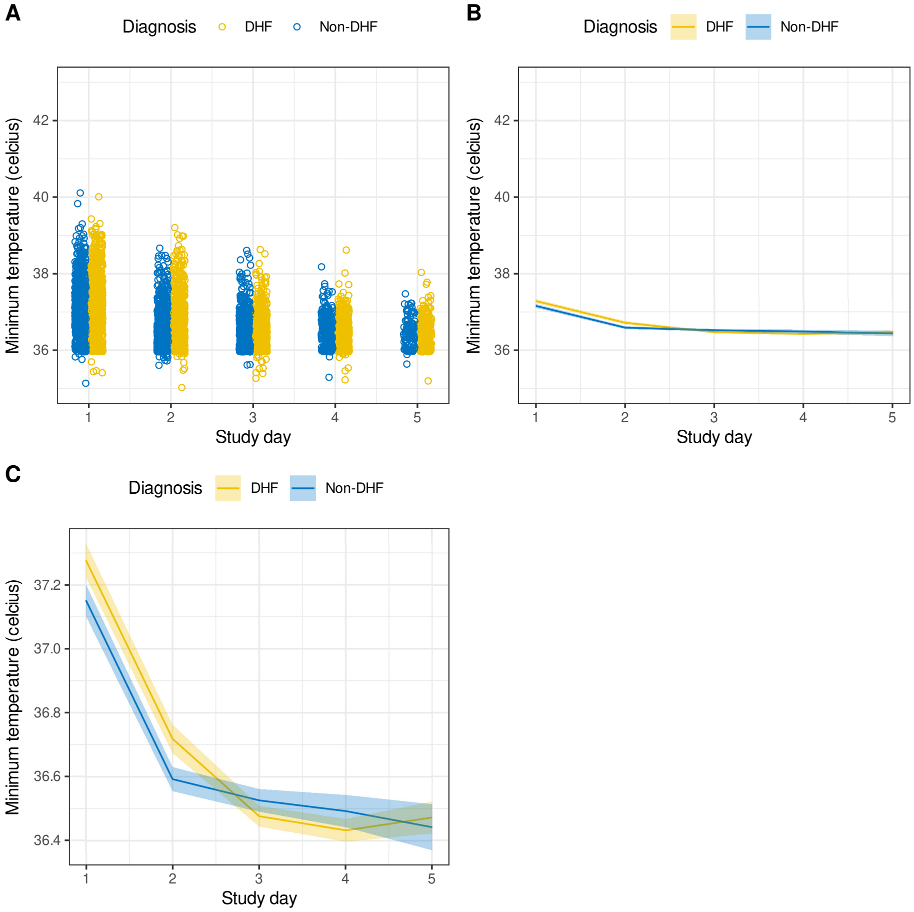

Fig. S1-18: Time course of `Minimum daily body temperature` variable from study day one to five in two groups (DHF and Non-DHF). The data are shown as raw values (A) and means by group by study day (B) with the shaded areas representing 95% confident intervals of the means. The mean values (B) are also zoomed in to show the difference and trajectories two groups (C).

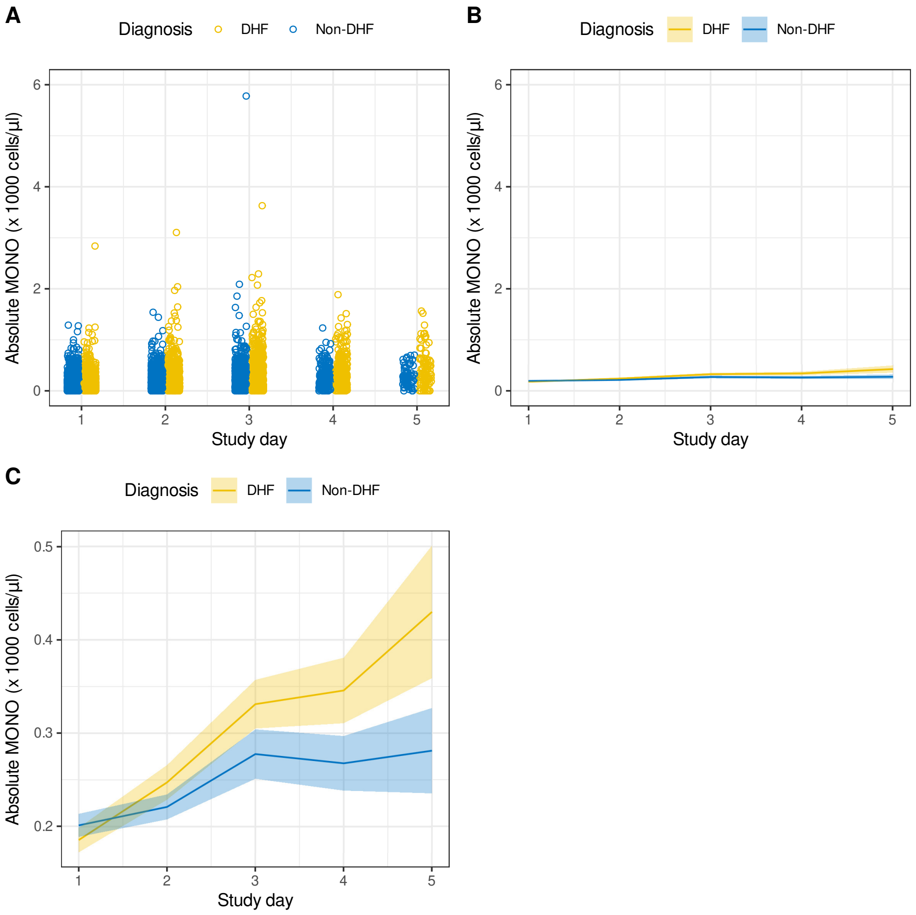

Fig. S1-19: Time course of `Monocyte` variable from study day one to five in two groups (DHF and Non-DHF). The data are shown as raw values (A) and means by group by study day (B) with the shaded areas representing 95% confident intervals of the means. The mean values (B) are also zoomed in to show the difference and trajectories two groups (C).

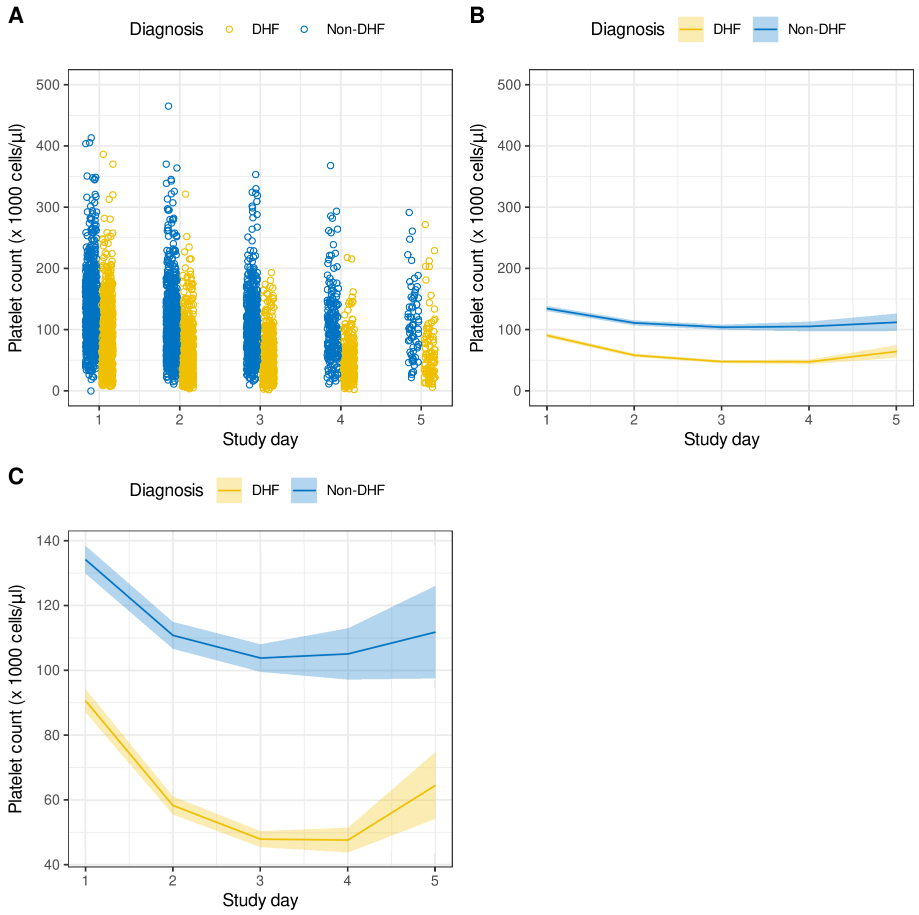

Fig. S1-20: Time course of `platelet count` variable from study day one to five in two groups (DHF and Non-DHF). The data are shown as raw values (A) and means by group by study day (B) with the shaded areas representing 95% confident intervals of the means. The mean values (B) are also zoomed in to show the difference and trajectories two groups (C).

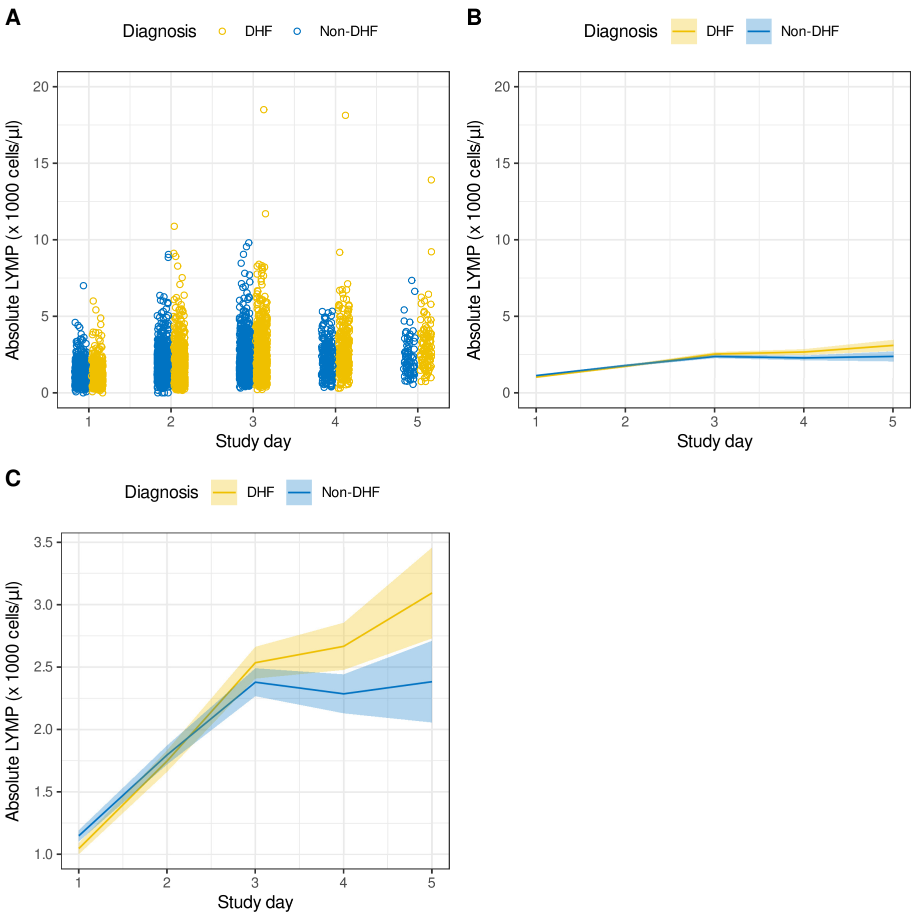

Fig. S1-21: Time course of `Lymphocyte` variable from study day one to five in two groups (DHF and Non-DHF). The data are shown as raw values (A) and means by group by study day (B) with the shaded areas representing 95% confident intervals of the means. The mean values (B) are also zoomed in to show the difference and trajectories two groups (C).

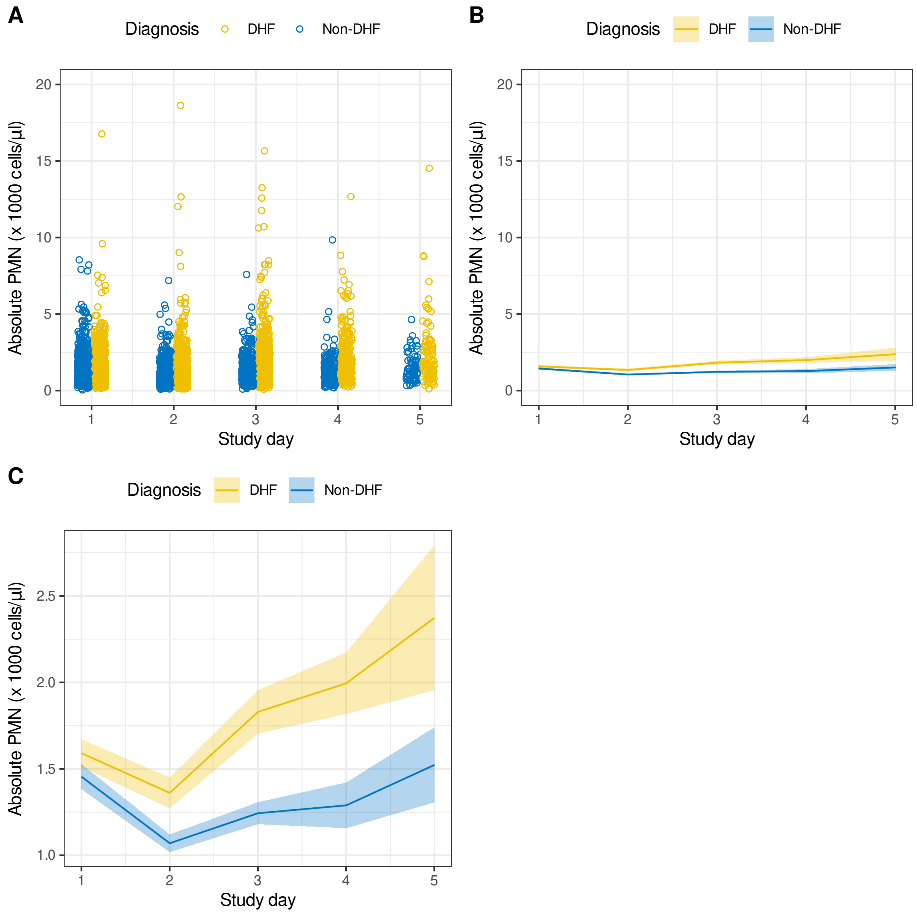

Fig. S1-22: Time course of `PMN` variable from study day one to five in two groups (DHF and Non-DHF). The data are shown as raw values (A) and means by group by study day (B) with the shaded areas representing 95% confident intervals of the means. The mean values (B) are also zoomed in to show the difference and trajectories two groups (C).

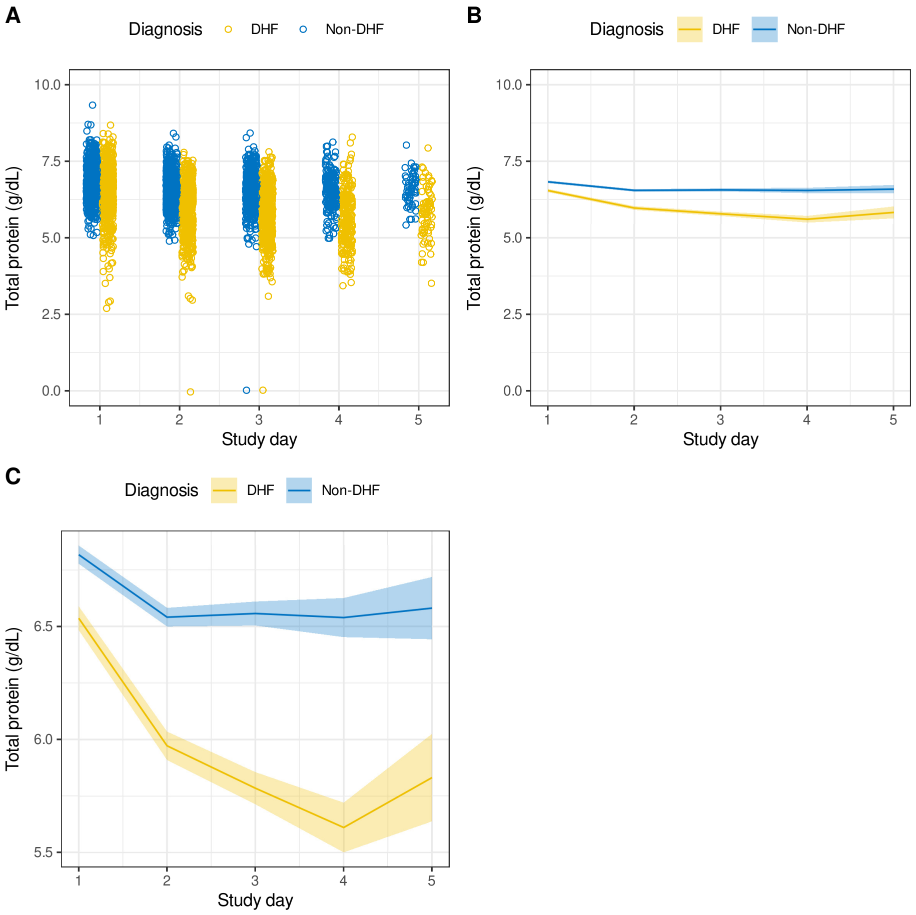

Fig. S1-23: Time course of `protein` variable from study day one to five in two groups (DHF and Non-DHF). The data are shown as raw values (A) and means by group by study day (B) with the shaded areas representing 95% confident intervals of the means. The mean values (B) are also zoomed in to show the difference and trajectories two groups (C).

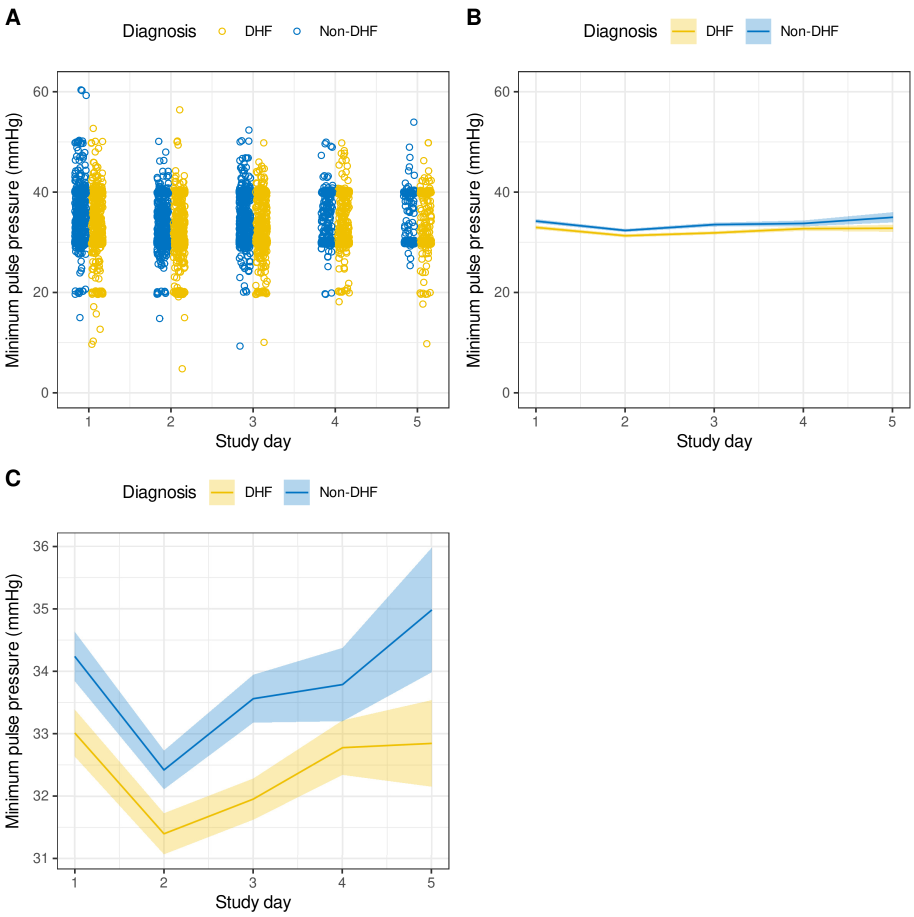

Fig. S1-24: Time course of `Average daily pulse pressure` variable from study day one to five in two groups (DHF and Non-DHF). The data are shown as raw values (A) and means by group by study day (B) with the shaded areas representing 95% confident intervals of the means. The mean values (B) are also zoomed in to show the difference and trajectories two groups (C).

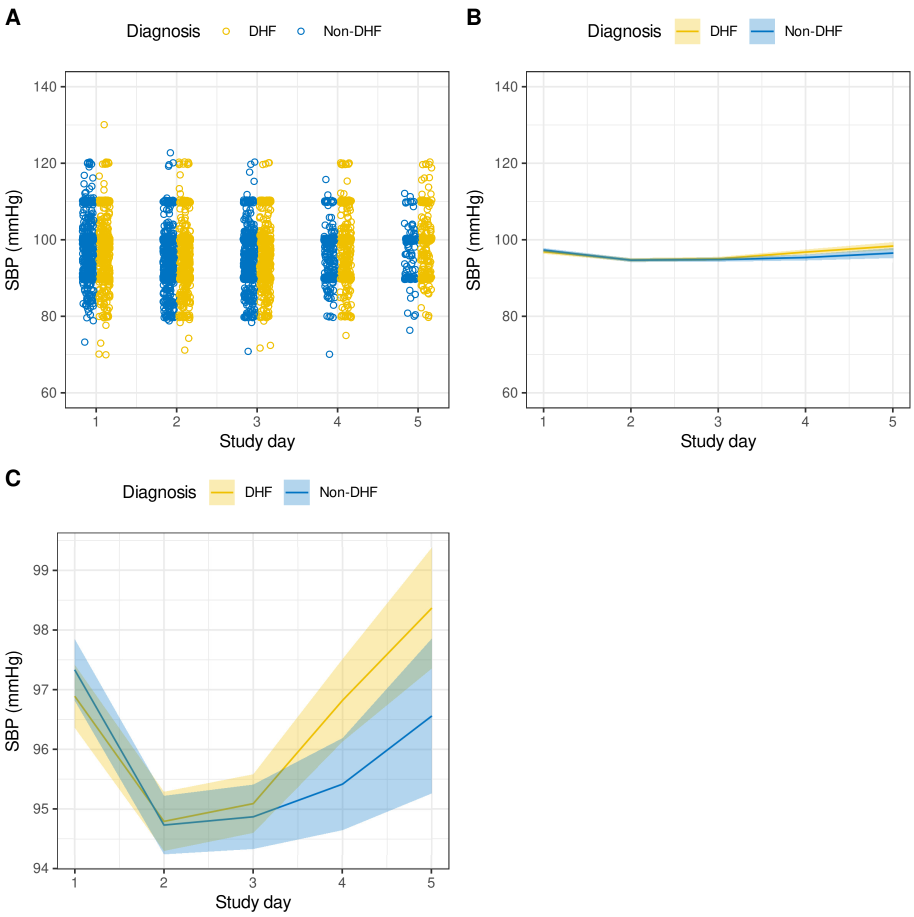

Fig. S1-25: Time course of `Minimum daily blood Pressure (Systolic)` variable from study day one to five in two groups (DHF and Non-DHF). The data are shown as raw values (A) and means by group by study day (B) with the shaded areas representing 95% confident intervals of the means. The mean values (B) are also zoomed in to show the difference and trajectories two groups (C).

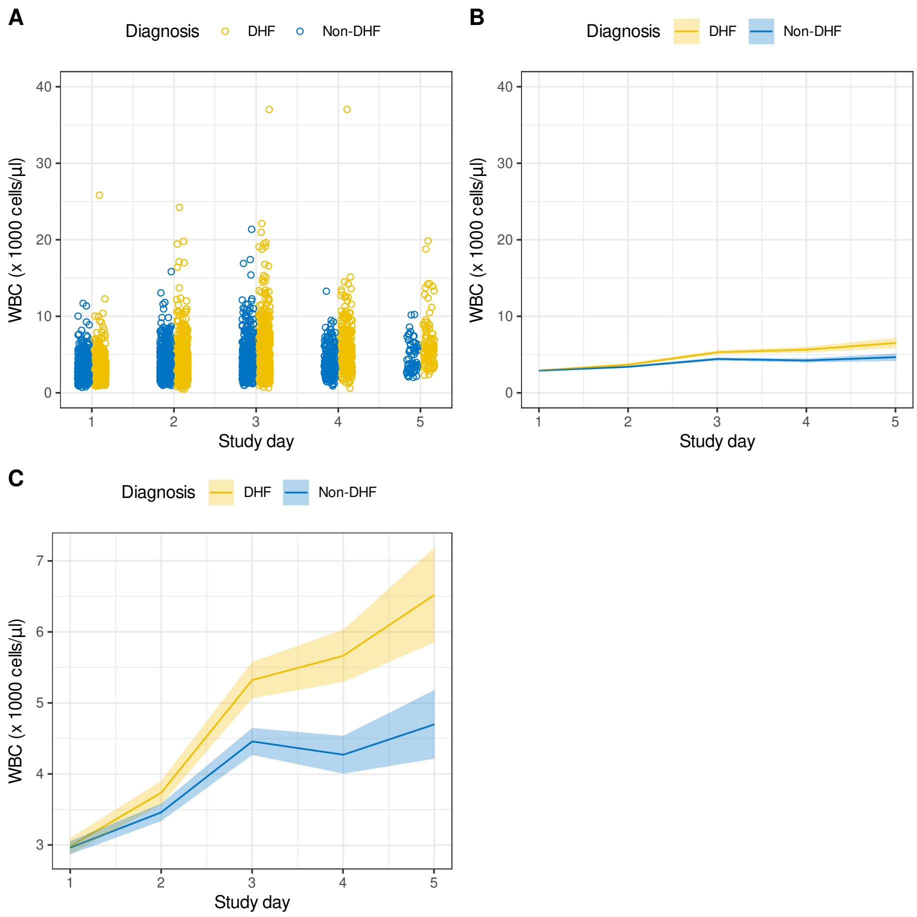

Fig. S1-26: Time course of `Weight Blood Cell` variable from study day one to five in two groups (DHF and Non-DHF). The data are shown as raw values (A) and means by group by study day (B) with the shaded areas representing 95% confident intervals of the means. The mean values (B) are also zoomed in to show the difference and trajectories two groups (C).

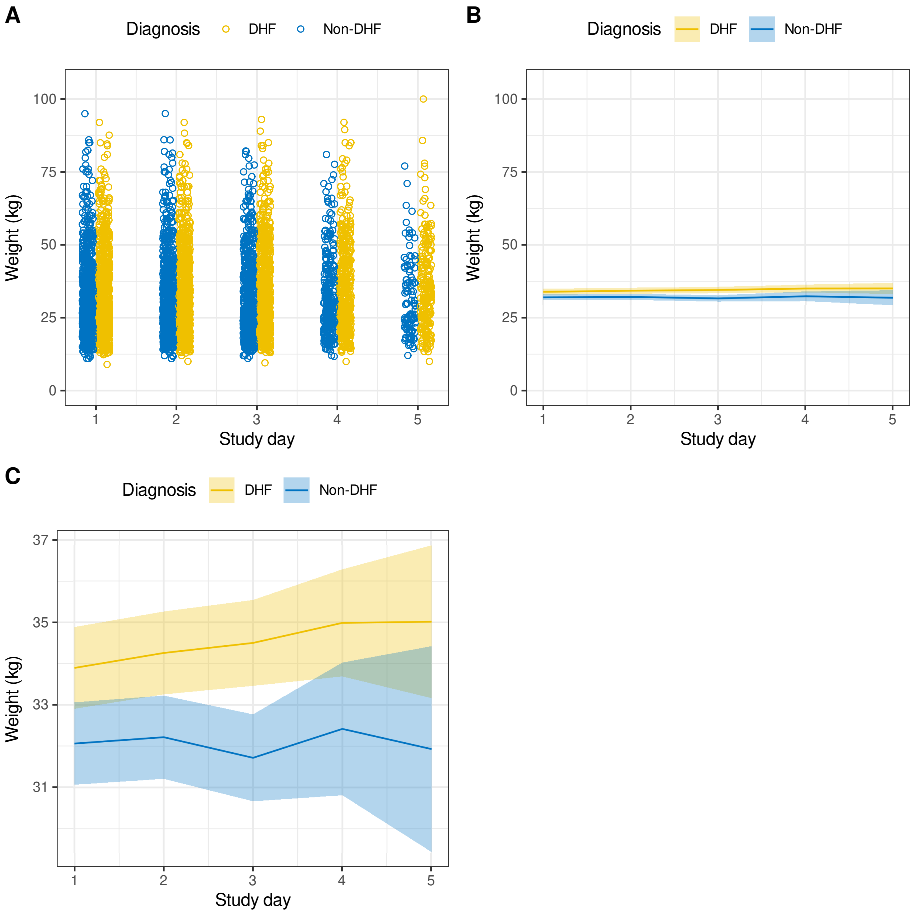

Fig. S1-27: Time course of `Weight` variable from study day one to five in two groups (DHF and Non-DHF). The data are shown as raw values (A) and means by group by study day (B) with the shaded areas representing 95% confident intervals of the means. The mean values (B) are also zoomed in to show the difference and trajectories two groups (C).
