# Supplementary material for: Prognostic prediction of dengue hemorrhagic fever in pediatric patients with suspected dengue infection: A multi-site study
Source: PLoS One. 2025 Aug 4;20(8):e0327360. doi: 10.1371/journal.pone.0327360 (PMC12321061; doi:10.1371/journal.pone.0327360)
Supplement: S2 File — (PDF) [file pone.0327360.s002.pdf]

Supplement file 2

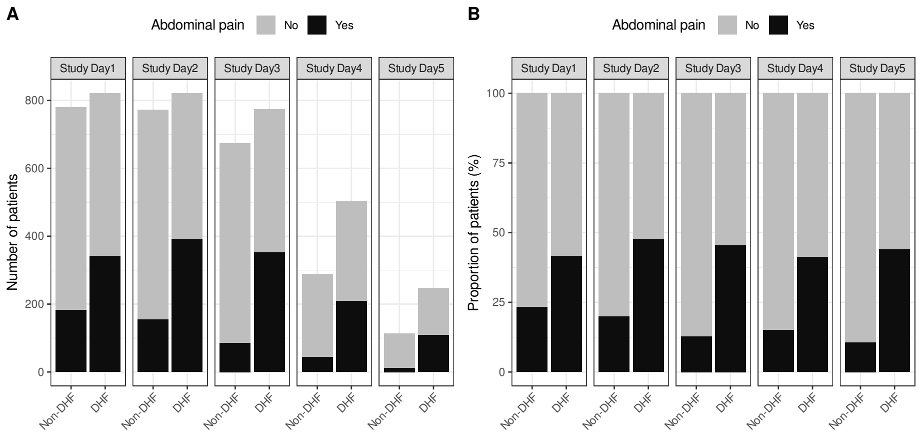

Fig. S2-1: Time course of `Abdominal pain` variable from study day one to five in two groups (DHF and Non-DHF). The data are shown as raw counts (A) and proportions by group by study day (B).

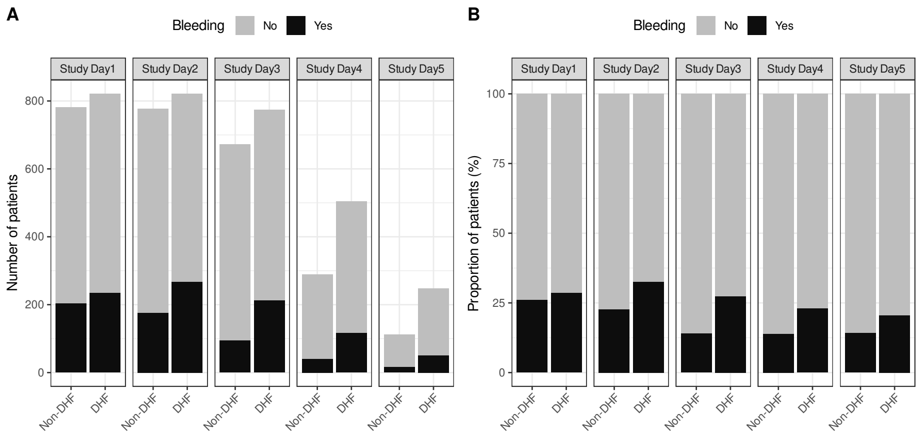

Fig. S2-2: Time course of `Bleeding` variable from study day one to five in two groups (DHF and Non-DHF). The data are shown as raw counts (A) and proportions by group by study day (B).

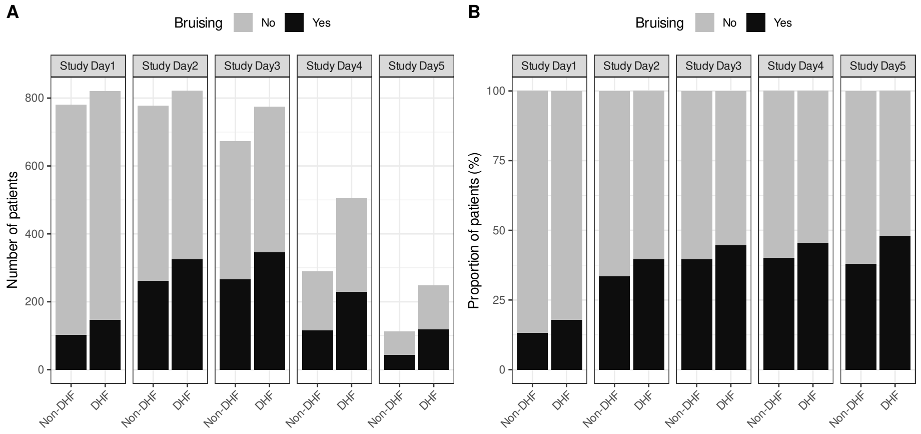

Fig. S2-3: Time course of `Bruising with venipuncture` variable from study day one to five in two groups (DHF and Non-DHF). The data are shown as raw counts (A) and proportions by group by study day (B).

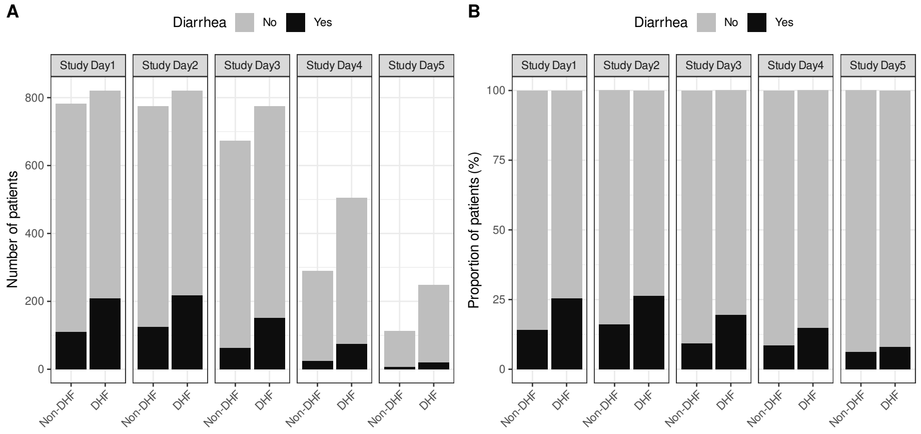

Fig. S2-4: Time course of `Diarrhea` variable from study day one to five in two groups (DHF and Non-DHF). The data are shown as raw counts (A) and proportions by group by study day (B).

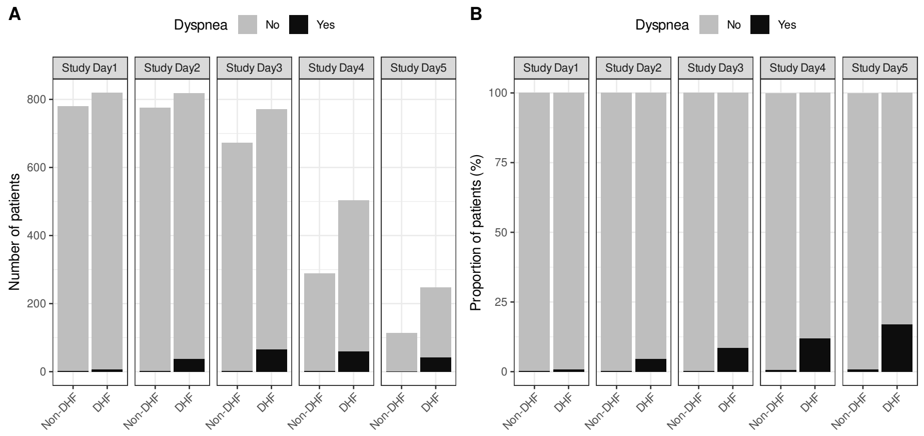

Fig. S2-5: Time course of `Dyspnea` variable from study day one to five in two groups (DHF and Non-DHF). The data are shown as raw counts (A) and proportions by group by study day (B).

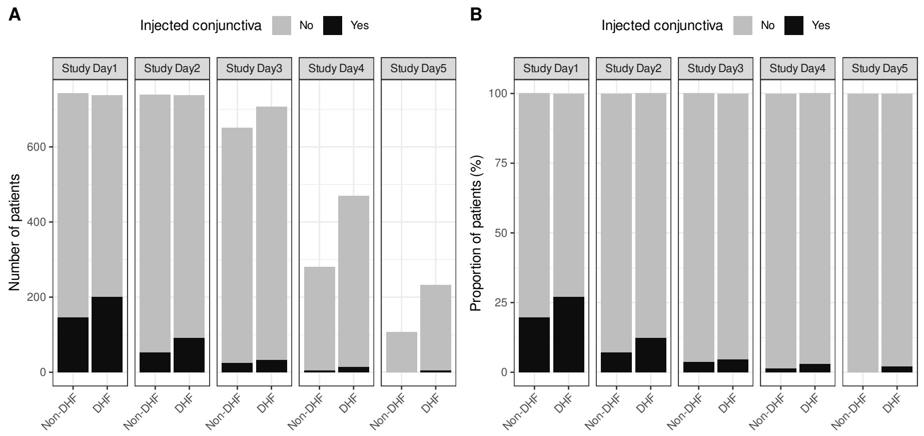

Fig. S2-6: Time course of `Injected conjunctivae` variable from study day one to five in two groups (DHF and Non-DHF). The data are shown as raw counts (A) and proportions by group by study day (B).

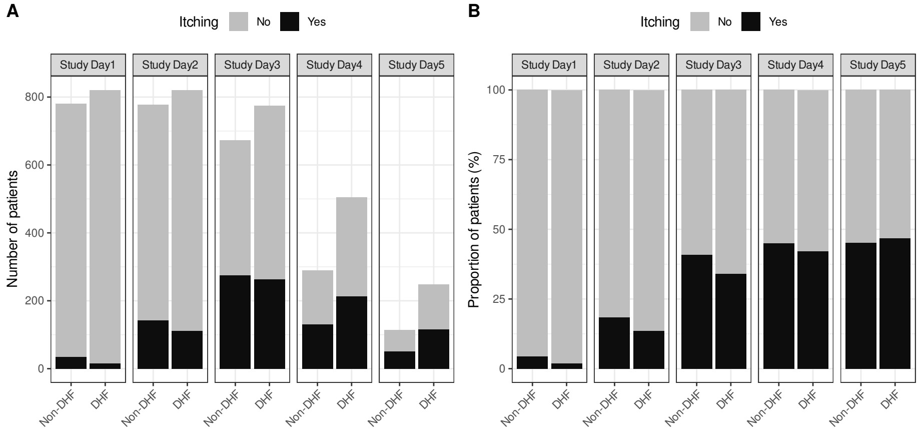

Fig. S2-7: Time course of `Itching related to rash` variable from study day one to five in two groups (DHF and Non-DHF). The data are shown as raw counts (A) and proportions by group by study day (B).

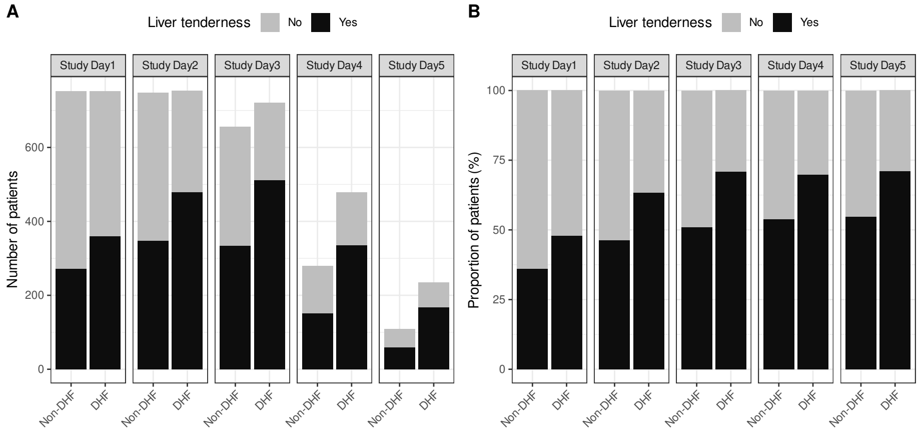

Fig. S2-8: Time course of `Liver tenderness` variable from study day one to five in two groups (DHF and Non-DHF). The data are shown as raw counts (A) and proportions by group by study day (B).

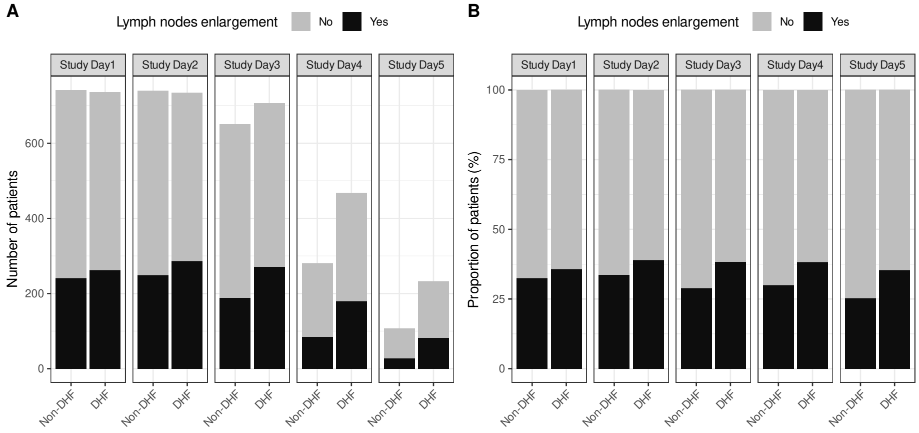

Fig. S2-9: Time course of `Lymph node enlargement` variable from study day one to five in two groups (DHF and Non-DHF). The data are shown as raw counts (A) and proportions by group by study day (B).

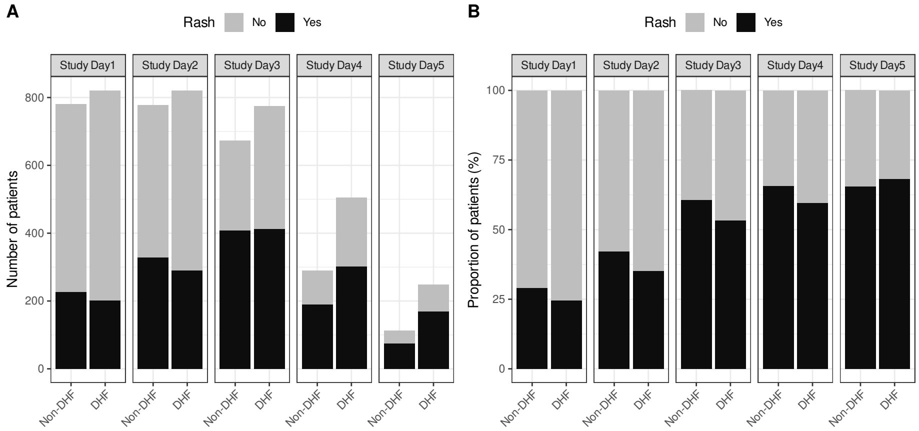

Fig. S2-10: Time course of `Rash` variable from study day one to five in two groups (DHF and Non-DHF). The data are shown as raw counts (A) and proportions by group by study day (B).

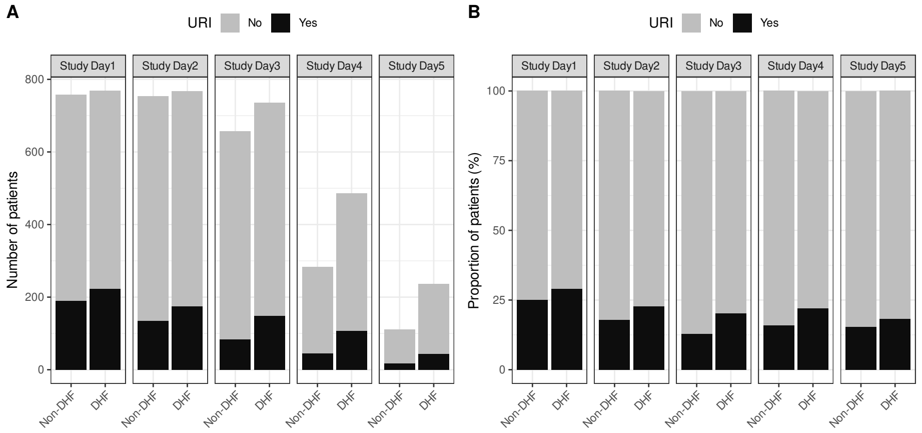

Fig. S2-11: Time course of `URI` variable from study day one to five in two groups (DHF and Non-DHF). The data are shown as raw counts (A) and proportions by group by study day (B).
