# Supplementary material for: Prognostic prediction of dengue hemorrhagic fever in pediatric patients with suspected dengue infection: A multi-site study
Source: PLoS One. 2025 Aug 4;20(8):e0327360. doi: 10.1371/journal.pone.0327360 (PMC12321061; doi:10.1371/journal.pone.0327360)
Supplement: S3 File — (PDF) [file pone.0327360.s003.pdf]

Supplement file 3

Table S3-1: Odds ratio

Interval	Odds ratio (95% CI)	p-value
**Age**		
(-inf, 10.5]		
(10.5, 12.5]	1.821 (0.327-15.013)	0.523
(12.5, 13.5]	2.843 (0.503-23.667)	0.270
(13.5, 14.5]	2.097 (0.367-17.592)	0.436
(14.5, inf)	2.861 (0.474-24.884)	0.281
(2.5, 3.5]	1.707 (0.258-15.881)	0.600
(3.5, 6.5]	2.544 (0.476-20.422)	0.311
(6.5, 7.5]	1.156 (0.209-9.475)	0.877
(7.5, 10.5]	1.523 (0.235-9.817)	0.647
**Sex**		
Male		
Female	0.773 (0.656-0.911)	0.002
**Height**		
(-inf, 116.0]		
(116.0, 122.0]	n/a (n/a, n/a)	n/a
(122.0, 130.0]	1.482 (0.12-22.302)	0.763
(130.0, 138.5]	1.157 (0.091-17.826)	0.912
(138.5, 150.0]	n/a (n/a, n/a)	n/a
(150.0, 155.5]	n/a (n/a, n/a)	n/a
(155.5, 161.0]	n/a (n/a, n/a)	n/a
(161.0, inf]	n/a (n/a, n/a)	n/a
**Minimum daily blood pressure (Diastolic)**		
(-inf, 40.5]		
(40.5, 57.5]	0.455 (0.157-1.318)	0.144
(57.5, 58.5]	0.297 (0.09-0.975)	0.045
(58.5, 60.5]	0.464 (0.16-1.341)	0.154
(60.5, 62.5]	0.416 (0.134-1.292)	0.128
(62.5, 66.5]	0.682 (0.223-2.087)	0.500
(66.5, 68.5]	0.236 (0.063-0.867)	0.030
(68.5, 80.5]	0.5 (0.17-1.469)	0.205
(80.5, inf)	0.241 (0.033-1.74)	0.157
**Average fingerstick hematocrit**		
(-inf, 27.75]		
(27.75, 31.25]	0.25 (0.009-10.13)	0.419
(31.25, 36.981]	0.283 (0.008-13.001)	0.483
(36.981, 38.308]	0.287 (0.008-13.472)	0.491
(38.308, 40.031]	0.349 (0.01-16.787)	0.566

continued ...

Table S3-1: Odds ratio

Interval	Odds ratio (95% CI)	p-value
(40.031, 41.25]	0.489 (0.013-24.356)	0.699
(41.25, 44.25]	0.543 (0.014-28.132)	0.745
(44.25, 48.75]	0.751 (0.017-42.355)	0.882
(48.75, inf)	2.115 (0.029-195.351)	0.734
**Maximum fingerstick hematocrit**		
(-inf, 32.5]		
(32.5, 37.865]	1.349 (0.493-3.9)	0.568
(37.865, 40.253]	1.326 (0.448-4.13)	0.617
(40.253, 41.886]	2.334 (0.712-8.016)	0.169
(41.886, 42.045]	1.335 (0.385-4.827)	0.653
(42.045, 44.5]	1.674 (0.443-6.578)	0.453
(44.5, 46.5]	2.086 (0.487-9.245)	0.327
(46.5, 50.5]	2.135 (0.406-11.578)	0.374
(50.5, inf)	0.995 (0.063-31.962)	0.997
**Minimum fingerstick hematocrit**		
(-inf, 25.5]		
(25.5, 30.75]	3.168 (0.088-67.657)	0.473
(30.75, 35.283]	2.941 (0.075-69.676)	0.515
(35.283, 36.434]	3.294 (0.082-81.797)	0.479
(36.434, 38.016]	2.346 (0.056-60.906)	0.617
(38.016, 40.1]	2.234 (0.051-61.676)	0.643
(40.1, 41.5]	1.834 (0.04-53.84)	0.731
(41.5, 44.5]	1.969 (0.042-60.259)	0.705
(44.5, inf)	1.465 (0.028-51.83)	0.837
**Fingerstick hematocrit range**		
(-inf, 0.9]		
(0.9, 1.947]	0.832 (0.619-1.115)	0.219
(1.947, 2.257]	0.885 (0.663-1.18)	0.406
(2.257, 3.564]	1.099 (0.786-1.537)	0.580
(3.564, 4.029]	1.279 (0.864-1.893)	0.219
(4.029, 5.5]	1.346 (0.836-2.17)	0.222
(5.5, 6.75]	1.823 (0.977-3.406)	0.059
(6.75, 8.5]	3.062 (1.544-6.098)	0.001
(8.5, inf)	5.749 (1.895-18.455)	0.003
**Daily max difference between fluid intake and output**		
(-inf, 5.0]	0.378 (0.136-0.97)	0.051
(1130.0, 1540.0]	1.362 (0.749-2.523)	0.318
(147.5, 287.5]	0.7 (0.525-0.931)	0.015
(1540.0, inf)	5.117 (1.1-37.63)	0.058
(287.5, 545.0]	0.794 (0.627-1.004)	0.054
(5.0, 147.5]	0.43 (0.296-0.619)	0.000

continued ...

Table S3-1: Odds ratio

Interval	Odds ratio (95% CI)	p-value
(545.0, 845.0]	0.906 (0.691-1.188)	0.476
(845.0, 1130.0]	0.812 (0.542-1.215)	0.310
**Minimum daily pulse pressure**		
(-inf, 16.5]		
(16.5, 20.5]	5.909 (0.753-54.681)	0.097
(20.5, 24.5]	4.304 (0.414-51.595)	0.229
(24.5, 26.5]	7.009 (0.865-66.834)	0.073
(26.5, 27.5]	5.494 (0.634-55.127)	0.127
(27.5, 28.5]	7.26 (0.968-64.525)	0.058
(28.5, 34.5]	4.407 (0.67-34.947)	0.130
(34.5, 38.5]	3.824 (0.573-30.722)	0.174
(38.5, inf)	4.193 (0.634-33.379)	0.144
**Average daily pulse rate**		
(-inf, 76.5]		
(121.5, 126.5]	15.229 (1.884-158.768)	0.015
(126.5, inf)	2.738 (0.319-29.785)	0.379
(70.5, 73.5]	5.316 (0.608-58.722)	0.147
(73.5, 76.5]	2.569 (0.314-26.773)	0.399
(76.5, 79.5]	5.517 (0.736-53.931)	0.114
(79.5, 80.75]	4.124 (0.524-42.001)	0.199
(80.75, 98.5]	3.451 (0.495-31.896)	0.237
(98.5, 121.5]	4.754 (0.674-44.337)	0.139
**Average daily body temperature**		
(36.375, 36.425]	2.817 (0.523-17.374)	0.245
(36.425, 36.675]	0.837 (0.271-2.728)	0.762
(36.675, 36.925]	1.202 (0.366-4.144)	0.765
(36.925, 38.175]	1.051 (0.308-3.765)	0.938
(38.175, 38.725]	1.289 (0.358-4.848)	0.702
(38.725, 38.775]	0.659 (0.161-2.792)	0.566
(38.775, 38.925]	1.848 (0.477-7.452)	0.380
(38.925, inf)	1.222 (0.309-5.01)	0.778
**Maximum daily body temperature**		
(-inf, 37.15]		
(37.15, 38.35]	0.517 (0.264-1.014)	0.054
(38.35, 38.95]	0.522 (0.254-1.078)	0.078
(38.95, 39.05]	0.727 (0.332-1.599)	0.427
(39.05, 39.65]	0.708 (0.342-1.473)	0.353
(39.65, 40.65]	1.035 (0.481-2.24)	0.931
(40.65, 40.85]	0.519 (0.168-1.597)	0.253
(40.85, 40.95]	2.434 (0.532-11.94)	0.258
(40.95, inf)	2.574 (0.639-11.584)	0.198

continued . . .

Table S3-1: Odds ratio

Interval	Odds ratio (95% CI)	p-value
**Minimum daily body temperature**		
(-inf, 36.65]		
(36.65, 37.05]	0.891 (0.697-1.138)	0.355
(37.05, 37.15]	0.794 (0.509-1.228)	0.303
(37.15, 37.25]	1.012 (0.642-1.595)	0.961
(37.25, 37.55]	1.057 (0.751-1.486)	0.749
(37.55, 38.35]	1.272 (0.87-1.857)	0.214
(38.35, 39.45]	1.347 (0.779-2.331)	0.286
(39.45, 39.85]	0.553 (0.205-1.455)	0.234
(39.85, inf)	0.846 (0.296-2.45)	0.755
**Abdominal Circumference**		
(-inf, 44.75]		
(44.75, 46.25]	0.33 (0.167-0.655)	0.001
(46.25, 47.625]	0.743 (0.372-1.496)	0.402
(47.625, 48.25]	0.423 (0.21-0.856)	0.016
(48.25, 58.008]	0.451 (0.241-0.854)	0.014
(58.008, 59.091]	0.231 (0.11-0.487)	0.000
(59.091, 59.865]	10.078 (3.438-34.797)	0.000
(59.865, 63.25]	0.668 (0.334-1.349)	0.257
(63.25, inf)	0.33 (0.164-0.669)	0.002
**Abdominal Pain**		
No		
Yes	1.425 (1.159-1.752)	0.001
**Diarrhea**		
No		
Yes	1.565 (1.245-1.969)	0.000
**Itching related to rash**		
No		
Yes	0.757 (0.54-1.057)	0.104
**Limbus**		
No		
Yes	1.337 (0.863-2.078)	0.195
**Liver tenderness**		
No		
Yes	1.163 (0.908-1.49)	0.233
**Liver Size**		
(-inf, 0.25]		
(0.25, 0.8]	0.806 (0.592-1.094)	0.167
(0.8, 1.25]	1.145 (0.856-1.53)	0.361
(1.25, 1.75]	1.585 (0.868-2.932)	0.138

continued ...

Table S3-1: Odds ratio

Interval	Odds ratio (95% CI)	p-value
(1.75, 2.25]	1.303 (0.953-1.782)	0.097
(2.25, 3.25]	1.831 (1.152-2.918)	0.011
(3.25, 4.75]	3.73 (1.772-8.165)	0.001
(4.75, 5.5]	7.117 (1.563-43.566)	0.019
(5.5, inf)	0.517 (0.102-2.596)	0.418
**Maculopapular**		
No		
Yes	0.526 (0.347-0.791)	0.002
**Rash**		
No		
Yes	0.758 (0.603-0.952)	0.017
**Upper respiratory infection (Daily examination)**		
No		
Yes	0.898 (0.724-1.112)	0.325
**Weight**		
(-inf, 12.55]		
(12.55, 13.15]	7.301 (1.179-57.681)	0.044
(13.15, 13.95]	1.954 (0.349-12.814)	0.463
(13.95, 18.05]	1.216 (0.315-5.75)	0. 789
(18.05, 28.35]	2.118 (0.514-10.535)	0.324
(28.35, 28.55]	9.977 (1.298-95.576)	0.034
(28.55, 28.75]	29.832 (2.415-888.336)	0.018
(28.75, 34.95]	2.625 (0.617-13.404)	0.214
(34.95, inf)	2.556 (0.587-13.291)	0.233
**HCT (laboratoty)**		
(-inf, 24.9]		
(24.9, 28.25]	0.553 (0.06-8.479)	0.631
(28.25, 35.15]	0.895 (0.112-12.878)	0.925
(35.15, 37.15]	0.903 (0.112-13.09)	0.931
(37.15, 39.05]	0.818 (0.101-11.908)	0.866
(39.05, 40.55]	0.924 (0.113-13.576)	0.948
(40.55, 43.25]	1.029 (0.125-15.135)	0.981
(43.25, 43.55]	2.5 (0.137-106.253)	0.577
(43.55, inf)	0.94 (0.11-14.147)	0.959
**Albumin**		
(-inf, 2.05]		
(2.05, 2.15]	0.121 (0.006-1.984)	0.154
(2.15, 2.75]	0.423 (0.034-3.689)	0.461
(2.75, 2.85]	0.392 (0.03-3.66)	0.434
(2.85, 3.075]	0.371 (0.03-3.256)	0.397

continued . . .

Table S3-1: Odds ratio

Interval	Odds ratio (95% CI)	p-value
(3.075, 3.15]	0.197 (0.016-1.743)	0.167
(3.15, 3.25]	0.208 (0.017-1.828)	0.180
(3.25, 3.65]	0.16 (0.013-1.394)	0.117
(3.65, inf)	0.134 (0.011-1.172)	0.086
**AST**		
(-inf, 23.5]		
(139.5, 832.5]	0.549 (0.169-1.8)	0.320
(23.5, 31.5]	0.348 (0.128-0.954)	0.038
(31.5, 43.5]	0.478 (0.179-1.302)	0.144
(43.5, 50.5]	0.442 (0.156-1.271)	0.126
(50.5, 70.5]	0.448 (0.157-1.291)	0.133
(70.5, 99.5]	0.514 (0.172-1.55)	0.234
(832.5, inf)	0.686 (0.091-5.314)	0.716
(99.5, 139.5]	0.546 (0.176-1.709)	0.295
**ALT**		
(-inf, 8.5]		
(16.5, 18.5]	0.735 (0.233-2.465)	0.606
(18.5, 36.5]	1.165 (0.425-3.485)	0.774
(36.5, 38.5]	1.678 (0.568-5.343)	0.362
(362.0, 453.0]	8.184 (0.779-211.408)	0.117
(38.5, 45.5]	1.511 (0.529-4.668)	0.454
(45.5, 362.0]	1.498 (0.517-4.679)	0.469
(453.0, inf)	6.545 (1.068-48.057)	0.050
(8.5, 16.5]	0.608 (0.212-1.886)	0.369
**Protein**		
(-inf, 3.25]		
(3.25, 4.75]	17.02 (0.31-801.969)	0.175
(4.75, 4.95]	8.069 (0.17-295.714)	0.299
(4.95, 5.375]	2.94 (0.074-81.867)	0.573
(5.375, 5.55]	2.043 (0.05-59.306)	0.711
(5.55, 6.133]	1.858 (0.047-52.043)	0.746
(6.133, 6.293]	1.955 (0.049-55.613)	0.727
(6.293, 7.167]	1.765 (0.045-49.513)	0.767
(7.167, inf)	1.641 (0.042-46.334)	0.796
**Lymphocyte**		
(-inf, 2.779]		
(2.779, 2.931]	0.923 (0.703-1.212)	0.565
(2.931, 2.934]	6.862 (1.276-56.433)	0.040
(2.934, 2.96]	0.564 (0.359-0.881)	0.012
(2.96, 3.001]	0.803 (0.56-1.149)	0.230
(3.001, 3.038]	0.734 (0.5-1.074)	0.112

continued ...

Table S3-1: Odds ratio

Interval	Odds ratio (95% CI)	p-value
(3.038, 3.196]	0.596 (0.444-0.8)	0.001
(3.196, 3.211]	0.17 (0.073-0.37)	0.000
(3.211, inf)	0.573 (0.405-0.809)	0.002
**Platelet count**		
(-inf, 8.5]	0.848 (0.159-4.354)	0.844
(104.25, 129.75]	0.539 (0.1-2.804)	0.464
(129.75, 180.75]	0.343 (0.061-1.853)	0.216
(21.5, 40.5]	5.187 (1.055-24.51)	0.038
(40.5, 44.5]	3.312 (0.609-17.453)	0.159
(44.5, 69.5]	2.025 (0.4-9.872)	0.384
(69.5, 104.25]	1.112 (0.213-5.594)	0.898
(8.5, 21.5]	6.553 (1.204-35.338)	0.028
(180.75, inf)		
**AST/platelet ratio**		
(-inf, 0.12]		
(0.12, 0.156]	4.543 (1.516-16.09)	0.011
(0.156, 0.195]	2.512 (0.791-9.377)	0.139
(0.195, 0.441]	6.61 (2.181-24.098)	0.002
(0.441, 1.353]	8.304 (2.528-32.207)	0.001
(1.353, 3.096]	10.797 (3.006-44.951)	0.001
(15.075, inf)	17.14 (2.61-130.405)	0.004
(3.096, 5.192]	10.126 (2.621-44.729)	0.001
(5.192, 15.075]	9.693 (2.362-45.018)	0.002
**AST/ALT ratio**		
(-inf, 0.851]		
(0.851, 0.933]	1.694 (0.762-3.779)	0.196
(0.933, 0.974]	0.239 (0.06-0.797)	0.028
(0.974, 1.419]	1.944 (1.058-3.653)	0.035
(1.419, 1.449]	0.807 (0.325-1.975)	0.640
(1.449, 1.661]	2.282 (1.202-4.422)	0.013
(1.661, 1.718]	0.997 (0.464-2.161)	0.994
(1.718, 1.772]	4.208 (1.981-9.095)	0.000
(1.772, inf)	2.098 (1.111-4.045)	0.024
**WBC**		
(-inf, 2.929]		
(2.929, 3.06]	1.383 (0.16-8.525)	0.741
(3.06, 3.13]	1.891 (0.23-10.85)	0.502
(3.13, 3.19]	0.871 (0.107-4.939)	0.883
(3.19, 3.217]	2.072 (0.25-12.061)	0.445
(3.217, 3.337]	1.062 (0.135-5.728)	0.948
(3.337, 3.473]	1.484 (0.188-7.985)	0.668

continued ...

Table S3-1: Odds ratio

**Interval**	**Odds ratio (95% CI)**	**p-value**
(3.473, 3.744]	1.457 (0.185-7.852)	0.682
(3.744, inf)	1.486 (0.185-8.204)	0.671
**Day of fever**		
(-inf, 1.5]		
(1.5, 2.5]	0.82 (0.521-1.292)	0.392
(2.5, 3.5]	0.42 (0.264-0.668)	2.49E-04
(3.5, 4.5]	0.197 (0.119-0.323)	1.48E-10
(4.5, 5.5]	0.099 (0.057-0.169)	5.20E-17
(5.5, 6.5]	0.089 (0.049-0.162)	3.00E-15
(6.5, 7.5]	0.075 (0.032-0.172)	1.76E-09
(7.5, inf)	0.045 (0.011-0.147)	1.85E-06
