# Supplementary material for: Prognostic prediction of dengue hemorrhagic fever in pediatric patients with suspected dengue infection: A multi-site study
Source: PLoS One. 2025 Aug 4;20(8):e0327360. doi: 10.1371/journal.pone.0327360 (PMC12321061; doi:10.1371/journal.pone.0327360)
Supplement: S4 File — (PDF) [file pone.0327360.s004.pdf]

Supplement file 4

Table S4-1: Features in five categories (Combined dataset)

	General Hospital (GH)	Primary Care Unit (PCU)
**Demographics and baseline physical examination/interview**	Day of fever<br>Age<br>Sex	Age<br>Day of fever<br>Sex<br>Weight
**Daily vital sign**	Maximum fingerstick hematocrit<br>Average daily body temperature<br>fingerstick hematocrit range<br>Maximum daily body temperature<br>Minimum daily body temperature<br>Minimum fingerstick hematocrit	Average daily body temperature<br>Minimum daily pulse pressure<br>Average daily pulse pressure
**Daily physical examination/interview**	Abdominal Circumference<br>Daily max difference between fluid intake and output<br>Difference between fluid intake and output<br>Liver size<br>Abdominal pain	Abdominal Circumference<br>Liver size<br>Abdominal pain<br>Quantity Tourniquet test (on admission)<br>Quantity Tourniquet (Daily examination)<br>Injected conjunctivae
**Daily laboratory checkup measures**	AST/platelet ratio<br>Platelet count<br>Lymphocyte<br>Albumin<br>AST<br>Protein<br>ALT<br>AST/ALT ratio<br>PMN<br>HCT (laboratory)	
**Derived features (d, da, ds, d12, d23, d13)**	Platelet count<br>Average fingerstick hematocrit<br>Maximum fingerstick hematocrit<br>AST/platelet ratio<br>WBC<br>Daily max difference between fluid intake and output<br>HCT (laboratory)<br>AST/ALT ratio<br>Albumin<br>Protein	Abdominal Circumference<br>Weight<br>Liver size<br>Minimum daily pulse pressure<br>Average daily body temperature<br>Minimum daily blood pressure (Systolic)

Note Aspartate Transaminase (AST), Alanine Transaminase (ALT) are liver enzymes

Table S4-2: Features in five categories (SK dataset)

	General Hospital (GH)	Primary Care Unit (PCU)
**Demographics and baseline physical examination/interview**	Day of fever<br>Age<br>Weight<br>Sex	Age<br>Day of fever<br>Sex<br>Quantity Tourniquet (on admission)<br>Quality Tourniquet (on admission)<br>Height<br>Upper respiratory infection (on admission)
**Daily vital sign**	Average daily body temperature<br>Fingerstick hematocritrange<br>Maximum daily body temperature<br>Maximum fingerstick hematocrit<br>Minimum daily body temperature<br>Average fingerstick hematocrit<br>Range of daily Pulse rate<br>Minimum daily pulse pressure<br>Minimum daily blood pressure (Diastolic)	Average daily body temperature<br>Minimum daily pulse pressure<br>Minimum daily blood pressure (Diastolic)<br>Average daily pulse pressure<br>Minimum daily blood pressure (Systolic)
**Daily physical examination/interview**	Daily max difference between fluid intake and output<br>Liver size<br>Abdominal pain<br>Abdominal Circumference<br>Rash	Abdominal pain<br>Bleeding (Daily examination)<br>Rash<br>d04iarrhea<br>Liver size<br>Abdominal Circumference<br>Upper respiratory infection (Daily examination)<br>Bruising with venipuncture<br>Liver tenderness<br>Quantity Tourniquet (Daily examination)<br>Confluent petechial<br>Maculopapular
**Daily laboratory checkup measures**	AST/platelet ratio<br>Platelet count<br>Lymphocyte<br>Albumin<br>AST<br>Protein<br>HCT (laboratory)<br>ALT<br>Monocyte<br>AST/ALT ratio<br>Band<br>Eosin	
**Derived features (d, da, ds, d12, d23, d13)**	Platelet count<br>AST/ALT ratio<br>Maximum fingerstick hematocrit<br>AST/platelet ratio<br>Average fingerstick hematocrit<br>WBC<br>Albumin<br>Difference between fluid intake and output<br>Protein<br>HCT (laboratory)	Liver size<br>Abdominal Circumference<br>Minimum daily blood pressure (Systolic)<br>Minimum daily pulse pressure<br>Average daily body temperature<br>Weight

Note Aspartate Transaminase (AST), Alanine Transaminase (ALT) are liver enzymes

Table S4-3: Features in five categories (KK dataset)

	General Hospital (GH)	Primary Care Unit (PCU)
**Demographics and baseline physical examination/interview**	Day of fever<br>Age<br>Sex	Age<br>Upper respiratory infection (on admission)<br>Quantity Tourniquet (on admission)<br>Sex<br>Day of fever<br>Bleeding (on admission)<br>Weight<br>Quality Tourniquet (on admission)<br>Height<br>JE vaccine
**Daily vital sign**	Fingerstick hematocrit range<br>Maximum fingerstick hematocrit<br>Average daily body temperature<br>Minimum daily body temperature<br>Maximum daily body temperature<br>Minimum fingerstick hematocrit	Average daily body temperature<br>Average daily pulse pressure<br>Minimum daily pulse pressure<br>Minimum daily blood pressure (Diastolic)<br>Minimum daily blood pressure (Systolic)
**Daily physical examination/interview**	Abdominal Circumference<br>Upper respiratory infection (Daily examination)<br>Difference between fluid intake and output<br>Liver size	Abdominal Circumference<br>Liver size<br>Liver tenderness<br>Lymph node enlargement<br>Quantity Tourniquet (Daily examination)<br>Abdominal pain<br>Upper respiratory infection (Daily examination)<br>Diarrhea<br>Non-productive cough<br>Limbus<br>Itching related to rash<br>Bleeding (Daily examination)<br>Bruising with venipuncture
**Daily laboratory checkup measures**	Platelet count<br>AST/platelet ratio<br>Albumin<br>Lymphocyte<br>AST/ALT ratio<br>Protein<br>ALT<br>AST<br>WBC<br>HCT (laboratory)<br>Monocyte<br>PMN	
**Derived features (d, da, ds, d12, d23, d13)**	Platelet count<br>Average fingerstick hematocrit<br>Liver size<br>Maximum fingerstick hematocrit<br>AST/platelet ratio<br>HCT (laboratory)<br>WBC<br>Maximum daily body temperature<br>ALT<br>AST	Abdominal Circumference<br>Liver size<br>Average daily body temperature<br>Average daily pulse pressure

Note Aspartate Transaminase (AST), Alanine Transaminase (ALT) are liver enzymes
