# Supplementary material for: Prognostic prediction of dengue hemorrhagic fever in pediatric patients with suspected dengue infection: A multi-site study
Source: PLoS One. 2025 Aug 4;20(8):e0327360. doi: 10.1371/journal.pone.0327360 (PMC12321061; doi:10.1371/journal.pone.0327360)
Supplement: S5 File — (PDF) [file pone.0327360.s005.pdf]

Supplement file 5

Table S5-1: Selected features with feature importance scores for the 2-Day(GH) models trained with the two site-specific datasets.

	KK-Hospital		SK-Hospital	
	Feature	Score	Feature	Score
1	Platelet count (Day2)	2204.05	Platelet count (Day2)	2186.63
2	Average daily body temperature (Day2)	799.11	Platelet count(diff. between Day 1-2)	1034.42
3	AST/platelet ratio (Day2)	614.25	Average daily body temperature (Day2)	866.12
4	Fingerstick hematocrit range (Day2)	513.78	Fingerstick hematocrit range (Day2)	612.71
5	Maximum fingerstick hematocrit (Day2)	487.67	Day of fever	511.37
6	AST (Day1)	449.95	Difference between fluid intake and output (Day2)	474.39
7	Lymphocyte (Day2)	437.44	Maximum daily body temperature (Day2)	388.91
8	Fingerstick hematocrit range (Day1)	430.34	Maximum fingerstick hematocrit (Day2)	376.7
9	Albumin (Day2)	393.23	Age	328.13
10	ALT (Day1)	391.24	Lymphocyte (Day2)	325.56
11	Protein (Day2)	389.22	Albumin (Day2)	320.41
12	Age	343.61	Monocyte (Day2)	304.41
13	Platelet count (diff. between Day 1-2)	327.52	AST/ALT ratio (diff. between Day 1-2)	290.37
14	WBC (Day2)	305.8	Maximum fingerstick hematocrit (diff. between Day 1-2)	287.32
15	Average fingerstick hematocrit (diff. between Day 1-2)	269.67	AST/platelet ratio (diff. between Day 1-2)	275.72
16	Day of fever	267.84	Abdominal pain (Day2)	252.55
17	Difference between fluid intake and output (Day2)	255.61	Protein (Day2)	252.01
18	Minimum daily body temperature (Day2)	231.35	AST (Day2)	239.37
19	Liver size (diff. between Day 1-2)	222.96	Lymphocyte (Day1)	228.65
20	HCT (laboratory) (Day1)	206.99	Daily max difference between fluid intake and output (Day2)	224.37
21	Maximum fingerstick hematocrit (diff. between Day 1-2)	189.41	ALT (Day1)	224.28
22	Sex	185.48	AST/platelet ratio (Day1)	210.95
23	Platelet count (Day1)	167.2	Minimum daily pulse pressure (Day2)	183.43
24			Protein (Day1)	179.42
25			Minimum daily body temperature (Day2)	164.59
26			AST (Day1)	160.1
27			Average fingerstick hematocrit (diff. between Day 1-2)	145.3
28			Eosinophil (Day1)	120.32

Note: Aspartate Transaminase (AST), Alanine Transaminase (ALT) are liver enzymes
* Variables are in accordant with WHO Dengue fever diagnosis guidelines

Table S5-2: Selected features with feature importance scores for the 2-Day(PCU) models trained with the two site-specific datasets.

	KK-Hospital		SK-Hospital	
	Feature	Score	Feature	Score
1	Abdominal Circumference (Day1)	836.45	Abdominal pain (Day2)	823.11
2	Average daily body temperature (Day2)	642.57	Average daily body temperature (Day1)	733.32
3	Abdominal Circumference (Day2)	627.61	Age	655.36
4	Age	556.01	Liver size (Day2)	654.27
5	Abdominal Circumference (diff. between Day 1-2)	520.46	Average daily body temperature (Day2)	586.21
6	Liver size (Day2)	503.44	Weight (diff. between Day 1-2)	515.06
7	Average daily body temperature (Day1)	449.47	Average daily body temperature (diff. between Day 1-2)	463.28
8	Liver tenderness (Day2)	355.84	Minimum daily pulse pressure (Day1)	431.41
9	Weight (Day1)	342.18	Minimum daily pulse pressure (diff. between Day 1-2)	422.07
10	Quantity Data of Tourniquet test (On admission)	331.82	Weight (Day1)	397.05
11	Sex	296.14	Day of fever	395.19
12	Average daily pulse pressure (Day2)	292.07	Abdominal Circumference (diff. between Day 1-2)	384.77
13	Day of fever	280.13	Minimum daily blood pressure (Diastolic) (Day2)	351.66
14	Liver size (diff. between Day 1-2)	270.71	Abdominal Circumference (Day1)	324.12
15	Upper respiratory infection (On admission)	266.03	Average daily pulse pressure (Day2)	317.67
16	Average daily body temperature (diff. between Day 1-2)	262.89	Liver size (diff. between Day 1-2)	307.85
17	Abdominal pain (Day2)	256.22	Minimum daily blood pressure (Systolic) (Day2)	243.81
18	Quantity Tourniquet (Daily Examination) (Day2)	216.77	Abdominal Circumference (Day2)	240.78
19	Average daily pulse pressure (diff. between Day 1-2)	202.45	Sex	230.03
20	JE vaccine	190.47	Minimum daily blood pressure (Systolic) (Day1)	219.33
21	Minimum daily blood pressure (Systolic) (Day2)	186.77	Quantity Data of Tourniquet test (on admission)	178.75
22	Minimum daily blood pressure (Diastolic) (Day1)	181.14	Diarrhea (Day2)	169.59
23	Upper respiratory infection (Daily examination) (Day2)	159.13		
24	Lymph node enlargement (Day2)	143.93		
25	Bleeding (on admission)	132.68		
26	Average daily pulse pressure (Day1)	113.76		
27	Bruising with venipuncture (Day2)	107.7		
28	Minimum daily pulse pressure (Day1)	105.63		
29	Lymph node enlargement (Day1)	92.7		
30	Upper respiratory infection (Daily examination) (Day1)	91.84		

Note: Aspartate Transaminase (AST), Alanine Transaminase (ALT) are liver enzymes
* Variables are in accordant with WHO Dengue fever diagnosis guidelines

Table S5-3: Selected features with feature importance scores for the 3-Day(GH) models trained with the two site-specific datasets.

	KK-Hospital		SK-Hospital	
	Feature	Score	Feature	Score
1	Platelet count (Day3)	878.19	Platelet count (Day3)	1206.66
2	AST/platelet ratio (Slope)	660.84	Platelet count (Slope)	774.94
3	Maximum fingerstick hematocrit (Day3)	395.36	AST/platelet ratio (Day3)	405.13
4	Average daily body temperature (Day3)	333.96	Maximum fingerstick hematocrit (Slope)	369.94
5	Average daily body temperature (Day2)	224.39	Maximum daily body temperature (Day3)	343.43
6	Lymphocyte (Day3)	214.58	WBC (Slope)	282.77
7	Albumin (Day3)	199.88	Difference between fluid intake and output (Day3)	273.89
8	Fingerstick hematocrit range (Day2)	199.49	Albumin (Slope)	227.55
9	Liver size (Day3)	194.63	Average daily body temperature (Day1)	219.49
10	HCT (laboratory) (Intercept)	178.38	Average daily body temperature (Day2)	210.87
11	Fingerstick hematocrit range (Day3)	173.49	Daily max difference between fluid intake and output (Day3)	207.85
12	WBC (Slope)	167.82	Average HCT (Slope)	202.03
13	AST/platelet ratio (Day3)	159.53	Protein (Day3)	201.06
14	Monocyte (Day3)	153.76	Difference between fluid intake and output (Slope)	192.11
15	Maximum fingerstick hematocrit (diff. between Day 2-3)	142.59	Maximum fingerstick hematocrit (Day3)	188.82
16	Platelet count (Slope)	141.39	Minimum daily body temperature (Day3)	180.21
17	AST/platelet ratio (diff. between Day 2-3)	128.7	Abdominal pain (Day3)	163.17
18	Protein (Day3)	112.13	AST/ALT ratio (diff. between Day 2-3)	161.09
19	Maximum daily body temperature (Intercept)	111.85	ALT (Day2)	150.35
20	Sex	108.1	Difference between fluid intake and output (diff. between Day 2-3)	140.37
21	ALT (Day1)	106.43	Protein (Slope)	138.69
22	Platelet count (Day2)	105.6	HCT (laboratory) (Slope)	131.48
23	AST (Day1)	98.35		
24	Difference between fluid intake and output (Day3)	96		
25	Protein (Day1)	84.2		
26	ALT (Intercept)	80.71		
27	PMN (Day1)	64.24		
28	Liver size (Slope)	55.08		
29	AST (diff. between Day 1-2)	17.8		

Note: Aspartate Transaminase (AST), Alanine Transaminase (ALT) are liver enzymes
* Variables are in accordant with WHO Dengue fever diagnosis guidelines

Table S5-4: Selected features with feature importance scores for the 3-Day(PCU) models trained with the two site-specific datasets.

	KK-Hospital		SK-Hospital	
	Feature	Score	Feature	Score
1	Liver size (Day3)	619.47	Abdominal pain (Day3)	634.27
2	Abdominal Circumference (Day1)	581.76	Average daily body temperature (Day2)	557.59
3	Average daily body temperature (Day2)	533.78	Liver size (Slope)	405.54
4	Average daily body temperature (Day3)	327.7	Age	376.04
5	Abdominal pain (Day3)	306.22	Abdominal Circumference (Slope)	300.44
6			Average daily body temperature (Day1)	289.06
7			Abdominal Circumference (Intercept)	288.5
8			Minimum daily blood pressure (Systolic) (Slope)	274.14
9			Weight (Slope)	270.23
10			Minimum daily pulse pressure (Day2)	269.47
11			Minimum daily pulse pressure (Slope)	262.11
12			Day of fever	256.5
13			Weight (diff. between Day 2-3)	238.83
14			Average daily body temperature (Intercept)	210.58
15			Average daily body temperature (diff. between Day 2-3)	191.9
16			Bleeding (Daily examination) (Day3)	186.25
17			Minimum daily blood pressure (Diastolic) (Day1)	185.73
18			Liver size (diff. between Day 2-3)	175.51
19			Sex	148.76
20			Quantity Data of Tourniquet test (On admission)	145.33
21			Rash (Day3)	137.21
22			Diarrhea (Day3)	131.38

Note: Aspartate Transaminase (AST), Alanine Transaminase (ALT) are liver enzymes
* Variables are in accordant with WHO Dengue fever diagnosis guidelines
