# Supplementary material for: Prognostic prediction of dengue hemorrhagic fever in pediatric patients with suspected dengue infection: A multi-site study
Source: PLoS One. 2025 Aug 4;20(8):e0327360. doi: 10.1371/journal.pone.0327360 (PMC12321061; doi:10.1371/journal.pone.0327360)
Supplement: S6 File — (PDF) [file pone.0327360.s006.pdf]

Supplement file 6

Table S6-1: Core features for all models in combine site

	1-Day	2-Day	3-Day
**General Hospital (GH)**			
1	AST	Platelet count	Platelet count
2	Platelet count	AST	AST
3	Day of fever	Daily body temperature	Fingerstick hematocrit
4	Fingerstick hematocrit	Fingerstick hematocrit	Fluid intake and output
5	Lymphocyte	Day of fever	Liver size
6	Abdominal Circumference	Albumin	WBC
7	Daily body temperature	Lymphocyte	Daily body temperature
8	Albumin	Liver size	Albumin
9	Fluid intake and output	Fluid intake and output	Abdominal Circumference
10	Protein	HCT (laboratory)	HCT (laboratory)
11	Age	PMN	ALT
12	ALT	Age	Protein
13	Liver size	Protein	
14		Sex	
15		HCT (laboratory)	
16		Abdominal pain	
**Primary Care Unit (PCU)**			
1	Abdominal circumference	Liver size	Abdominal Pain
2	Daily body temperature	Daily body temperature	Liver size
3	Liver size	Abdominal pain	Age
4	Age	Abdominal circumference	Abdominal circumference
5	Abdominal pain	Age	Weight
6	Quantity of Tourniquet (on admission)	Weight	Pulse pressure
7	Day of fever	Day of fever	Daily body temperature
8	Sex	Pulse pressure	Day of fever
9	Weight	Quantity of Tourniquet (Daily examination)	Blood pressure (systolic)
10	Pulse pressure	Sex	Quantity of Tourniquet (Daily examination)
11			Quantity of Tourniquet (on admission)
12			Injected conjunctiva
13			Quality of Tourniquet (Daily examination)
