# Supplementary material for: Prognostic prediction of dengue hemorrhagic fever in pediatric patients with suspected dengue infection: A multi-site study
Source: PLoS One. 2025 Aug 4;20(8):e0327360. doi: 10.1371/journal.pone.0327360 (PMC12321061; doi:10.1371/journal.pone.0327360)
Supplement: S7 File — (PDF) [file pone.0327360.s007.pdf]

Supplement file 7

Non-DHF vs DHF Bootstrap

Table S7-1: Performance results of the models trained and tested on combined-site data for General Hospital (GH), Primary care unit (PCU) scenario. The columns Sensitivity and Specificity were both measured at the specified threshold of 0.5.

Model	Scenario	AUC (95% CI)	Sensitivity	Specificity
1-Day	GH	0.79 (0.76, 0.82)	0.69	0.73
	PCU	0.66 (0.62, 0.70)	0.53	0.71
2-Day	GH	0.87 (0.84, 0.89)	0.75	0.80
	PCU	0.76 (0.72, 0.79)	0.65	0.72
3-Day	GH	0.90 (0.87, 0.93)	0.78	0.84
	PCU	0.79 (0.75, 0.83)	0.68	0.75

Table S7-2: Performance results of the models trained with combined-site and tested on KK-hospital data for General Hospital (GH), Primary care unit (PCU) scenario. The columns Sensitivity and Specificity were both measured at the specified threshold of 0.5.

Model	Scenario	AUC (95% CI)	Sensitivity	Specificity
1-Day	GH	0.83 (0.78, 0.87)	0.73	0.75
	PCU	0.70 (0.65, 0.75)	0.59	0.71
2-Day	GH	0.86 (0.83, 0.90)	0.76	0.78
	PCU	0.77 (0.71, 0.82)	0.65	0.73
3-Day	GH	0.89 (0.84, 0.93)	0.76	0.82
	PCU	0.79 (0.73, 0.85)	0.68	0.75

Table S7-3: Performance results of the models trained with combined-site and tested on SK-hospital data for General Hospital (GH), Primary care unit (PCU) scenario. The columns Sensitivity and Specificity were both measured at the specified threshold of 0.5.

Model	Scenario	AUC (95% CI)	Sensitivity	Specificity
1-Day	GH	0.76 (0.70, 0.81)	0.66	0.71
	PCU	0.62 (0.56, 0.67)	0.47	0.70
2-Day	GH	0.87 (0.83, 0.90)	0.75	0.82
	PCU	0.75 (0.70, 0.80)	0.64	0.72
3-Day	GH	0.91 (0.87, 0.94)	0.79	0.86
	PCU	0.79 (0.73, 0.84)	0.67	0.76

Table S7-4: Performance results of the models trained on KK-hospital and tested on combined-sited data for General Hospital (GH), Primary care unit (PCU) scenario. The columns Sensitivity and Specificity were both measured at the specified threshold of 0.5.

Model	Scenario	AUC (95% CI)	Sensitivity	Specificity
1-Day	GH	0.78 (0.75, 0.82)	0.68	0.73
	PCU	0.65 (0.60, 0.68)	0.54	0.65
2-Day	GH	0.84 (0.81, 0.87)	0.76	0.75
	PCU	0.72 (0.67, 0.76)	0.70	0.59
3-Day	GH	0.88 (0.84, 0.91)	0.80	0.77
	PCU	0.76 (0.71, 0.80)	0.70	0.69

Table S7-5: Performance results of the models trained on KK-hospital and tested on KK-hospital data for General Hospital (GH), Primary care unit (PCU) scenario. The columns Sensitivity and Specificity were both measured at the specified threshold of 0.5.

Model	Scenario	AUC (95% CI)	Sensitivity	Specificity
1-Day	GH	0.83 (0.78, 0.87)	0.73	0.74
	PCU	0.72 (0.66, 0.78)	0.61	0.70
2-Day	GH	0.86 (0.81, 0.90)	0.77	0.76
	PCU	0.77 (0.72, 0.81)	0.69	0.70
3-Day	GH	0.89 (0.85, 0.93)	0.81	0.80
	PCU	0.76 (0.69, 0.82)	0.64	0.76

Table S7-6: Performance results of the models trained on KK-hospital and tested on SK-hospital data for General Hospital (GH), Primary care unit (PCU) scenario. The columns Sensitivity and Specificity were both measured at the specified threshold of 0.5.

Model	Scenario	AUC (95% CI)	Sensitivity	Specificity
1-Day	GH	0.74 (0.68, 0.79)	0.62	0.71
	PCU	0.57 (0.50, 0.63)	0.47	0.61
2-Day	GH	0.83 (0.79, 0.88)	0.76	0.75
	PCU	0.67 (0.60, 0.73)	0.72	0.50
3-Day	GH	0.86 (0.81, 0.91)	0.80	0.75
	PCU	0.76 (0.70, 0.83)	0.76	0.63

Table S7-7: Performance results of the models trained on SK-hospital and tested combined-site data for General hospital (GH), Primary care unit (PCU) scenario. The columns Sensitivity and Specificity were both measured at the specified threshold of 0.5.

**Model**	**Scenario**	**AUC (95% CI)**	**Sensitivity**	**Specificity**
1-Day	GH	0.77 (0.73, 0.81)	0.64	0.75
	PCU	0.62 (0.57, 0.66)	0.45	0.72
2-Day	GH	0.85 (0.82, 0.88)	0.71	0.81
	PCU	0.72 (0.68, 0.76)	0.56	0.75
3-Day	GH	0.88 (0.85, 0.91)	0.72	0.86
	PCU	0.76 (0.71, 0.81)	0.60	0.78

Table S7-8: Performance results of the models trained on SK-hospital and tested on KK-hospital data for General hospital (GH), Primary care unit (PCU) scenario. The columns Sensitivity and Specificity were both measured at the specified threshold of 0.5.

**Model**	**Scenario**	**AUC (95% CI)**	**Sensitivity**	**Specificity**
1-Day	GH	0.80 (0.75, 0.84)	0.66	0.77
	PCU	0.62 (0.55, 0.68)	0.39	0.77
2-Day	GH	0.84 (0.80, 0.88)	0.71	0.79
	PCU	0.71 (0.65, 0.76)	0.52	0.77
3-Day	GH	0.86 (0.81, 0.91)	0.68	0.85
	PCU	0.74 (0.67, 0.81)	0.58	0.78

Table S7-9: Performance results of the models trained on SK-hospital and tested on SK-hospital data for General hospital (GH), Primary care unit (PCU) scenario. The columns Sensitivity and Specificity were both measured at the specified threshold of 0.5.

Model	Scenario	AUC (95% CI)	Sensitivity	Specificity
1-Day	GH	0.75 (0.70, 0.79)	0.62	0.73
	PCU	0.62 (0.56, 0.68)	0.51	0.69
2-Day	GH	0.85 (0.81, 0.89)	0.71	0.83
	PCU	0.73 (0.68, 0.78)	0.59	0.73
3-Day	GH	0.90 (0.86, 0.94)	0.75	0.87
	PCU	0.78 (0.72, 0.84)	0.63	0.78
