# Supplementary material for: Prognostic prediction of dengue hemorrhagic fever in pediatric patients with suspected dengue infection: A multi-site study
Source: PLoS One. 2025 Aug 4;20(8):e0327360. doi: 10.1371/journal.pone.0327360 (PMC12321061; doi:10.1371/journal.pone.0327360)
Supplement: S8 File — (PDF) [file pone.0327360.s008.pdf]

Supplement file 8

The patient distribution by PCR and ELISA results for dengue serotypes in the cohort is as follows: 301 tested negative, with 510, 390, 314, and 215 positive for DENV 1, DENV 2, DENV 3, and DENV 4, respectively. There were also 4 un-interpretable results and 2 unavailable results. For primary and secondary infections, based on PCR and ELISA results, there were 55 Primary and 1518 Secondary cases. Additionally, 145 cases were Negative, with 3 Un-interpretable, 2 Not available, and 10 classified as Other.

Table S8-1: Summary of ELISA Test Results for Dengue Diagnosis Among Patients

**ELISA Test Result**	**# Patients**
Primary	55
Secondary	1518
Negative	145
Un-interpretable	3
Not available	2
Other	10

Table S8-2: Distribution of PCR Test Results for Dengue Virus Serotypes Among Patients

**PCR Test Result**	**# Patients**
Negative	300
DENV 1	509
DENV 2	389
DENV 3	314
DENV 4	215
Un-interpretable	4
Not available	2
