# Supplementary material for: Prognostic prediction of dengue hemorrhagic fever in pediatric patients with suspected dengue infection: A multi-site study
Source: PLoS One. 2025 Aug 4;20(8):e0327360. doi: 10.1371/journal.pone.0327360 (PMC12321061; doi:10.1371/journal.pone.0327360)
Supplement: S9 File — (PDF) [file pone.0327360.s009.pdf]

Supplement file 9

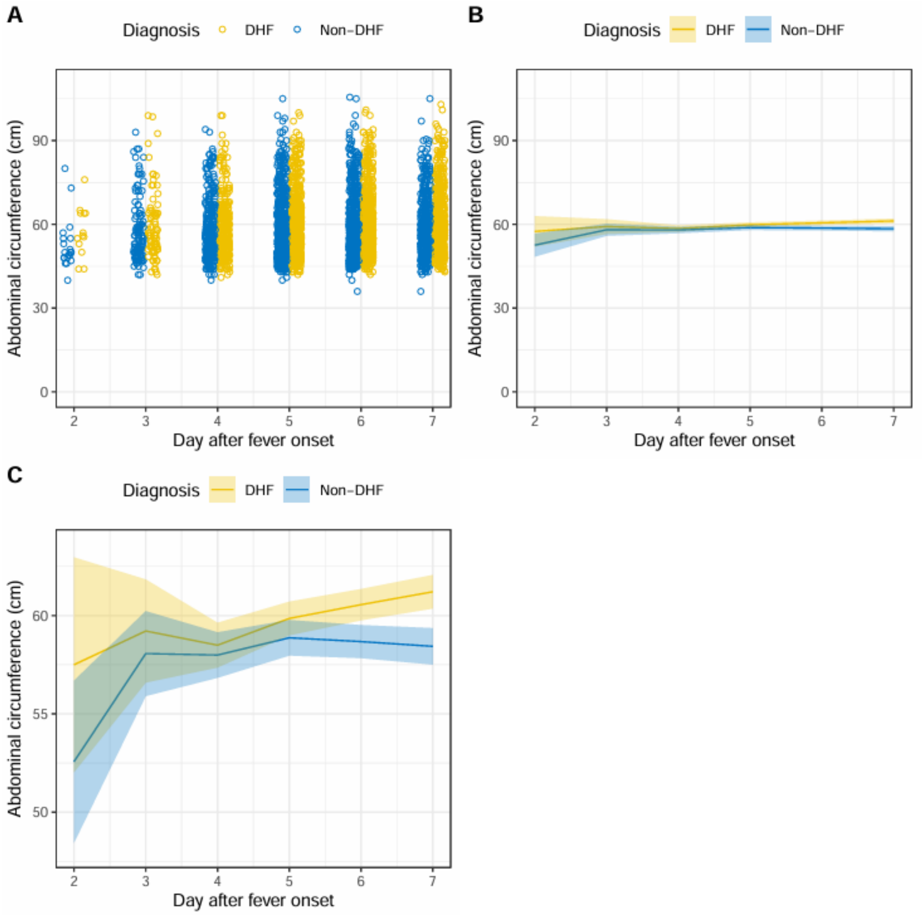

Fig. S9-1: Time course of `Abdomial cirumferenece` variable from two to seven days after fever onset in two groups (DHF and Non-DHF). The data are shown as raw values (A) and means grouped by day after fever onset (B) with the shaded areas representing 95% confident intervals of the means. The mean values (B) are also zoomed in to show the difference and trajectories two groups (C).

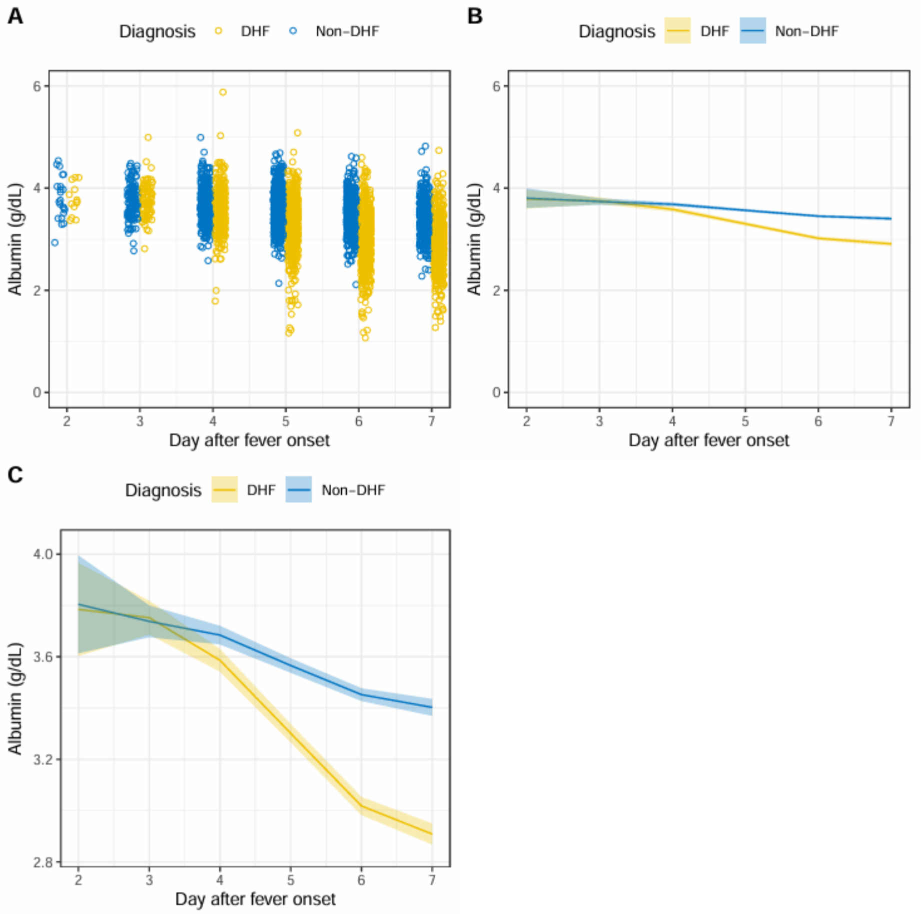

Fig. S9-2: Time course of `Albumin` variable from two to seven days after fever onset in two groups (DHF and Non-DHF). The data are shown as raw values (A) and means grouped by day after fever onset (B) with the shaded areas representing 95% confident intervals of the means. The mean values (B) are also zoomed in to show the difference and trajectories two groups (C).

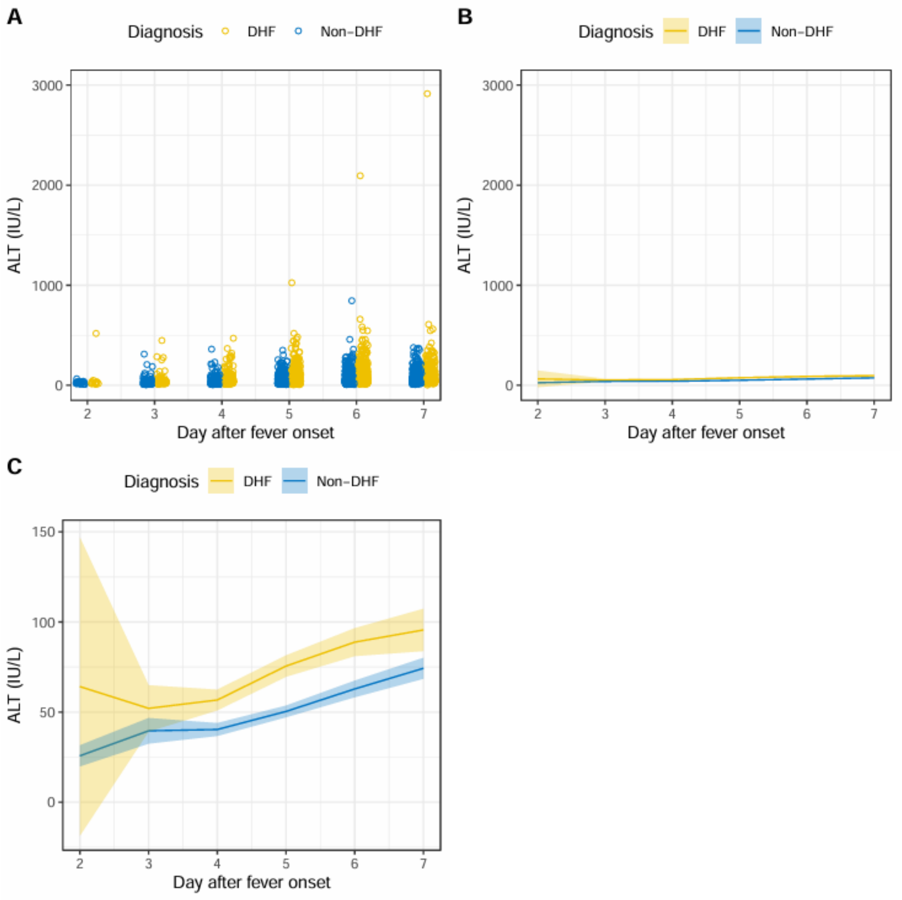

Fig. S9-3: Time course of `ALT` variable from two to seven days after fever onset in two groups (DHF and Non-DHF). The data are shown as raw values (A) and means grouped by day after fever onset (B) with the shaded areas representing 95% confident intervals of the means. The mean values (B) are also zoomed in to show the difference and trajectories two groups (C).

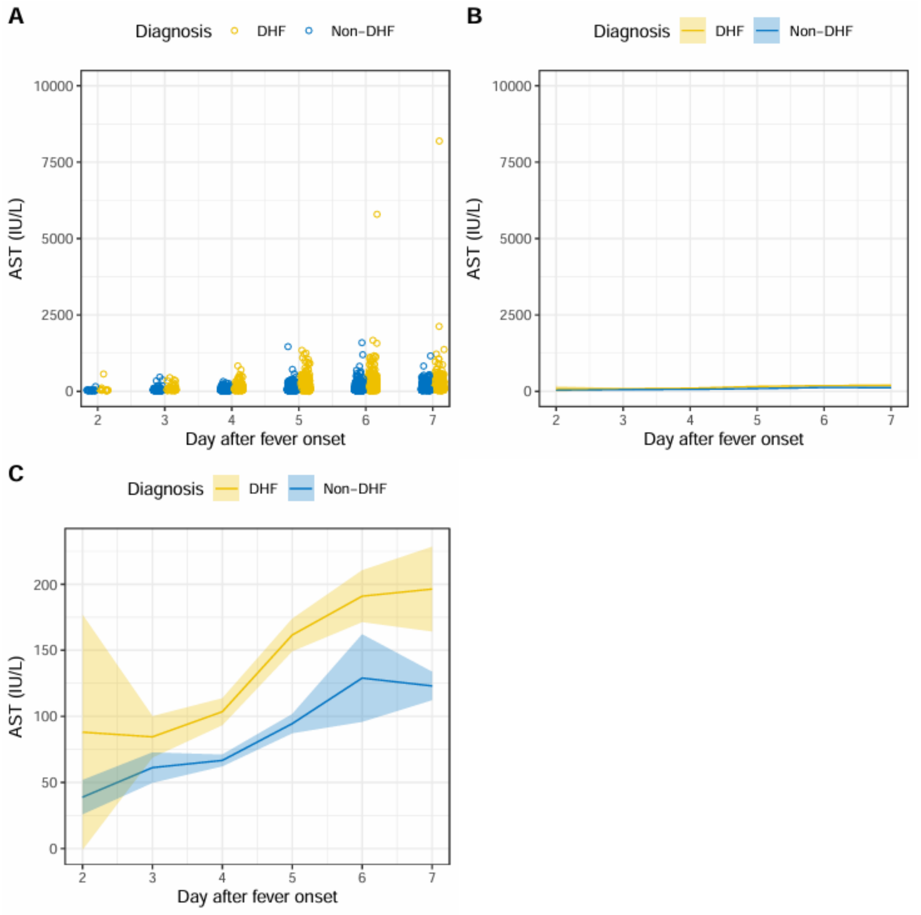

Fig. S9-4: Time course of `AST` variable from two to seven days after fever onset in two groups (DHF and Non-DHF). The data are shown as raw values (A) and means grouped by day after fever onset (B) with the shaded areas representing 95% confident intervals of the means. The mean values (B) are also zoomed in to show the difference and trajectories two groups (C).

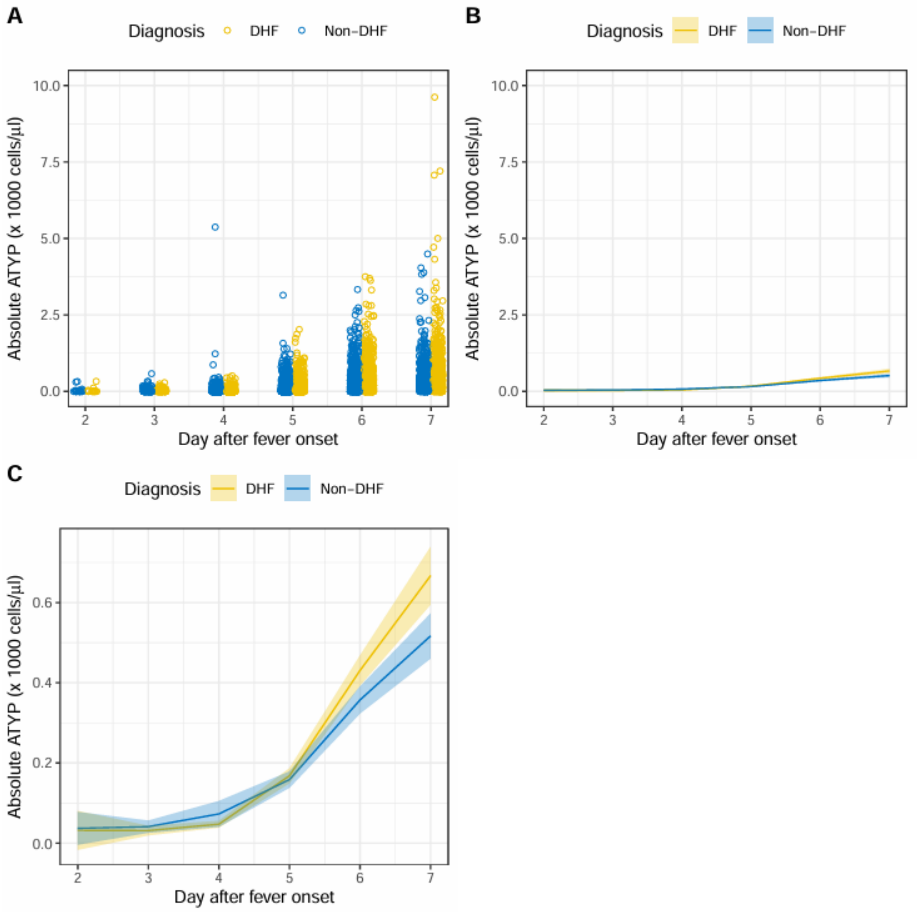

Fig. S9-5: Time course of `ATYP` variable from two to seven days after fever onset in two groups (DHF and Non-DHF). The data are shown as raw values (A) and means grouped by day after fever onset (B) with the shaded areas representing 95% confident intervals of the means. The mean values (B) are also zoomed in to show the difference and trajectories two groups (C).

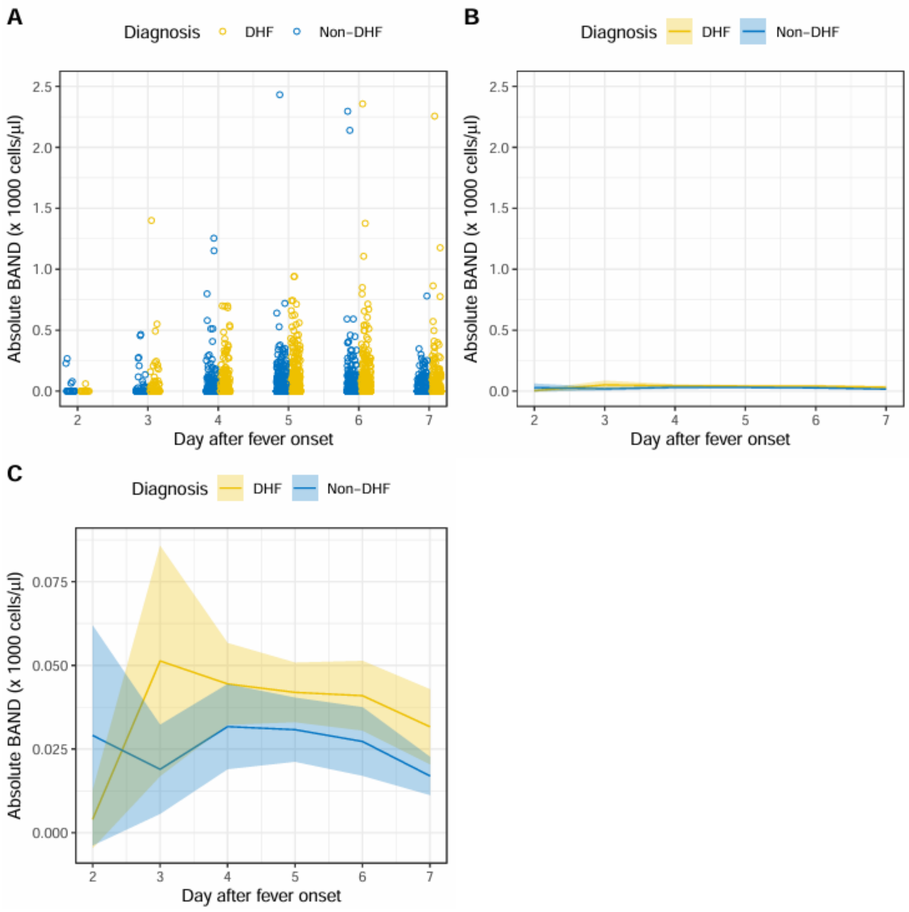

Fig. S9-6: Time course of `Band` variable from two to seven days after fever onset in two groups (DHF and Non-DHF). The data are shown as raw values (A) and means grouped by day after fever onset (B) with the shaded areas representing 95% confident intervals of the means. The mean values (B) are also zoomed in to show the difference and trajectories two groups (C).

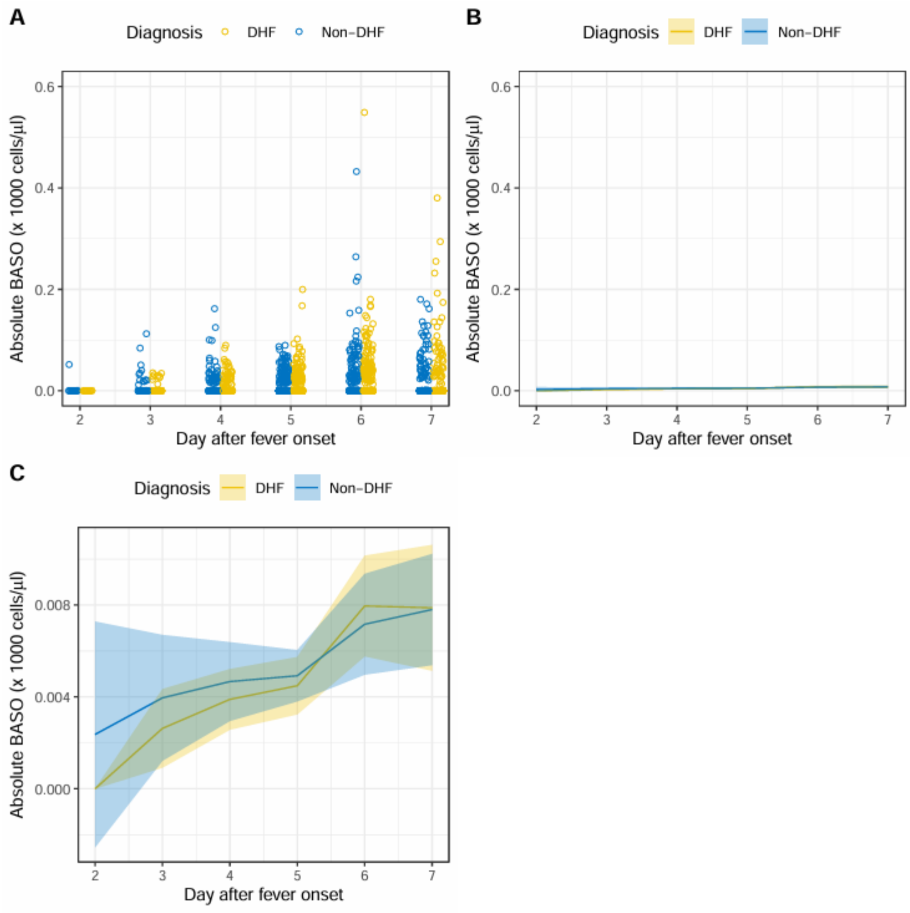

Fig. S9-7: Time course of `BASO` variable from two to seven days after fever onset in two groups (DHF and Non-DHF). The data are shown as raw values (A) and means grouped by day after fever onset (B) with the shaded areas representing 95% confident intervals of the means. The mean values (B) are also zoomed in to show the difference and trajectories two groups (C).

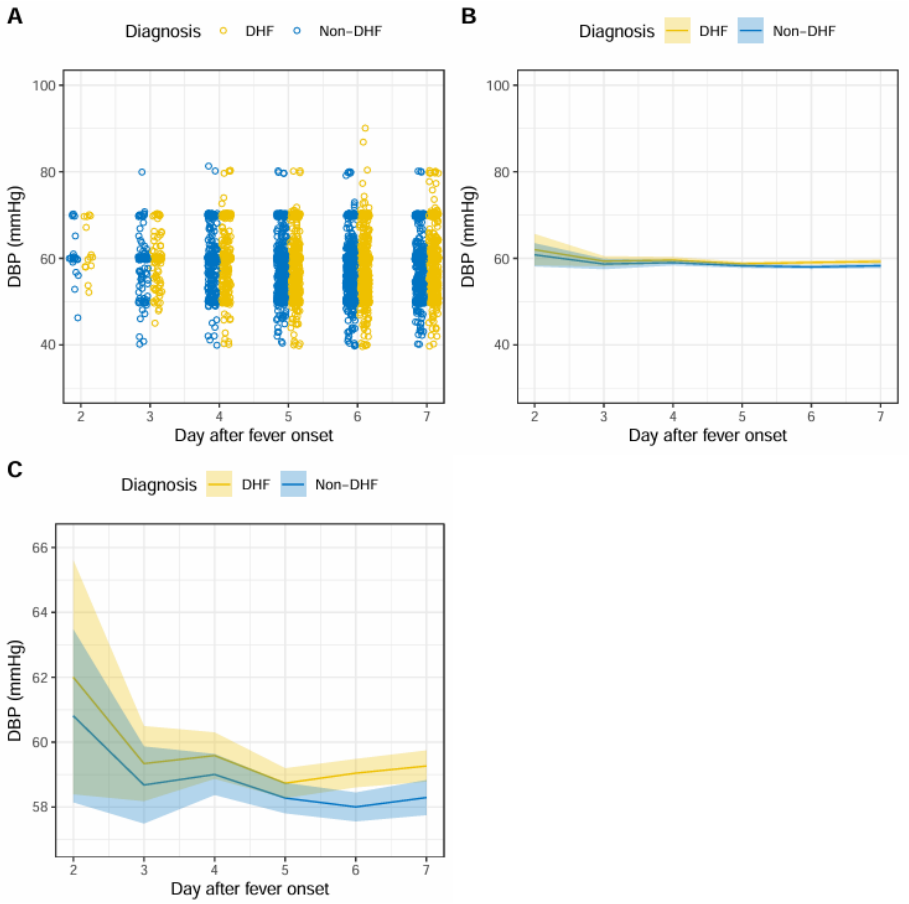

Fig. S9-8: Time course of DBP variable from two to seven days after fever onset in two groups (DHF and Non-DHF). The data are shown as raw values (A) and means grouped by day after fever onset (B) with the shaded areas representing 95% confident intervals of the means. The mean values (B) are also zoomed in to show the difference and trajectories two groups (C).

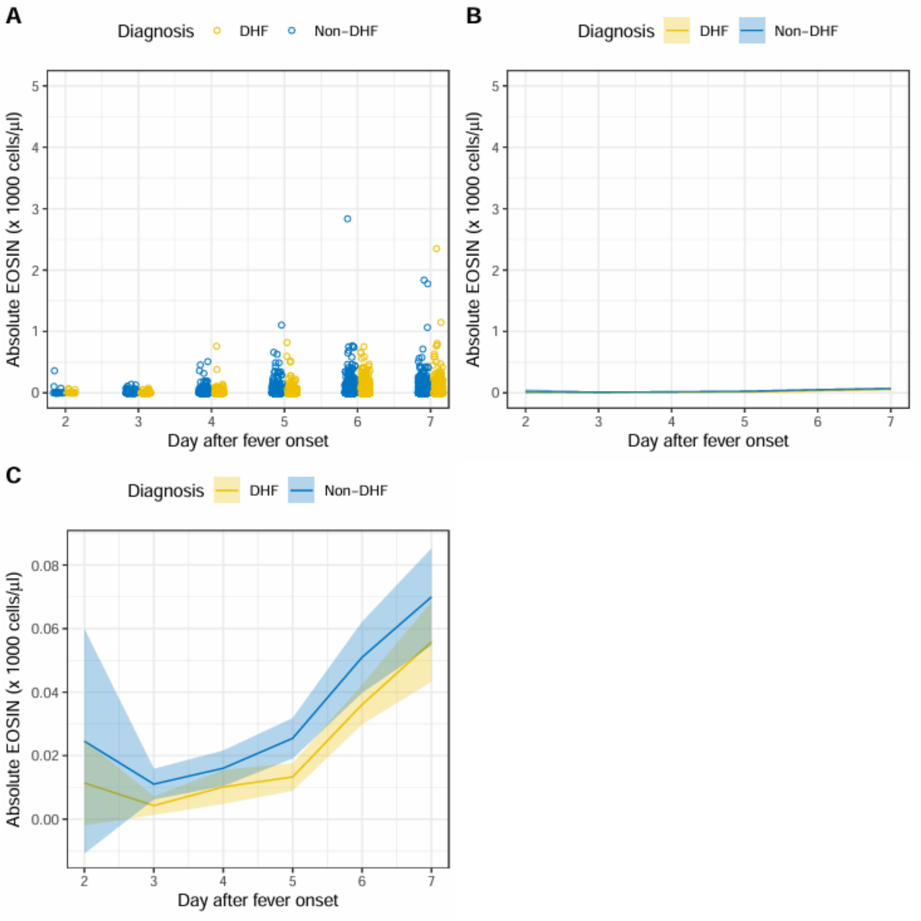

Fig. S9-9: Time course of `EOSIN` variable from two to seven days after fever onset in two groups (DHF and Non-DHF). The data are shown as raw values (A) and means grouped by day after fever onset (B) with the shaded areas representing 95% confident intervals of the means. The mean values (B) are also zoomed in to show the difference and trajectories two groups (C).

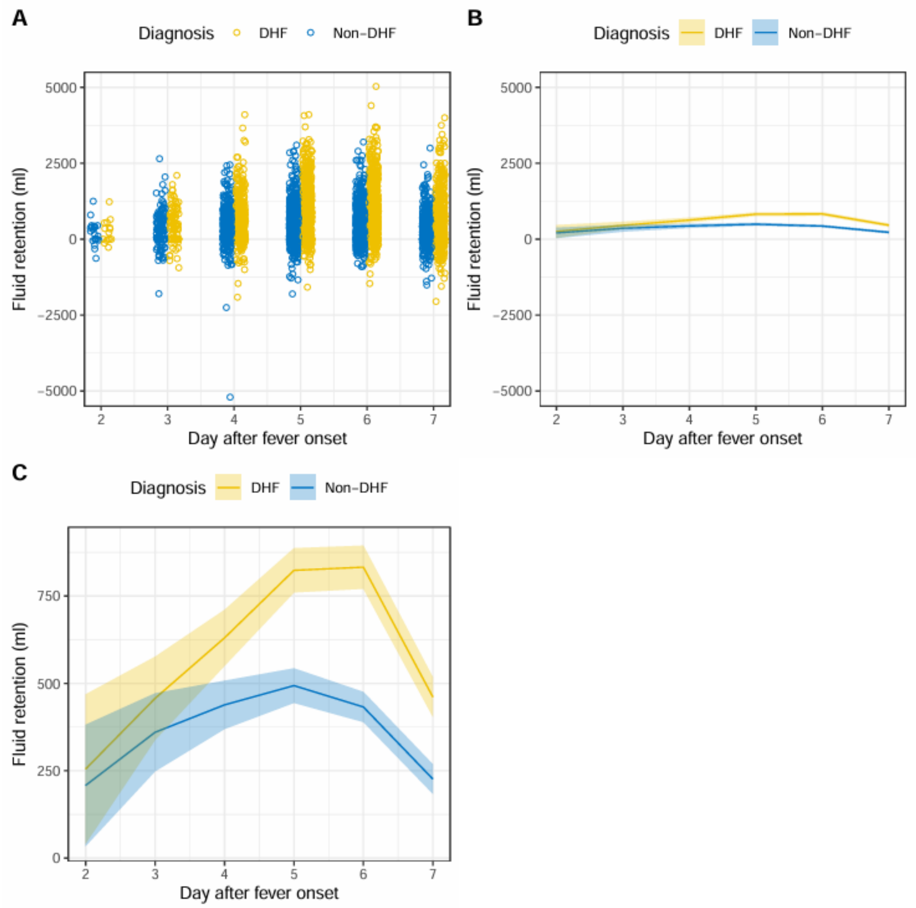

Fig. S9-10: Time course of `Fluid retention` variable from two to seven days after fever onset in two groups (DHF and Non-DHF). The data are shown as raw values (A) and means grouped by day after fever onset (B) with the shaded areas representing 95% confident intervals of the means. The mean values (B) are also zoomed in to show the difference and trajectories two groups (C).

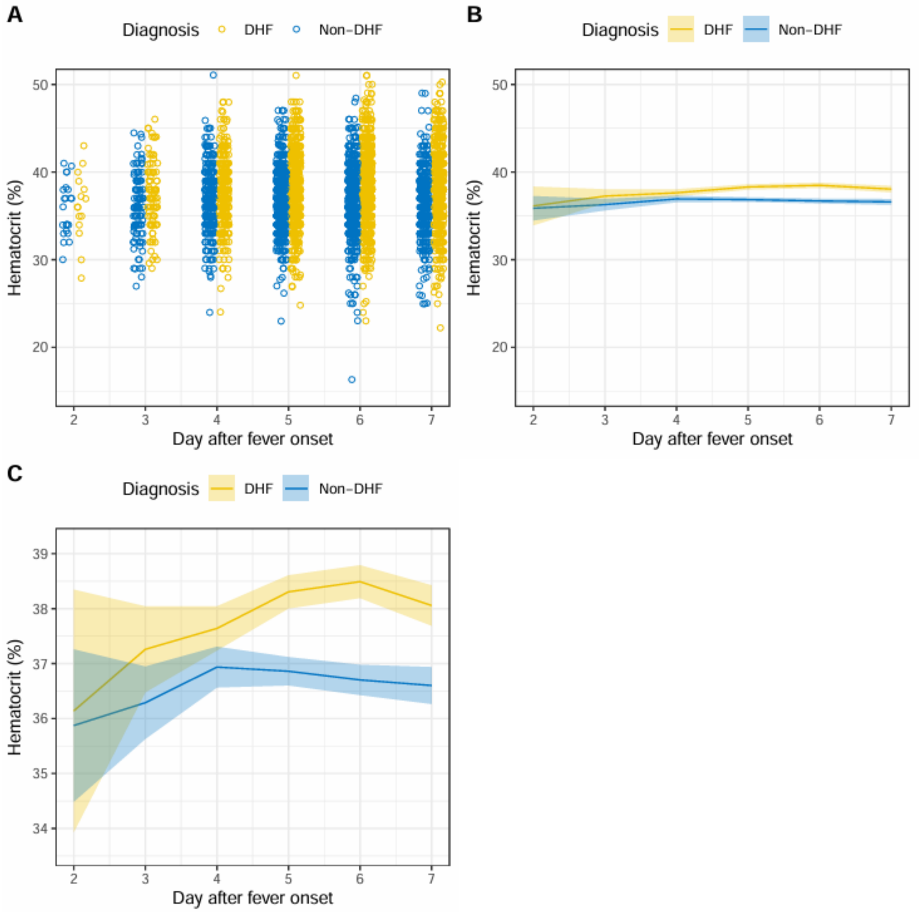

Fig. S9-11: Time course of `hematocrit` variable from two to seven days after fever onset in two groups (DHF and Non-DHF). The data are shown as raw values (A) and means grouped by day after fever onset (B) with the shaded areas representing 95% confident intervals of the means. The mean values (B) are also zoomed in to show the difference and trajectories two groups (C).

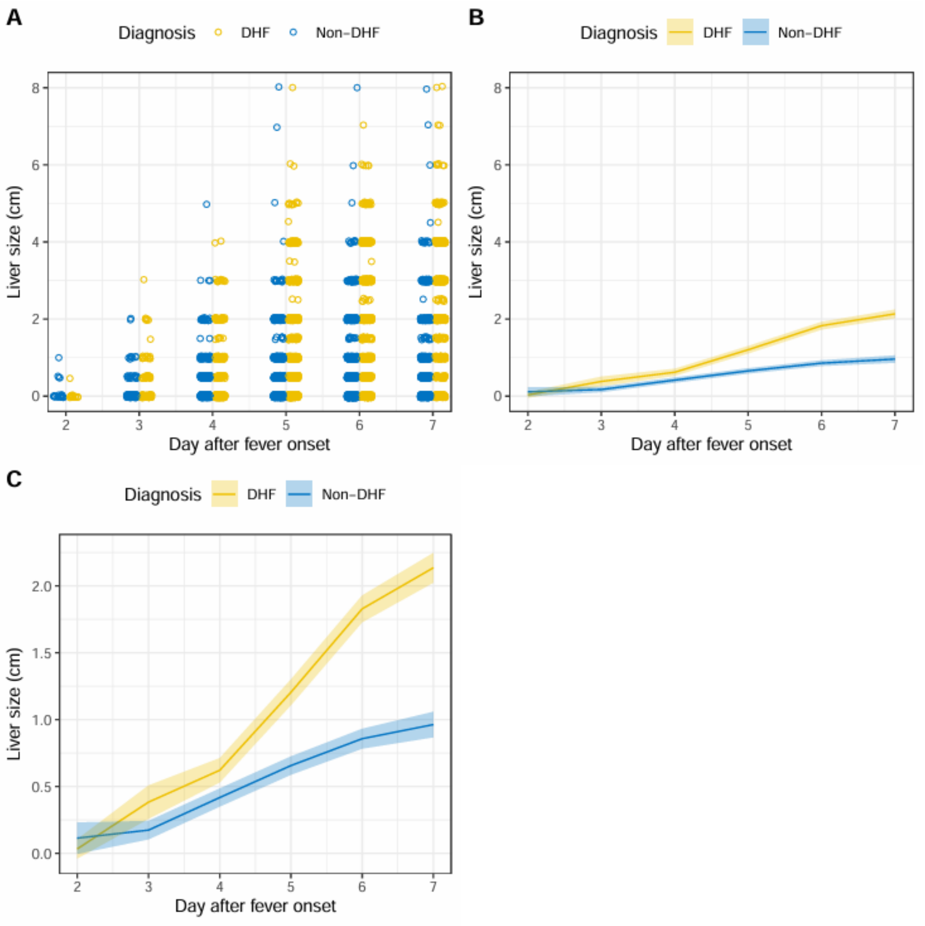

Fig. S9-12: Time course of `Liver size` variable from two to seven days after fever onset in two groups (DHF and Non-DHF). The data are shown as raw values (A) and means grouped by day after fever onset (B) with the shaded areas representing 95% confident intervals of the means. The mean values (B) are also zoomed in to show the difference and trajectories two groups (C).

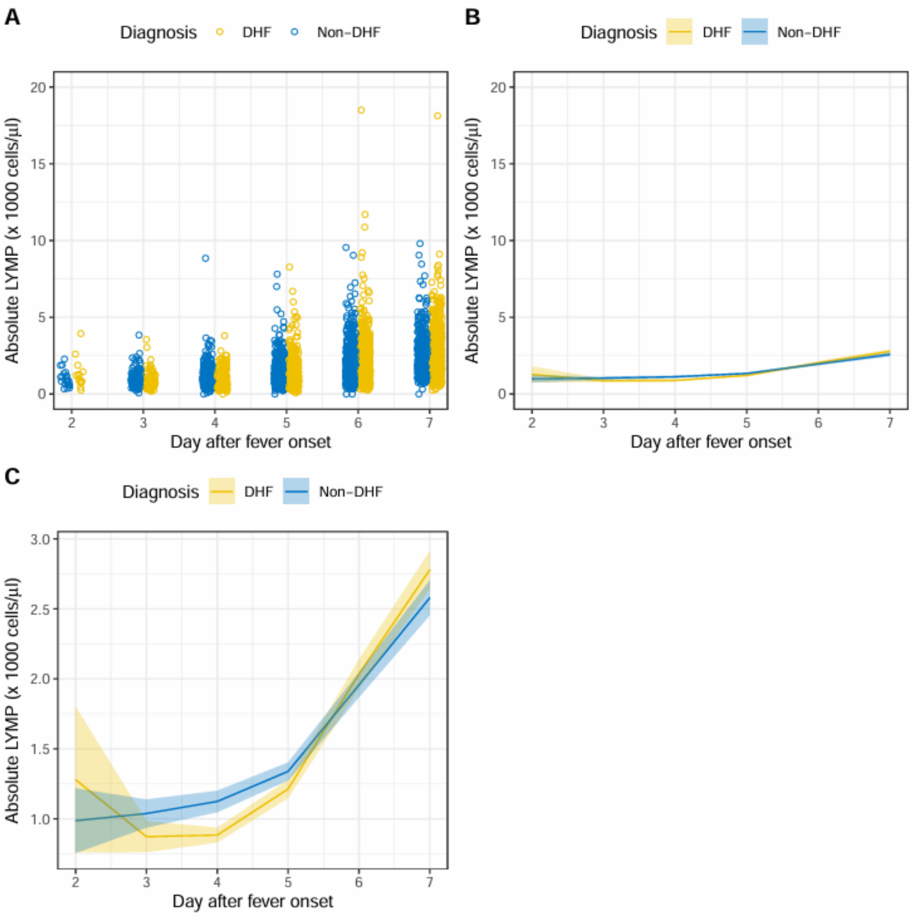

Fig. S9-13: Time course of `Lymphocyte` variable from two to seven days after fever onset in two groups (DHF and Non-DHF). The data are shown as raw values (A) and means grouped by day after fever onset (B) with the shaded areas representing 95% confident intervals of the means. The mean values (B) are also zoomed in to show the difference and trajectories two groups (C).

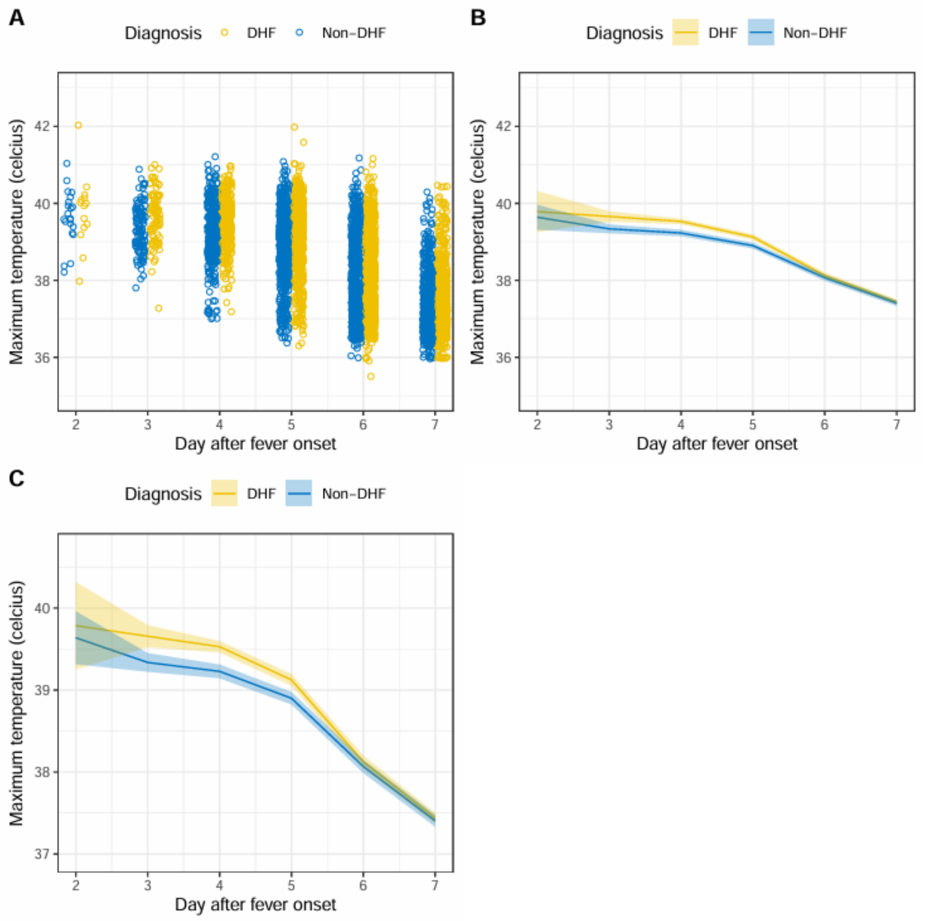

Fig. S9-14: Time course of `Maximum body temperature` variable from two to seven days after fever onset in two groups (DHF and Non-DHF). The data are shown as raw values (A) and means grouped by day after fever onset (B) with the shaded areas representing 95% confident intervals of the means. The mean values (B) are also zoomed in to show the difference and trajectories two groups (C).

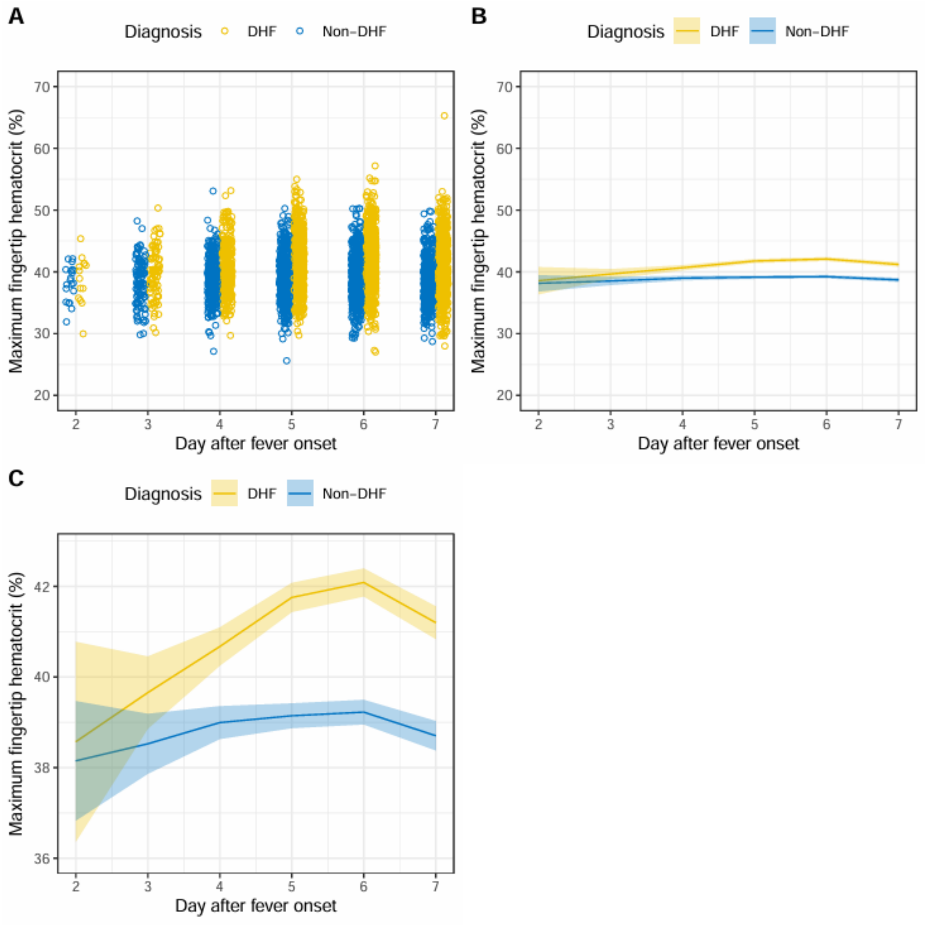

Fig. S9-15: Time course of `Maximum fingerip hematocrit` variable from two to seven days after fever onset in two groups (DHF and Non-DHF). The data are shown as raw values (A) and means grouped by day after fever onset (B) with the shaded areas representing 95% confident intervals of the means. The mean values (B) are also zoomed in to show the difference and trajectories two groups (C).

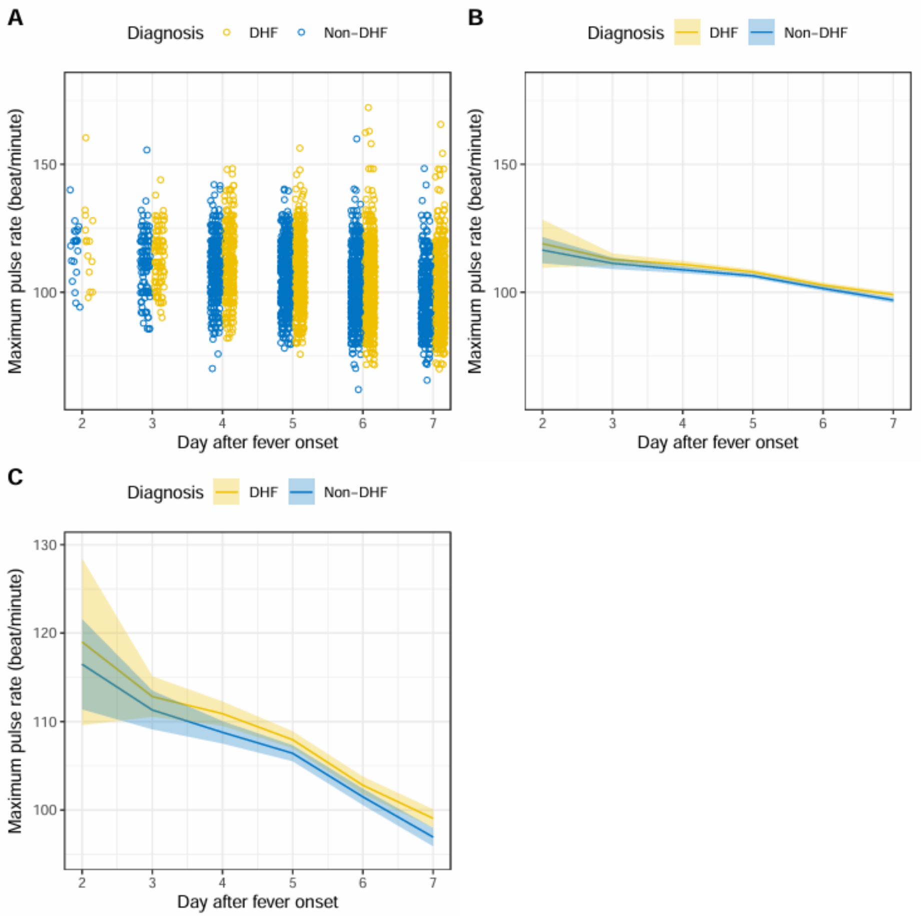

Fig. S9-16: Time course of `Maximum pulse rate` variable from two to seven days after fever onset in two groups (DHF and Non-DHF). The data are shown as raw values (A) and means grouped by day after fever onset (B) with the shaded areas representing 95% confident intervals of the means. The mean values (B) are also zoomed in to show the difference and trajectories two groups (C).

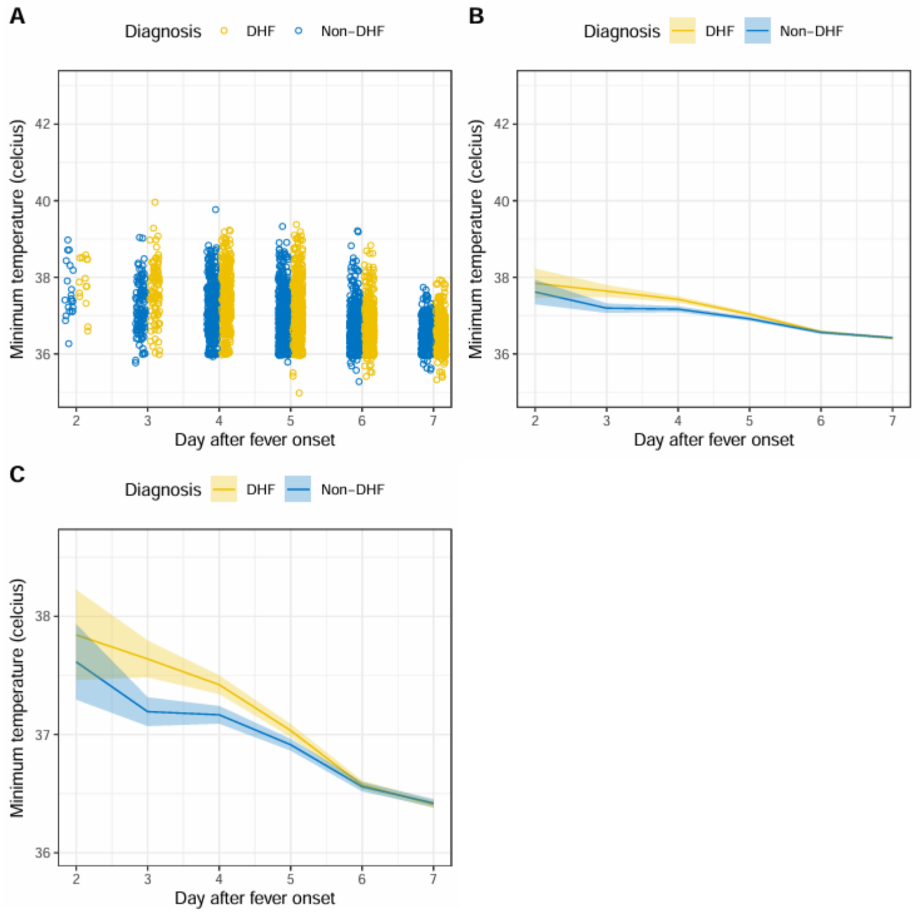

Fig. S9-17: Time course of `Minimum body temperature` variable from two to seven days after fever onset in two groups (DHF and Non-DHF). The data are shown as raw values (A) and means grouped by day after fever onset (B) with the shaded areas representing 95% confident intervals of the means. The mean values (B) are also zoomed in to show the difference and trajectories two groups (C).

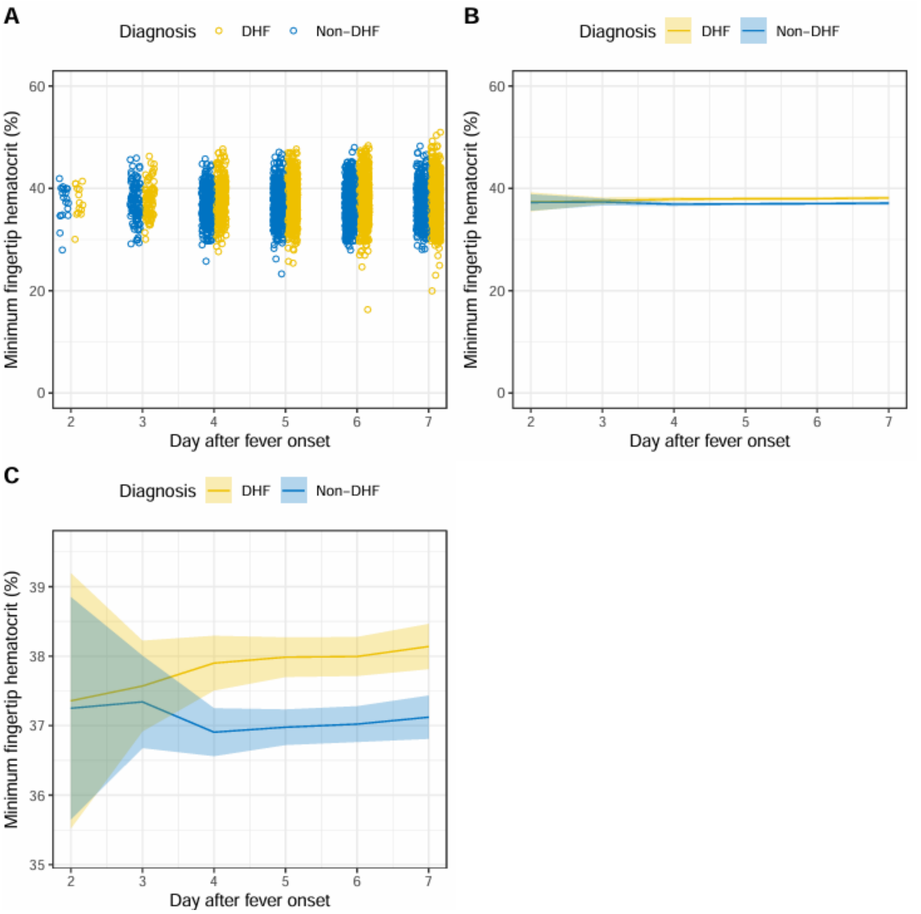

Fig. S9-18: Time course of `Minimum fingerip hematocrit` variable from two to seven days after fever onset in two groups (DHF and Non-DHF). The data are shown as raw values (A) and means grouped by day after fever onset (B) with the shaded areas representing 95% confident intervals of the means. The mean values (B) are also zoomed in to show the difference and trajectories two groups (C).

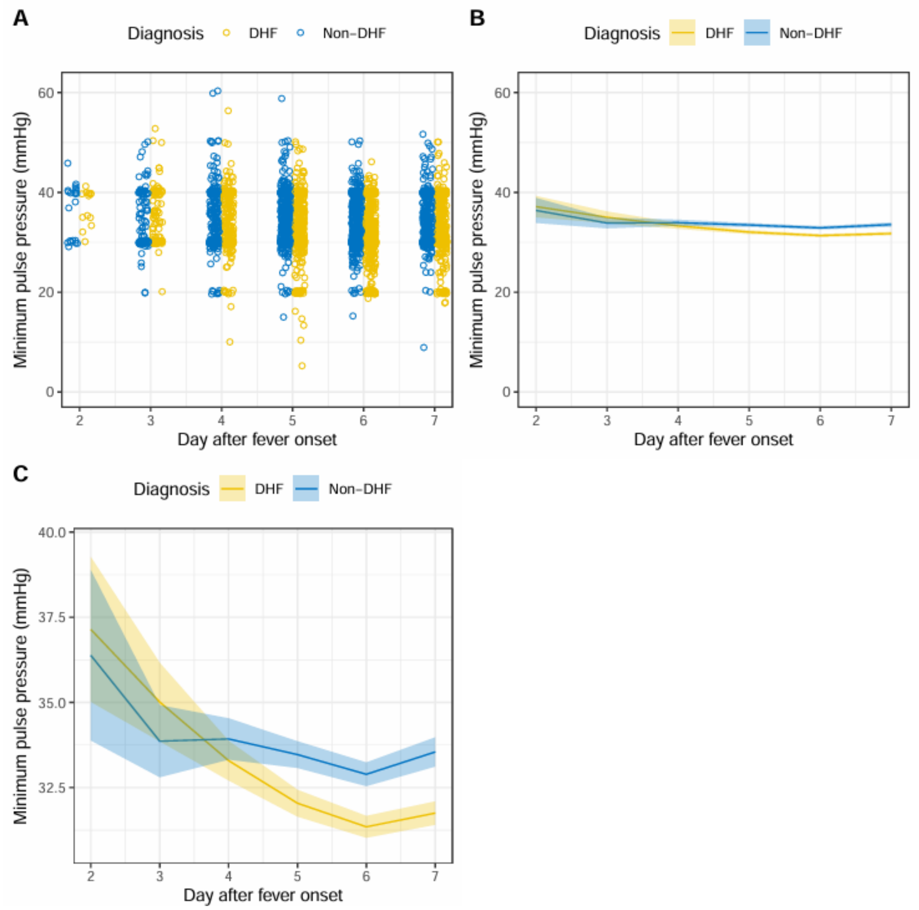

Fig. S9-19: Time course of `Minimum pulse pressure` variable from two to seven days after fever onset in two groups (DHF and Non-DHF). The data are shown as raw values (A) and means grouped by day after fever onset (B) with the shaded areas representing 95% confident intervals of the means. The mean values (B) are also zoomed in to show the difference and trajectories two groups (C).

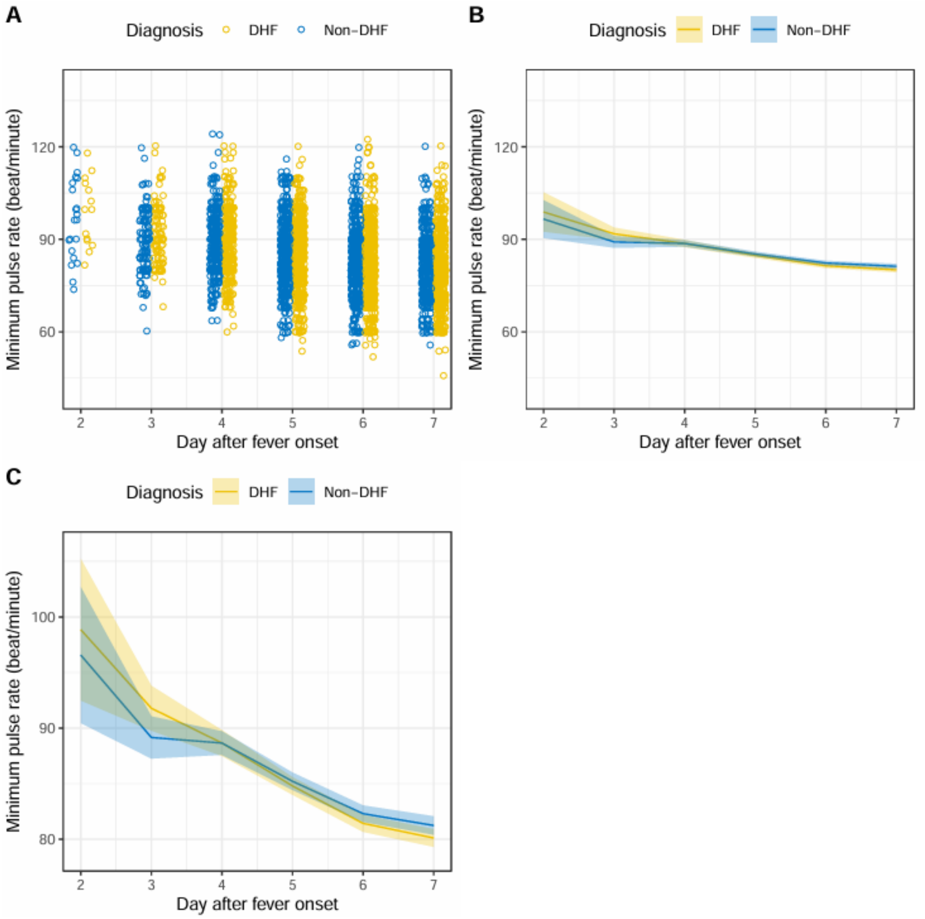

Fig. S9-20: Time course of `Minimum pulse rate` variable from two to seven days after fever onset in two groups (DHF and Non-DHF). The data are shown as raw values (A) and means grouped by day after fever onset (B) with the shaded areas representing 95% confident intervals of the means. The mean values (B) are also zoomed in to show the difference and trajectories two groups (C).

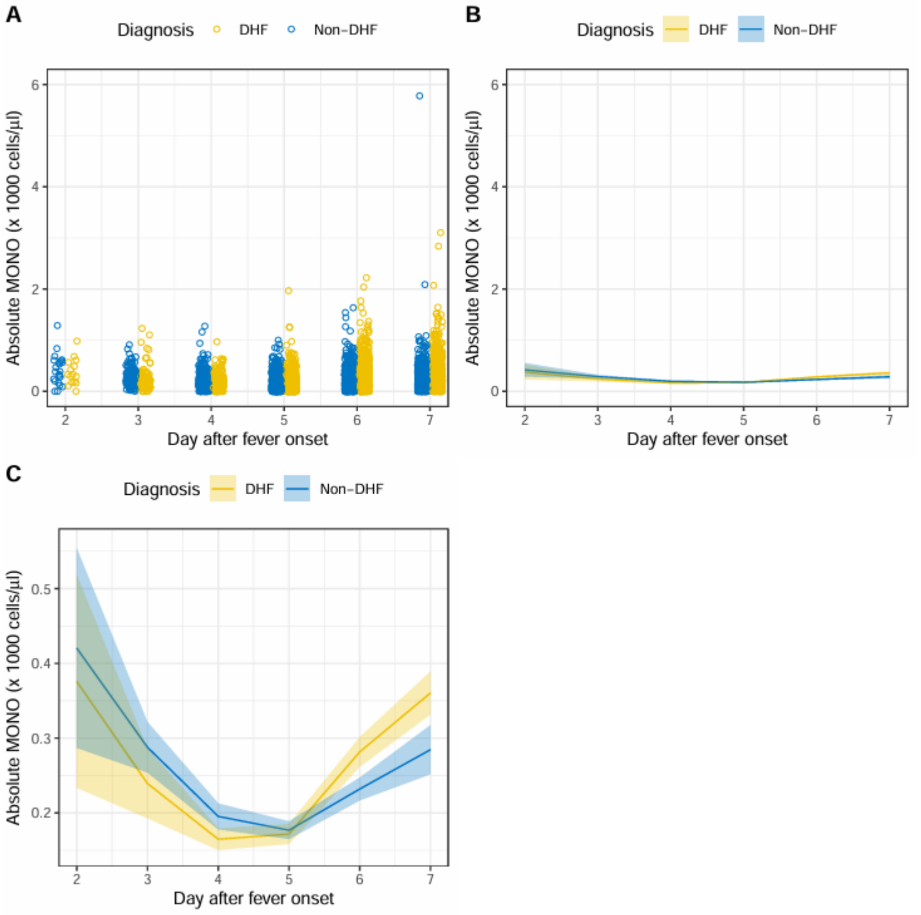

Fig. S9-21: Time course of `MONO` variable from two to seven days after fever onset in two groups (DHF and Non-DHF). The data are shown as raw values (A) and means grouped by day after fever onset (B) with the shaded areas representing 95% confident intervals of the means. The mean values (B) are also zoomed in to show the difference and trajectories two groups (C).

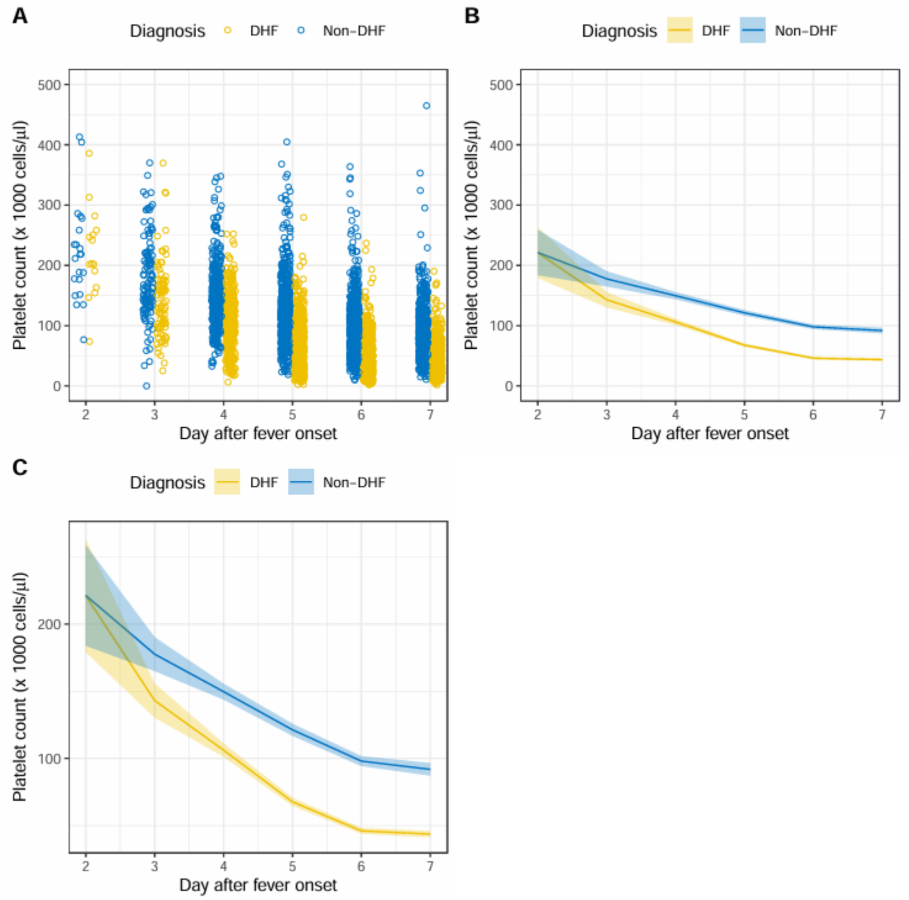

Fig. S9-22: Time course of `Platelet count` variable from two to seven days after fever onset in two groups (DHF and Non-DHF). The data are shown as raw values (A) and means grouped by day after fever onset (B) with the shaded areas representing 95% confident intervals of the means. The mean values (B) are also zoomed in to show the difference and trajectories two groups (C).

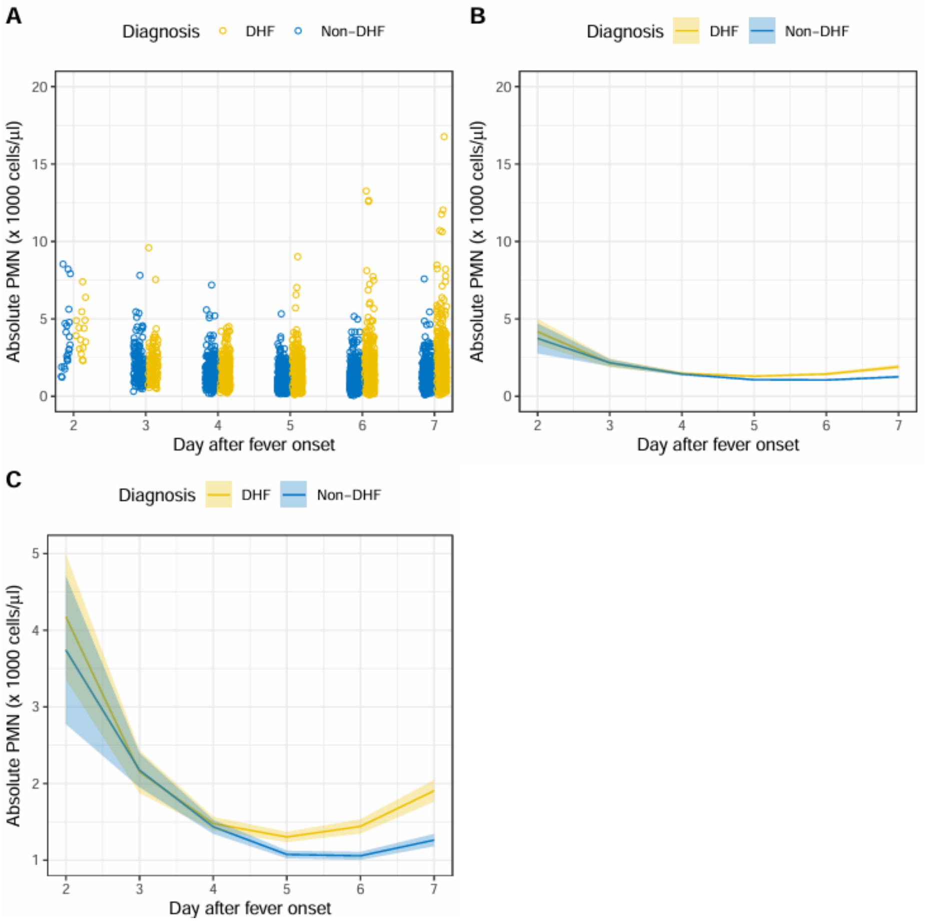

Fig. S9-23: Time course of `PMN` variable from two to seven days after fever onset in two groups (DHF and Non-DHF). The data are shown as raw values (A) and means grouped by day after fever onset (B) with the shaded areas representing 95% confident intervals of the means. The mean values (B) are also zoomed in to show the difference and trajectories two groups (C).

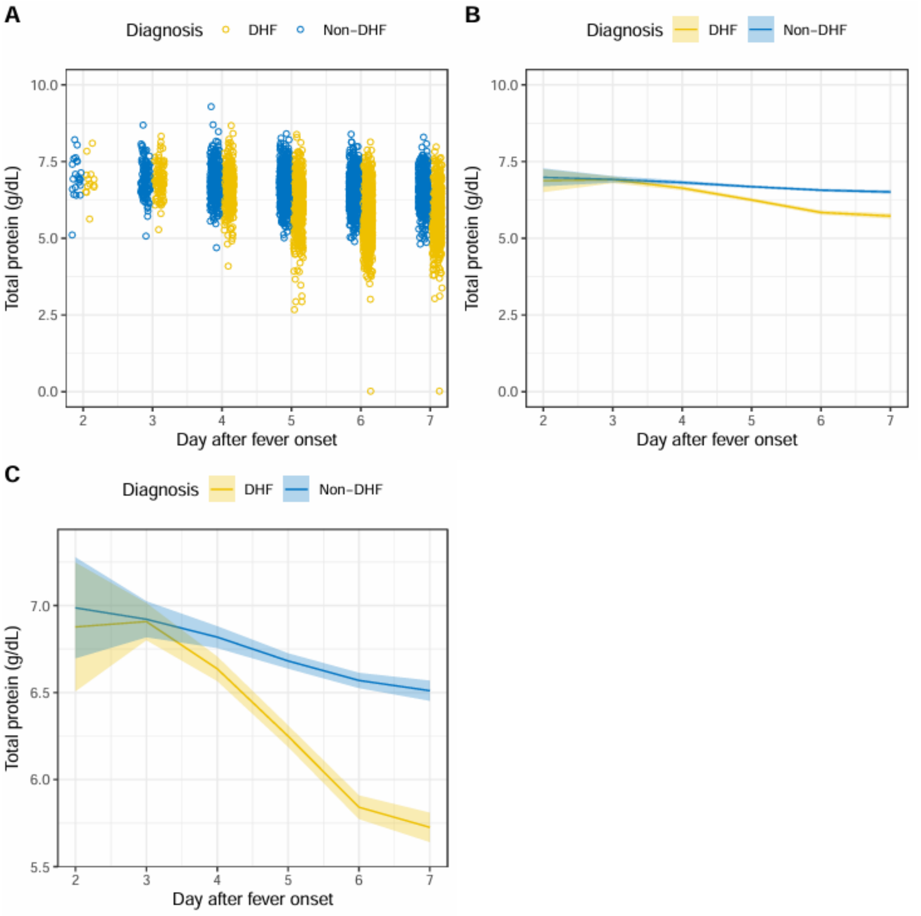

Fig. S9-24: Time course of `Protein` variable from two to seven days after fever onset in two groups (DHF and Non-DHF). The data are shown as raw values (A) and means grouped by day after fever onset (B) with the shaded areas representing 95% confident intervals of the means. The mean values (B) are also zoomed in to show the difference and trajectories two groups (C).

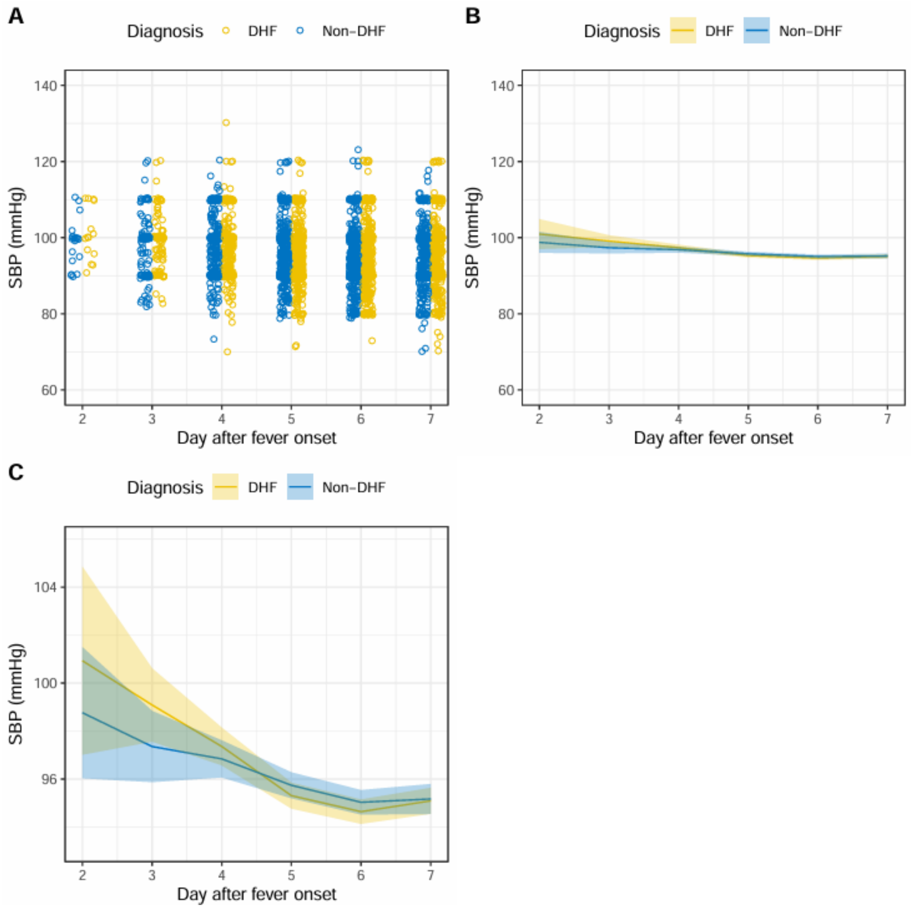

Fig. S9-25: Time course of `SBP` variable from two to seven days after fever onset in two groups (DHF and Non-DHF). The data are shown as raw values (A) and means grouped by day after fever onset (B) with the shaded areas representing 95% confident intervals of the means. The mean values (B) are also zoomed in to show the difference and trajectories two groups (C).

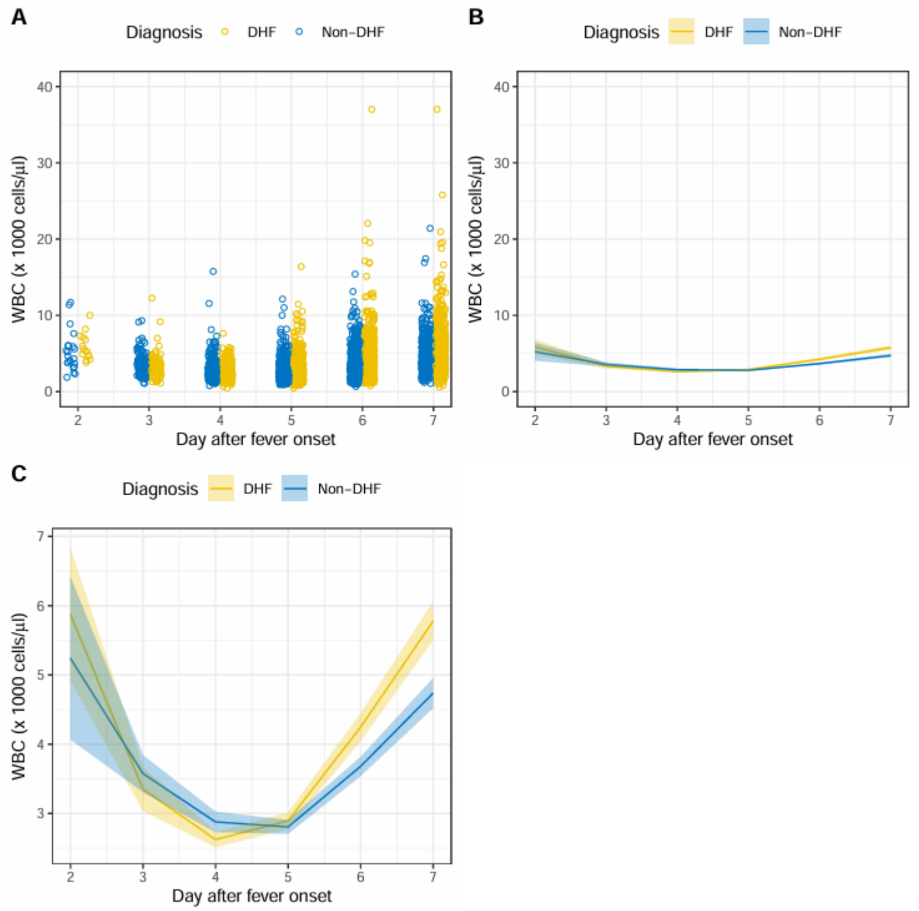

Fig. S9-26: Time course of `WBC` variable from two to seven days after fever onset in two groups (DHF and Non-DHF). The data are shown as raw values (A) and means grouped by day after fever onset (B) with the shaded areas representing 95% confident intervals of the means. The mean values (B) are also zoomed in to show the difference and trajectories two groups (C).

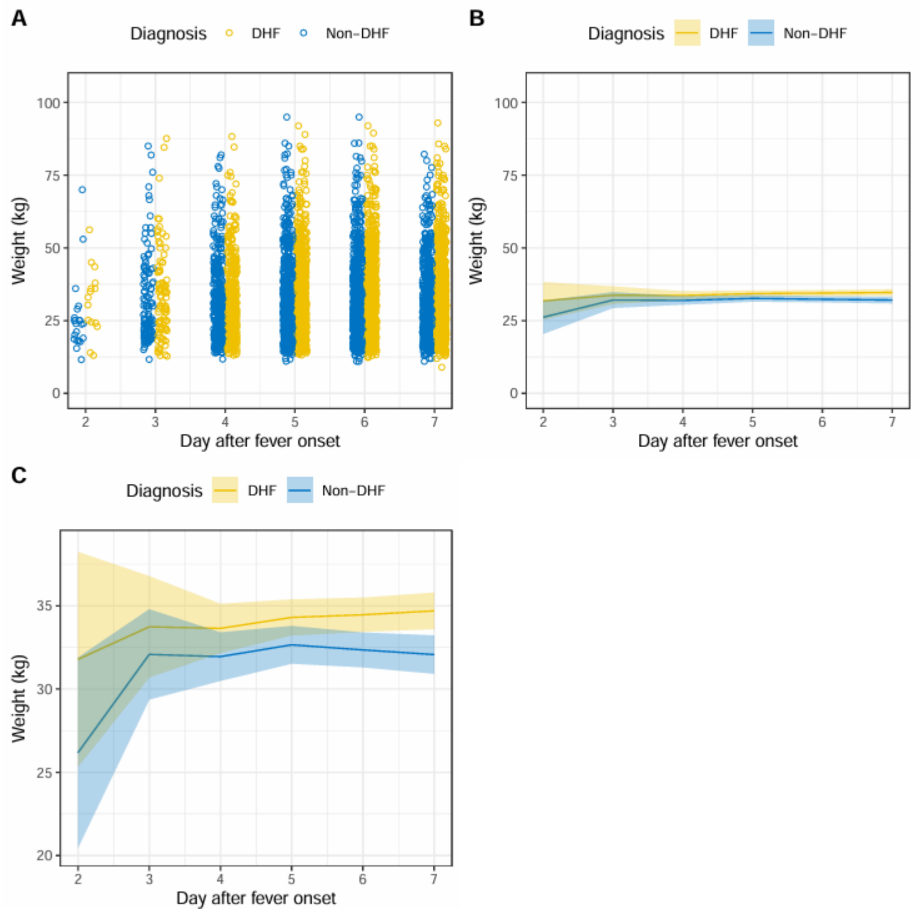

Fig. S9-27: Time course of `Weight` variable from two to seven days after fever onset in two groups (DHF and Non-DHF). The data are shown as raw values (A) and means grouped by day after fever onset (B) with the shaded areas representing 95% confident intervals of the means. The mean values (B) are also zoomed in to show the difference and trajectories two groups (C).
