# Supplementary material for: Prognostic prediction of dengue hemorrhagic fever in pediatric patients with suspected dengue infection: A multi-site study
Source: PLoS One. 2025 Aug 4;20(8):e0327360. doi: 10.1371/journal.pone.0327360 (PMC12321061; doi:10.1371/journal.pone.0327360)
Supplement: S10 File — (PDF) [file pone.0327360.s010.pdf]

Supplement file 10

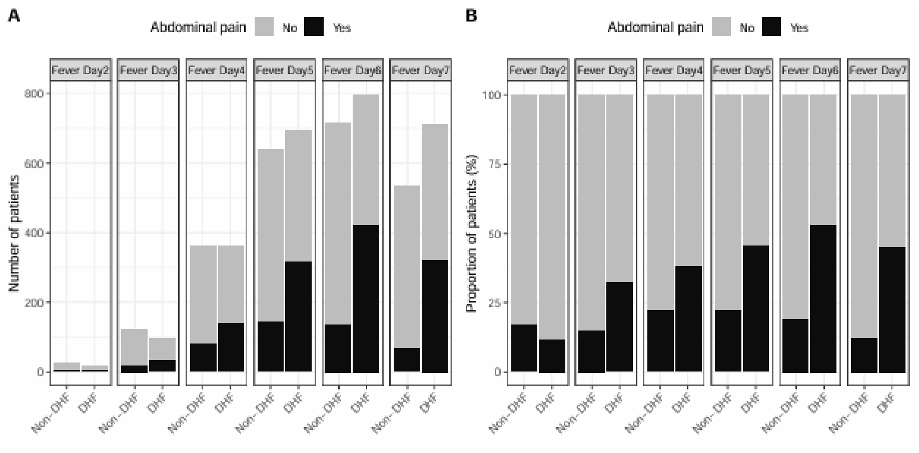

Fig. S10-1: Time course of `Abdominal pain` from two to seven days after fever onset in two groups (DHF and Non-DHF). The data are shown as raw counts (A) and proportions by group by study day (B).

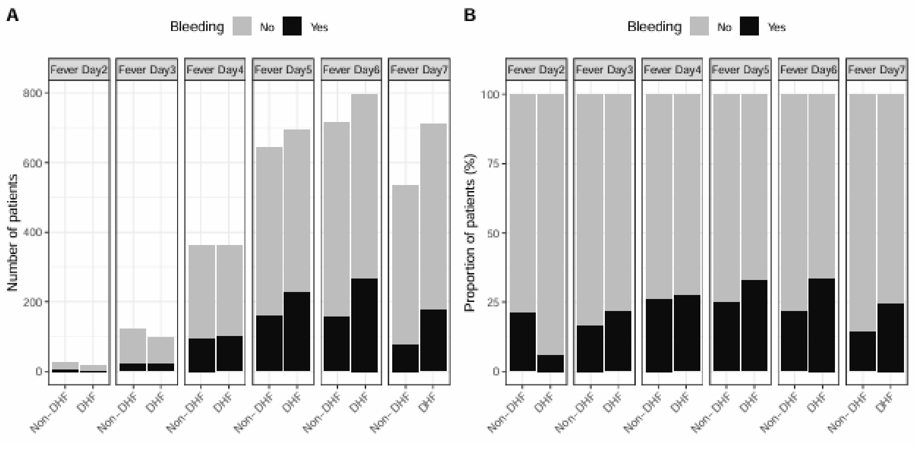

Fig. S10-2: Time course of `Bleeding` from two to seven days after fever onset in two groups (DHF and Non-DHF). The data are shown as raw counts (A) and proportions by group by study day (B).

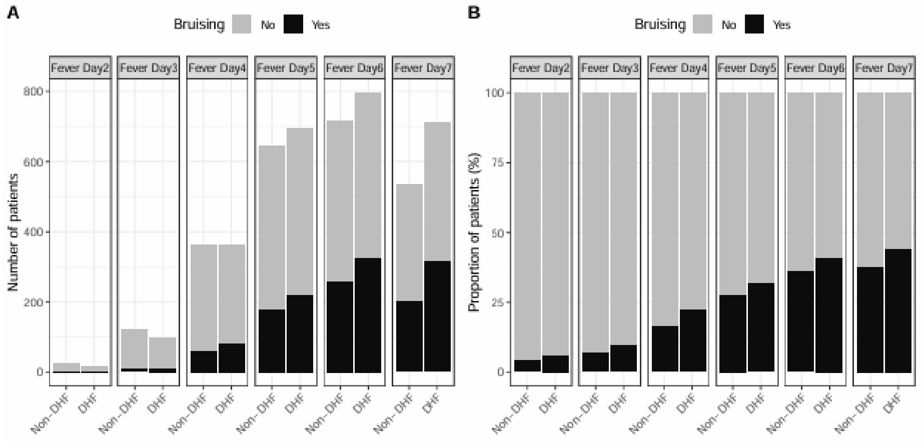

Fig. S10-3: Time course of `Bruising` from two to seven days after fever onset in two groups (DHF and Non-DHF). The data are shown as raw counts (A) and proportions by group by study day (B).

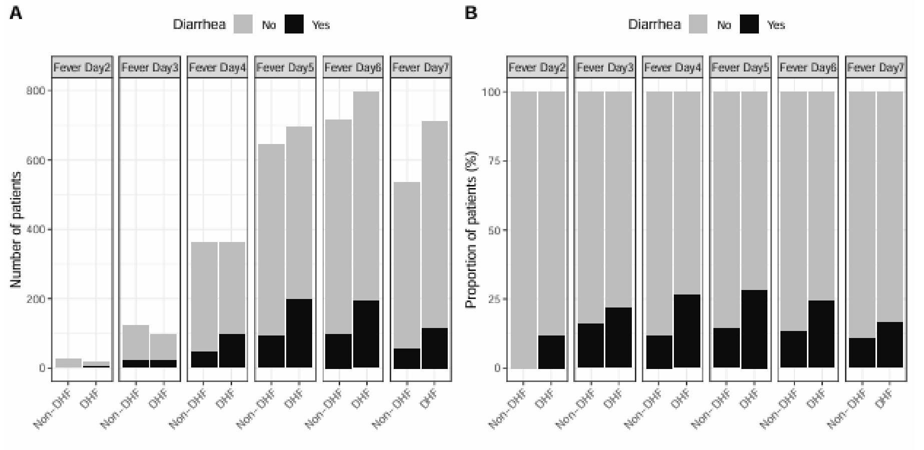

Fig. S10-4: Time course of `Diarrhea` from two to seven days after fever onset in two groups (DHF and Non-DHF). The data are shown as raw counts (A) and proportions by group by study day (B).

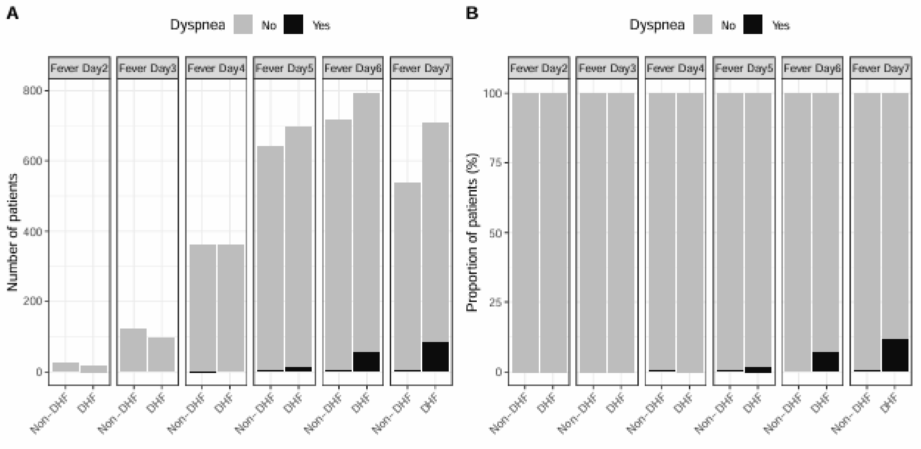

Fig. S10-5: Time course of `Dyspnea` from two to seven days after fever onset in two groups (DHF and Non-DHF). The data are shown as raw counts (A) and proportions by group by study day (B).

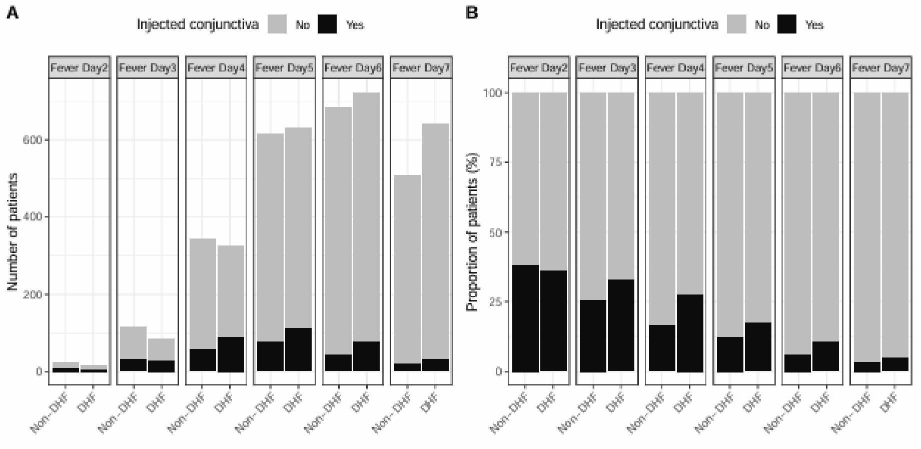

Fig. S10-6: Time course of `Injected conjunctiva` from two to seven days after fever onset in two groups (DHF and Non-DHF). The data are shown as raw counts (A) and proportions by group by study day (B).

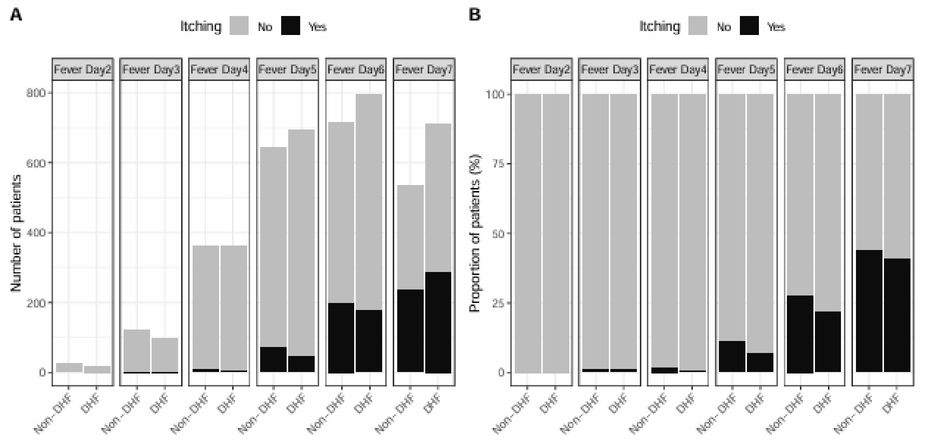

Fig. S10-7: Time course of `Itching` from two to seven days after fever onset in two groups (DHF and Non-DHF). The data are shown as raw counts (A) and proportions by group by study day (B).

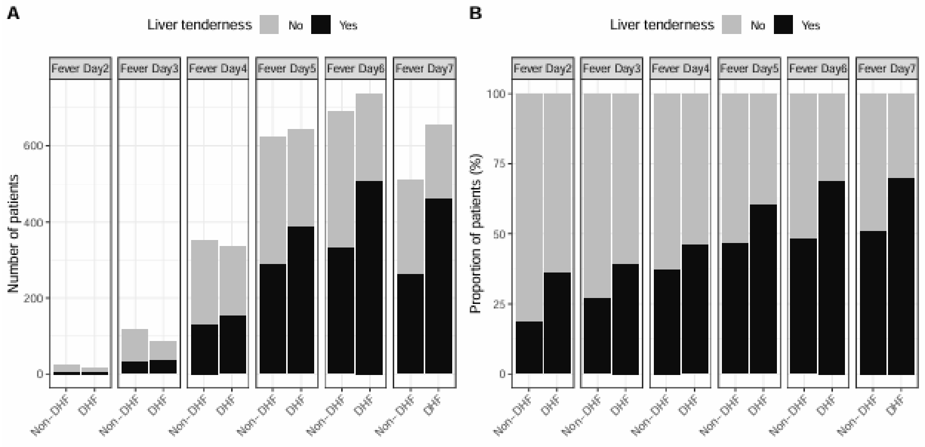

Fig. S10-8: Time course of `Liver tenderness` from two to seven days after fever onset in two groups (DHF and Non-DHF). The data are shown as raw counts (A) and proportions by group by study day (B).

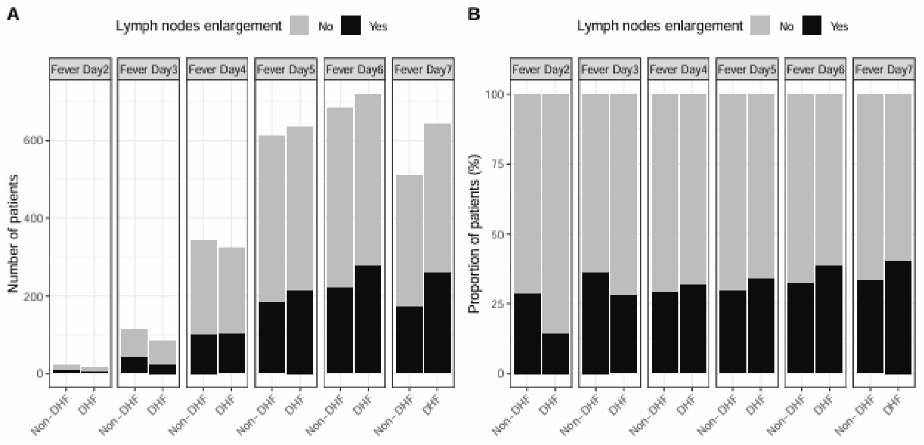

Fig. S10-9: Time course of `Lymph nodes enlargement` from two to seven days after fever onset in two groups (DHF and Non-DHF). The data are shown as raw counts (A) and proportions by group by study day (B).

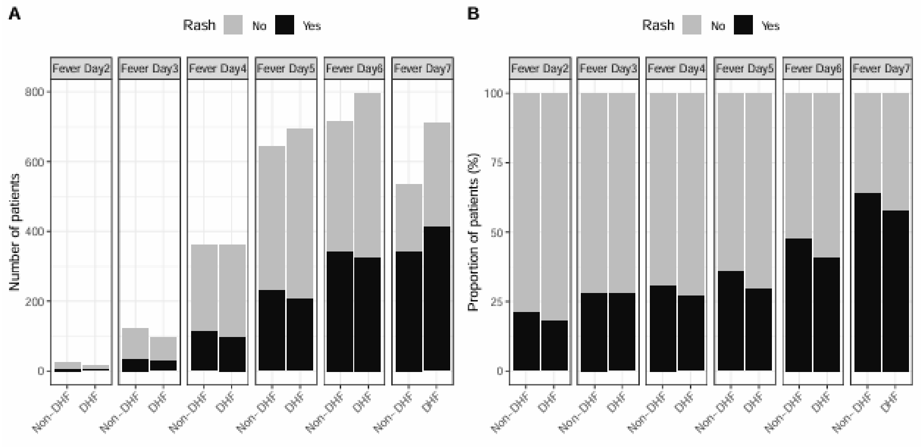

Fig. S10-10: Time course of `Rash` from two to seven days after fever onset in two groups (DHF and Non-DHF). The data are shown as raw counts (A) and proportions by group by study day (B).

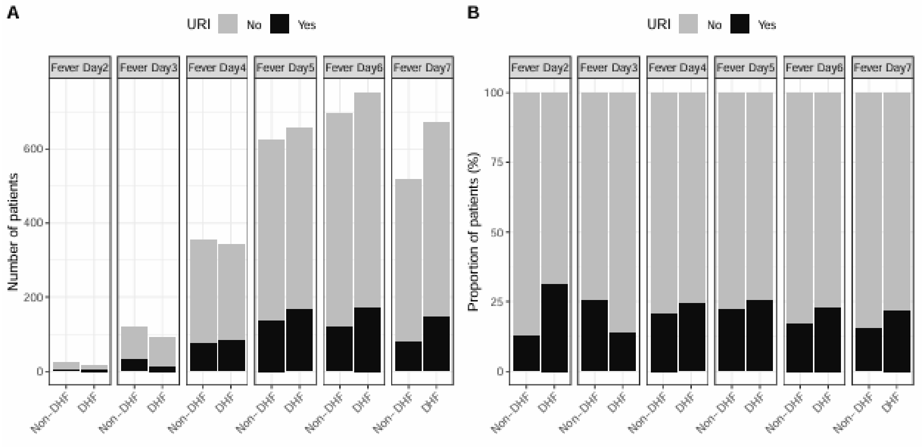

Fig. S10-11: Time course of `URI` from two to seven days after fever onset in two groups (DHF and Non-DHF). The data are shown as raw counts (A) and proportions by group by study day (B).
