# Supplementary material for: Prognostic prediction of dengue hemorrhagic fever in pediatric patients with suspected dengue infection: A multi-site study
Source: PLoS One. 2025 Aug 4;20(8):e0327360. doi: 10.1371/journal.pone.0327360 (PMC12321061; doi:10.1371/journal.pone.0327360)
Supplement: S11 File — (PDF) [file pone.0327360.s011.pdf]

Supplement file 11

Table S11-1: Numeric variables from symptoms and biological parameters with medians (inter-quartile ranges) and odds ratios for DHF and Non-DHF categories for Study day 1 data.

Description	Unit	Non-DHF	DHF	Odds Ratio
**Daily blood pressure**				
Systolic	mmHg	100 (92, 108)	100 (94, 106)	1.00 (0.99, 1.01)
Diastolic	mmHg	60 (59, 67)	60 (59, 70)	1.01 (1.00, 1.02)
**Daily pulse pressure**				
Minimum	mmHg	36 (30, 40)	35 (30, 40)	0.98 (0.97, 1.00)
**Daily fingertip hematocrit**				
Minimum	%	37 (35, 40)	38 (35, 40)	1.07 (1.05, 1.10)
Maximum	%	39 (37, 41)	40 (38, 43)	1.12 (1.09, 1.15)
Average	%	38 (36, 40)	40 (37, 42)	1.11 (1.08, 1.14)
Range	%	1 (0, 3)	2 (0, 4)	1.14 (1.09, 1.19)
**Fluid intake and output**				
Difference between fluid intake and output	ml	200 (0, 550)	200 (0, 600)	1.00 (1.00, 1.00)
Daily maximum difference between fluid intake and output	ml	200 (0, 430)	200 (0, 500)	1.00 (1.00, 1.00)
**Daily pulse rate**				
Minimum	beats/minute	92 (84, 100)	92 (84, 100)	1.00 (0.99, 1.00)
Maximum	beats/minute	110 (100, 120)	110 (100, 120)	1.00 (0.99, 1.00)
Average	beats/minute	101 (94, 110)	100 (93, 109)	1.00 (0.99, 1.00)
Range	beats/minute	14 (8, 22)	16 (8, 24)	1.00 (0.99, 1.01)
**Daily body temperature**				
Minimum	°C	37.5 (37.0, 38.1)	37.7 (37.0, 38.4)	1.19 (1.07, 1.33)
Maximum	°C	39.2 (38.7, 39.7)	39.3 (38.8, 39.9)	1.16 (1.03, 1.31)
Average	°C	38.4 (37.9, 38.9)	38.5 (38.0, 39.0)	1.27 (1.11, 1.45)
Range	°C	1.6 (0.9, 2.3)	1.6 (0.8, 2.3)	0.95 (0.86, 1.05)
Abdominal circumference	cm	55.0 (50.0, 62.0)	58.8 (52.0, 64.0)	1.02 (1.01, 1.03)
Liver size	cm	0.0 (0.0, 0.0)	0.0 (0.0, 0.0)	1.35 (1.16, 1.57)
Body weight	kg	27.5 (20.6, 39.3)	31.1 (22.3, 43.0)	1.01 (1.01, 1.02)
Venipuncture hematocrit	%	37.0 (35.0, 39.0)	38.0 (36.0, 40.7)	1.09 (1.07, 1.12)
Atypical lymphocyte count*	x1000 cells/mm3	0.00 (0.00, 0.110)	0.00 (0.00, 0.128)	3.03 (0.20, 46.75)

Continued on next page

Description	Unit	Non-DHF	DHF	Odds Ratio
Band cell count*	x1000 cells/mm3	0.00 (0.00, 0.165)	0.00 (0.00, 0.128)	123.04 (3.35, 4678.55)
Basophil count*	x1000 cells/mm3	0.00 (0.00, 0.096)	0.00 (0.00, 0.100)	0.37 (0.00, 35.28)
Eosinophil count*	x1000 cells/mm3	0.00 (0.00, 0.127)	0.00 (0.00, 0.128)	0.05 (0.00, 2.44)
Lymphocyte count*	x1000 cells/mm3	0.106 (0.00, 0.163)	0.102 (0.00, 0.169)	0.00 (0.00, 0.00)
Monocyte count*	x1000 cells/mm3	0.082 (0.00, 0.130)	0.079 (0.00, 0.136)	0.00 (0.00, 0.05)
Polymorphonuclear Leukocyte count*	x1000 cells/mm3	0.114 (0.068, 0.190)	0.114 (0.00, 0.186)	0.02 (0.00, 4.76)
White blood cell count*	x1000 cells/mm3	3.5 (3.4, 3.7)	3.5 (3.4, 3.6)	0.55 (0.34, 0.88)
Albumin	g/dL	3.7 (3.5, 3.9)	3.5 (3.2, 3.8)	0.33 (0.26, 0.43)
ALT	IU/L	36 (25, 52)	49 (35, 79)	1.01 (1.01, 1.01)
AST	IU/L	58 (42, 90)	96 (60, 167)	1.01 (1.01, 1.01)
Total protein	g/dL	6.8 (6.4, 7.2)	6.6 (6.1, 7.0)	0.52 (0.44, 0.60)
Platelet count	x1000 cells/mm3	152 (115, 196)	109 (72, 150)	0.99 (0.99, 0.99)
Albumin:Globulin ratio	-	0.538 (0.508, 0.571)	0.532 (0.506, 0.563)	0.06 (0.01, 0.44)
AST:Platelet ratio	(IU/L)/(x1000 cells/mm3)	0.384 (0.226, 0.740)	0.980 (0.458, 2.466)	1.62 (1.48, 1.79)
AST:ALT ratio	-	1.697 (1.235, 2.379)	1.963 (1.480, 2.538)	1.25 (1.13, 1.38)
Day of illness	day	3 (2, 4)	3 (2, 4)	0.99 (0.91, 1.08)

Note: AST: Aspartate Transaminase, ALT: Alanine Transaminase.

*Data are presented as median (min, max).

Table S11-2: Numeric variables from symptoms and biological parameters with medians (inter-quartile ranges) and odds ratios for DHF and Non-DHF categories for Study day 1 data.

Description	Unit	Non-DHF	DHF	Odds Ratio
**Daily blood pressure**				
Systolic	mmHg	100 (92, 108)	100 (94, 106)	1.00 (0.99, 1.01)
Diastolic	mmHg	60 (59, 67)	60 (59, 70)	1.01 (1.00, 1.02)
**Daily pulse pressure**				
Minimum	mmHg	36 (30, 40)	35 (30, 40)	0.98 (0.97, 1.00)
**Daily fingertip hematocrit**				
Minimum	%	37 (35, 40)	38 (35, 40)	1.07 (1.05, 1.10)
Maximum	%	39 (37, 41)	40 (38, 43)	1.12 (1.09, 1.15)
Average	%	38 (36, 40)	40 (37, 42)	1.11 (1.08, 1.14)
Range	%	1 (0, 3)	2 (0, 4)	1.14 (1.09, 1.19)
**Fluid intake and output**				
Difference between fluid intake and output	ml	200 (0, 550)	200 (0, 600)	1.00 (1.00, 1.00)

Continued on next page

Description	Unit	Non-DHF	DHF	Odds Ratio
Daily maximum difference between fluid intake and output	ml	200 (0, 430)	200 (0, 500)	1.00 (1.00, 1.00)
**Daily pulse rate**				
Minimum	beats/minute	92 (84, 100)	92 (84, 100)	1.00 (0.99, 1.00)
Maximum	beats/minute	110 (100, 120)	110 (100, 120)	1.00 (0.99, 1.00)
Average	beats/minute	101 (94, 110)	100 (93, 109)	1.00 (0.99, 1.00)
Range	beats/minute	14 (8, 22)	16 (8, 24)	1.00 (0.99, 1.01)
**Daily body temperature**				
Minimum	˚C	37.5 (37.0, 38.1)	37.7 (37.0, 38.4)	1.19 (1.07, 1.33)
Maximum	˚C	39.2 (38.7, 39.7)	39.3 (38.8, 39.9)	1.16 (1.03, 1.31)
Average	˚C	38.4 (37.9, 38.9)	38.5 (38.0, 39.0)	1.27 (1.11, 1.45)
Range	˚C	1.6 (0.9, 2.3)	1.6 (0.8, 2.3)	0.95 (0.86, 1.05)
Abdominal circumference	cm	55.0 (50.0, 62.0)	58.8 (52.0, 64.0)	1.02 (1.01, 1.03)
Liver size	cm	0.0 (0.0, 0.0)	0.0 (0.0, 0.0)	1.35 (1.16, 1.57)
Body weight	kg	27.5 (20.6, 39.3)	31.1 (22.3, 43.0)	1.01 (1.01, 1.02)
Venipuncture hematocrit	%	37.0 (35.0, 39.0)	38.0 (36.0, 40.7)	1.09 (1.07, 1.12)
Atypical lymphocyte count*	x1000 cells/mm3	0.00 (0.00, 0.110)	0.00 (0.00, 0.128)	3.03 (0.20, 46.75)
Band cell count*	x1000 cells/mm3	0.00 (0.00, 0.165)	0.00 (0.00, 0.128)	123.04 (3.35, 4678.55)
Basophil count*	x1000 cells/mm3	0.00 (0.00, 0.096)	0.00 (0.00, 0.100)	0.37 (0.00, 35.28)
Eosinophil count*	x1000 cells/mm3	0.00 (0.00, 0.127)	0.00 (0.00, 0.128)	0.05 (0.00, 2.44)
Lymphocyte count*	x1000 cells/mm3	0.106 (0.00, 0.163)	0.102 (0.00, 0.169)	0.00 (0.00, 0.00)
Monocyte count*	x1000 cells/mm3	0.082 (0.00, 0.130)	0.079 (0.00, 0.136)	0.00 (0.00, 0.05)
Polymorphonuclear Leukocyte count*	x1000 cells/mm3	0.114 (0.068, 0.190)	0.114 (0.00, 0.186)	0.02 (0.00, 4.76)
White blood cell count*	x1000 cells/mm3	3.5 (3.4, 3.7)	3.5 (3.4, 3.6)	0.55 (0.34, 0.88)
Albumin	g/dL	3.7 (3.5, 3.9)	3.5 (3.2, 3.8)	0.33 (0.26, 0.43)
ALT	IU/L	36 (25, 52)	49 (35, 79)	1.01 (1.01, 1.01)
AST	IU/L	58 (42, 90)	96 (60, 167)	1.01 (1.01, 1.01)
Total protein	g/dL	6.8 (6.4, 7.2)	6.6 (6.1, 7.0)	0.52 (0.44, 0.60)
Platelet count	x1000 cells/mm3	152 (115, 196)	109 (72, 150)	0.99 (0.99, 0.99)
Albumin:Globulin ratio	-	0.538 (0.508, 0.571)	0.532 (0.506, 0.563)	0.06 (0.01, 0.44)
AST:Platelet ratio	(IU/L)/(x1000 cells/mm3)	0.384 (0.226, 0.740)	0.980 (0.458, 2.466)	1.62 (1.48, 1.79)
AST:ALT ratio	-	1.697 (1.235, 2.379)	1.963 (1.480, 2.538)	1.25 (1.13, 1.38)
Day of illness	day	3 (2, 4)	3 (2, 4)	0.99 (0.91, 1.08)

Continued on next page

Description	Unit	Non-DHF	DHF	Odds Ratio
Note: AST: Aspartate Transaminase, ALT: Alanine Transaminase.				
*Data are presented as median (min, max).				

Table S11-3: Numeric variables from symptoms and biological parameters with medians (inter-quartile ranges) and odds ratios for DHF and Non-DHF categories for Study day 3 data.

Description	Unit	Non-DHF	DHF	Odds Ratio
**Daily blood pressure**				
Systolic	mmHg	94 (90, 100)	93 (90, 100)	1.00 (0.98, 1.01)
Diastolic	mmHg	60 (54, 60)	60 (55, 60)	1.02 (1.00, 1.04)
**Daily pulse pressure**				
Minimum	mmHg	30 (30, 36)	30 (30, 34)	0.95 (0.93, 0.98)
**Daily fingertip hematocrit**				
Minimum	%	37 (35, 39)	38 (35, 40)	1.11 (1.08, 1.15)
Maximum	%	39 (37, 41)	42 (39, 45)	1.23 (1.18, 1.27)
Average	%	38 (36, 40)	40 (38, 42)	1.20 (1.15, 1.24)
Range	%	2 (0, 3)	3 (2, 5)	1.38 (1.30, 1.47)
**Fluid intake and output**				
Difference between fluid intake and output	ml	285 (0, 775)	690 (0, 1400)	1.00 (1.00, 1.00)
Daily maximum difference between fluid intake and output	ml	300 (90, 500)	440 (150, 700)	1.00 (1.00, 1.00)
**Daily pulse rate**				
Minimum	beats/minute	83 (78, 90)	80 (76, 90)	0.99 (0.98, 1.00)
Maximum	beats/minute	102 (96, 112)	104 (96, 112)	1.00 (0.99, 1.01)
Average	beats/minute	94 (87, 100)	93 (86, 101)	1.00 (0.98, 1.01)
Range	beats/minute	20 (14, 26)	21 (16, 28)	1.02 (1.01, 1.04)
**Daily body temperature**				
Minimum	°C	36.7 (36.3, 37.0)	36.7 (36.3, 37.2)	1.23 (1.01, 1.50)
Maximum	°C	38.5 (37.8, 39.2)	38.8 (37.9, 39.5)	1.17 (1.04, 1.33)
Average	°C	37.5 (37.1, 38.1)	37.8 (37.2, 38.3)	1.26 (1.07, 1.50)
Range	°C	1.8 (1.2, 2.3)	1.8 (1.2, 2.5)	1.13 (0.97, 1.31)
Abdominal circumference	cm	56.0 (50.0, 63.0)	58.0 (52.0, 64.1)	1.02 (1.00, 1.03)
Liver size	cm	0.5 (0.0, 1.0)	1.0 (0.0, 2.0)	1.69 (1.51, 1.90)
Body weight	kg	27.7 (21.4, 40.0)	31.6 (22.9, 42.8)	1.01 (1.00, 1.02)
Venipuncture hematocrit	%	36.0 (34.0, 38.9)	38.0 (36.0, 41.0)	1.15 (1.11, 1.19)

Continued on next page

Description	Unit	Non-DHF	DHF	Odds Ratio
Atypical lymphocyte count*	x1000 cells/mm3	0.066 (0.00, 0.134)	0.072 (0.00, 0.163)	470.39 (23.78, 9578.98)
Band cell count*	x1000 cells/mm3	0.00 (0.00, 0.122)	0.00 (0.00, 0.124)	231.87 (2.83, 20084.22)
Basophil count*	x1000 cells/mm3	0.00 (0.00, 0.088)	0.00 (0.00, 0.082)	0.05 (0.00, 28.19)
Eosinophil count*	x1000 cells/mm3	0.00 (0.00, 0.121)	0.00 (0.00, 0.115)	0.00 (0.00, 0.03)
Lymphocyte count*	x1000 cells/mm3	0.111 (0.00, 0.154)	0.107 (0.062, 0.195)	0.00 (0.00, 0.00)
Monocyte count*	x1000 cells/mm3	0.079 (0.00, 0.133)	0.075 (0.00, 0.153)	0.00 (0.00, 0.53)
Polymorphonuclear Leukocyte count*	x1000 cells/mm3	0.105 (0.064, 0.162)	0.104 (0.066, 0.187)	0.00 (0.00, 8.04)
White blood cell count*	x1000 cells/mm3	3.5 (3.4, 3.6)	3.4 (3.3, 3.6)	0.38 (0.21, 0.67)
Albumin	g/dL	3.5 (3.2, 3.7)	3.2 (2.9, 3.5)	0.15 (0.11, 0.22)
ALT	IU/L	39 (27, 57)	53 (37, 84)	1.01 (1.00, 1.01)
AST	IU/L	65 (43, 102)	108 (74, 182)	1.01 (1.00, 1.01)
Total protein	g/dL	6.6 (6.2, 6.9)	6.2 (5.7, 6.7)	0.41 (0.33, 0.49)
Platelet count	x1000 cells/mm3	114 (77, 160)	55 (32, 85)	0.98 (0.98, 0.98)
Albumin:Globulin ratio	-	0.524 (0.500, 0.561)	0.516 (0.483, 0.554)	0.03 (0.00, 0.22)
AST:Platelet ratio	(IU/L)/(x1000 cells/mm3)	0.607 (0.292, 1.291)	2.352 (1.035, 4.942)	1.43 (1.33, 1.54)
AST:ALT ratio	-	1.700 (1.291, 2.333)	2.029 (1.581, 2.755)	1.40 (1.23, 1.61)
Day of illness	day	5 (4, 5)	5 (4, 5)	0.93 (0.83, 1.03)

Note: AST: Aspartate Transaminase, ALT: Alanine Transaminase.

*Data are presented as median (min, max).

Table S11-4: Numeric variables from symptoms and biological parameters with medians (inter-quartile ranges) and odds ratios for DHF and Non-DHF categories for Study day 4 data.

Description	Unit	Non-DHF	DHF	Odds Ratio
**Daily blood pressure**				
Systolic	mmHg	93 (90, 100)	92 (90, 100)	1.00 (0.97, 1.02)
Diastolic	mmHg	60 (53, 60)	60 (55, 60)	1.03 (1.00, 1.07)
**Daily pulse pressure**				
Minimum	mmHg	30 (30, 36)	30 (30, 33)	0.93 (0.89, 0.96)
**Daily fingertip hematocrit**				
Minimum	%	37 (34, 38)	38 (36, 41)	1.15 (1.10, 1.22)
Maximum	%	38 (36, 40)	42 (39, 45)	1.25 (1.19, 1.32)
Average	%	38 (35, 40)	40 (38, 43)	1.23 (1.16, 1.30)
Range	%	2 (0, 3)	3 (2, 5)	1.46 (1.33, 1.61)
**Fluid intake and output**				

Continued on next page

Description	Unit	Non-DHF	DHF	Odds Ratio
Difference between fluid intake and output	ml	152 (0, 600)	780 (100, 1394)	1.00 (1.00, 1.00)
Daily maximum difference between fluid intake and output	ml	220 (28, 400)	500 (200, 700)	1.00 (1.00, 1.00)
**Daily pulse rate**				
Minimum	beats/minute	84 (80, 90)	80 (76, 90)	0.98 (0.96, 0.99)
Maximum	beats/minute	104 (96, 112)	103 (96, 112)	1.00 (0.98, 1.01)
Average	beats/minute	94 (88, 102)	93 (86, 101)	0.99 (0.97, 1.00)
Range	beats/minute	20 (14, 26)	20 (16, 28)	1.02 (1.00, 1.04)
**Daily body temperature**				
Minimum	°C	36.6 (36.3, 37.0)	36.8 (36.3, 37.1)	1.30 (0.96, 1.78)
Maximum	°C	38.4 (37.8, 39.2)	38.7 (38.0, 39.5)	1.20 (0.99, 1.45)
Average	°C	37.5 (37.1, 38.0)	37.7 (37.2, 38.2)	1.34 (1.03, 1.76)
Range	°C	1.7 (1.2, 2.3)	1.8 (1.2, 2.4)	1.09 (0.87, 1.36)
Abdominal circumference	cm	54.4 (50.0, 63.2)	59.0 (54.0, 65.0)	1.02 (1.01, 1.04)
Liver size	cm	0.5 (0.0, 1.1)	1.5 (0.5, 2.0)	1.76 (1.49, 2.09)
Body weight	kg	26.0 (20.0, 38.8)	33.6 (24.4, 43.9)	1.02 (1.01, 1.03)
Venipuncture hematocrit	%	36.0 (34.0, 38.2)	38.2 (36.0, 41.0)	1.17 (1.11, 1.23)
Atypical lymphocyte count*	x1000 cells/mm3	0.067 (0.00, 0.158)	0.075 (0.00, 0.139)	1231.91 (10.48, 159823.79)
Band cell count*	x1000 cells/mm3	0.00 (0.00, 0.123)	0.00 (0.00, 0.098)	6.42 (0.01, 6013.63)
Basophil count*	x1000 cells/mm3	0.00 (0.00, 0.084)	0.00 (0.00, 0.075)	8.06 (0.00, 242596.15)
Eosinophil count*	x1000 cells/mm3	0.00 (0.00, 0.107)	0.00 (0.00, 0.091)	0.00 (0.00, 0.75)
Lymphocyte count*	x1000 cells/mm3	0.110 (0.00, 0.168)	0.105 (0.00, 0.155)	0.00 (0.00, 0.02)
Monocyte count*	x1000 cells/mm3	0.077 (0.00, 0.163)	0.073 (0.00, 0.121)	0.01 (0.00, 17.29)
Polymorphonuclear Leukocyte count*	x1000 cells/mm3	0.104 (0.068, 0.160)	0.104 (0.064, 0.149)	0.00 (0.00, 25.27)
White blood cell count*	x1000 cells/mm3	3.5 (3.4, 3.7)	3.4 (3.3, 3.6)	0.35 (0.15, 0.79)
Albumin	g/dL	3.4 (3.2, 3.6)	3.1 (2.7, 3.4)	0.16 (0.09, 0.25)
ALT	IU/L	43 (28, 66)	54 (38, 95)	1.01 (1.00, 1.01)
AST	IU/L	76 (46, 118)	116 (78, 206)	1.00 (1.00, 1.01)
Total protein	g/dL	6.6 (6.2, 6.9)	6.1 (5.5, 6.6)	0.41 (0.31, 0.54)
Platelet count	x1000 cells/mm3	115 (80, 166)	45 (28, 81)	0.97 (0.97, 0.98)
Albumin:Globulin ratio	-	0.522 (0.493, 0.562)	0.516 (0.484, 0.551)	0.05 (0.00, 1.19)

Continued on next page

Description	Unit	Non-DHF	DHF	Odds Ratio
AST:Platelet ratio	(IU/L)/(x1000 cells/mm3)	0.692 (0.280, 1.459)	2.783 (1.393, 6.534)	1.28 (1.20, 1.39)
AST:ALT ratio	-	1.784 (1.250, 2.442)	2.057 (1.689, 2.806)	1.45 (1.19, 1.78)
Day of illness	day	5 (4, 6)	5 (4, 6)	0.87 (0.74, 1.02)

Note: AST: Aspartate Transaminase, ALT: Alanine Transaminase.

*Data are presented as median (min, max).

Table S11-5: Numeric variables from symptoms and biological parameters with medians (inter-quartile ranges) and odds ratios for DHF and Non-DHF categories for Study day 5 data.

Description	Unit	Non-DHF	DHF	Odds Ratio
**Minimum**				
Maximum	°C	38.4 (37.7, 39.0)	38.0 (37.5, 39.4)	1.02 (0.75, 1.40)
Average	°C	37.5 (37.1, 37.9)	37.5 (36.9, 38.0)	0.97 (0.62, 1.53)
**Range**				
Abdominal circumference	cm	55.0 (50.0, 64.0)	57.5 (51.0, 62.0)	1.00 (0.97, 1.03)
**Liver size**				
Body weight	kg	25.0 (20.4, 39.5)	30.0 (22.7, 38.2)	1.00 (0.98, 1.03)
Venipuncture hematocrit	%	35.5 (33.0, 38.0)	39.0 (35.0, 41.0)	1.16 (1.07, 1.26)
Atypical lymphocyte count*	x1000 cells/mm3	0.064 (0.00, 0.120)	0.088 (0.00, 0.125)	1.605510e+09 (1.429246e+05, 5.783720e+13)
Band cell count*	x1000 cells/mm3	0.00 (0.00, 0.101)	0.00 (0.00, 0.114)	0.01 (0.00, 999.39)
**Basophil count***				
Eosinophil count*	x1000 cells/mm3	0.00 (0.00, 0.096)	0.00 (0.00, 0.103)	0.00 (0.00, 4.50)
Lymphocyte count*	x1000 cells/mm3	0.113 (0.00, 0.142)	0.113 (0.085, 0.162)	0.37 (0.00, 49568271.73)
**Monocyte count***				
Polymorphonuclear Leukocyte count*	x1000 cells/mm3	0.107 (0.071, 0.159)	0.105 (0.071, 0.148)	0.00 (0.00, 8765.33)
White blood cell count*	x1000 cells/mm3	3.5 (3.4, 3.7)	3.5 (3.4, 3.6)	0.65 (0.15, 2.70)
Albumin	g/dL	3.4 (3.1, 3.6)	3.1 (2.9, 3.4)	0.24 (0.11, 0.49)
ALT	IU/L	53 (33, 96)	61 (42, 118)	1.00 (1.00, 1.01)
**AST**				
Total protein	g/dL	6.5 (6.1, 6.9)	6.0 (5.2, 6.4)	0.44 (0.28, 0.66)
Platelet count	x1000 cells/mm3	103 (74, 164)	50 (32, 70)	0.97 (0.96, 0.98)
Albumin:Globulin ratio	-	0.514 (0.486, 0.550)	0.519 (0.485, 0.562)	3.02 (0.03, 487.24)

Continued on next page

Description	Unit	Non-DHF	DHF	Odds Ratio
AST:Platelet ratio	(IU/L)/(x1000 cells/mm3)	0.920 (0.349, 2.439)	3.243 (1.789, 5.882)	1.40 (1.22, 1.63)
AST:ALT ratio	-	1.781 (1.217, 2.284)	2.067 (1.635, 2.689)	1.54 (1.09, 2.21)
Day of illness	day	6 (5, 6)	5 (5, 6)	0.68 (0.50, 0.91)
Body weight	kg	27.7 (21.4, 40.0)	31.6 (22.9, 42.8)	1.01 (1.00, 1.02)
Venipuncture hematocrit	%	36.0 (34.0, 38.9)	38.0 (36.0, 41.0)	1.15 (1.11, 1.19)
Atypical lymphocyte count*	x1000 cells/mm3	0.066 (0.00, 0.134)	0.072 (0.00, 0.163)	470.39 (23.78, 9578.98)
Band cell count*	x1000 cells/mm3	0.00 (0.00, 0.122)	0.00 (0.00, 0.124)	231.87 (2.83, 20084.22)
Basophil count*	x1000 cells/mm3	0.00 (0.00, 0.088)	0.00 (0.00, 0.082)	0.05 (0.00, 28.19)
Eosinophil count*	x1000 cells/mm3	0.00 (0.00, 0.121)	0.00 (0.00, 0.115)	0.00 (0.00, 0.03)
Lymphocyte count*	x1000 cells/mm3	0.111 (0.00, 0.154)	0.107 (0.062, 0.195)	0.00 (0.00, 0.00)
Monocyte count*	x1000 cells/mm3	0.079 (0.00, 0.133)	0.075 (0.00, 0.153)	0.00 (0.00, 0.53)
Polymorphonuclear Leukocyte count*	x1000 cells/mm3	0.105 (0.064, 0.162)	0.104 (0.066, 0.187)	0.00 (0.00, 8.04)
White blood cell count*	x1000 cells/mm3	3.5 (3.4, 3.6)	3.4 (3.3, 3.6)	0.38 (0.21, 0.67)
Albumin	g/dL	3.5 (3.2, 3.7)	3.2 (2.9, 3.5)	0.15 (0.11, 0.22)
ALT	IU/L	39 (27, 57)	53 (37, 84)	1.01 (1.00, 1.01)
AST	IU/L	65 (43, 102)	108 (74, 182)	1.01 (1.00, 1.01)
Total protein	g/dL	6.6 (6.2, 6.9)	6.2 (5.7, 6.7)	0.41 (0.33, 0.49)
Platelet count	x1000 cells/mm3	114 (77, 160)	55 (32, 85)	0.98 (0.98, 0.98)
Albumin:Globulin ratio	-	0.524 (0.500, 0.561)	0.516 (0.483, 0.554)	0.03 (0.00, 0.22)
AST:Platelet ratio	(IU/L)/(x1000 cells/mm3)	0.607 (0.292, 1.291)	2.352 (1.035, 4.942)	1.43 (1.33, 1.54)
AST:ALT ratio	-	1.700 (1.291, 2.333)	2.029 (1.581, 2.755)	1.40 (1.23, 1.61)
Day of illness	day	5 (4, 5)	5 (4, 5)	0.93 (0.83, 1.03)

Note: AST: Aspartate Transaminase, ALT: Alanine Transaminase.

*Data are presented as median (min, max).
