# Supplementary material for: Prognostic prediction of dengue hemorrhagic fever in pediatric patients with suspected dengue infection: A multi-site study
Source: PLoS One. 2025 Aug 4;20(8):e0327360. doi: 10.1371/journal.pone.0327360 (PMC12321061; doi:10.1371/journal.pone.0327360)
Supplement: S12 File — (PDF) [file pone.0327360.s012.pdf]

Supplement file 12

Table S12-1: Numeric variables from symptoms and biological parameters with medians (inter-quartile ranges) and odds ratios for DSS and Non-DSS categories.

Description	Unit	Non-DHF	DHF	Odds Ratio
**Daily blood pressure**				
Systolic	mmHg	98 (90, 100)	90 (90, 100)	0.94 (0.93, 0.96)
Diastolic	mmHg	60 (56, 60)	60 (58, 60)	0.99 (0.97, 1.02)
**Daily pulse pressure**				
Minimum	mmHg	30 (30, 40)	30 (21, 30)	0.82 (0.80, 0.85)
**Daily fingertip hematocrit**				
Minimum	%	37 (35, 40)	38 (35, 40)	1.01 (0.97, 1.05)
Maximum	%	40 (37, 42)	42 (39, 46)	1.13 (1.09, 1.16)
Average	%	38 (36, 41)	40 (37, 42)	1.08 (1.04, 1.12)
Range	%	2 (0, 4)	4 (2, 7)	1.29 (1.23, 1.34)
**Fluid intake and output**				
Difference between fluid intake and output	ml	340 (0, 840)	250 (0, 1288)	1.00 (1.00, 1.00)
Daily maximum difference between fluid intake and output	ml	300 (0, 550)	275 (0, 700)	1.00 (1.00, 1.00)
**Daily pulse rate**				
Minimum	beats/minute	86 (80, 96)	84 (80, 94)	0.99 (0.97, 1.00)
Maximum	beats/minute	108 (98, 116)	108 (100, 120)	1.02 (1.01, 1.03)
Average	beats/minute	97 (90, 105)	96 (90, 105)	1.00 (0.99, 1.02)
Range	beats/minute	20 (12, 26)	24 (12, 34)	1.04 (1.03, 1.05)
**Daily body temperature**				
Minimum	°C	37.0 (36.5, 37.6)	37.0 (36.5, 37.7)	1.03 (0.87, 1.20)
Maximum	°C	39.0 (38.3, 39.7)	39.0 (38.3, 39.8)	1.02 (0.88, 1.18)
Average	°C	38.0 (37.5, 38.5)	38.1 (37.5, 38.6)	1.03 (0.86, 1.23)
Range	°C	1.8 (1.2, 2.4)	1.8 (1.2, 2.6)	1.00 (0.85, 1.16)
Abdominal circumference	cm	57.0 (51.0, 64.0)	56.0 (52.0, 61.0)	0.99 (0.97, 1.00)
Liver size	cm	0.0 (0.0, 1.0)	1.0 (0.0, 2.0)	1.38 (1.25, 1.52)
Body weight	kg	29.7 (21.9, 41.5)	26.0 (21.1, 35.8)	0.98 (0.97, 0.99)
Venipuncture hematocrit	%	37.0 (35.0, 39.5)	38.0 (35.0, 40.9)	1.03 (0.99, 1.06)
Atypical lymphocyte count*	x1000 cells/mm3	0.056 (0.000, 0.163)	0.063 (0.000, 0.140)	23.80 (0.79, 733.09)
Band cell count*	x1000 cells/mm3	0.000 (0.000, 0.165)	0.000 (0.000, 0.118)	32.60 (0.26, 2913.29)

Continued on next page

Description	Unit	Non-DHF	DHF	Odds Ratio
Basophil count*	x1000 cells/mm3	0.000 (0.000, 0.100)	0.000 (0.000, 0.085)	65.84 (0.07, 37098.57)
Eosinophil count*	x1000 cells/mm3	0.000 (0.000, 0.131)	0.000 (0.000, 0.091)	0.04 (0.00, 6.42)
Lymphocyte count*	x1000 cells/mm3	0.106 (0.000, 0.195)	0.105 (0.069, 0.169)	0.03 (0.00, 286.38)
Monocyte count*	x1000 cells/mm3	0.078 (0.000, 0.163)	0.075 (0.000, 0.136)	0.63 (0.00, 186.32)
Polymorphonuclear Leukocyte count*	x1000 cells/mm3	0.108 (0.000, 0.190)	0.110 (0.077, 0.178)	3239.13 (1.08, 8382123.61)
White blood cell count*	x1000 cells/mm3	3.5 (3.4, 3.6)	3.5 (3.4, 3.6)	1.20 (0.62, 2.28)
Albumin	g/dL	3.5 (3.2, 3.7)	3.1 (2.7, 3.5)	0.21 (0.16, 0.28)
ALT	IU/L	43 (30, 68)	59 (43, 100)	1.00 (1.00, 1.00)
AST	IU/L	77 (49, 129)	131 (76, 210)	1.00 (1.00, 1.00)
Total protein	g/dL	6.6 (6.1, 7.0)	6.2 (5.3, 6.7)	0.50 (0.43, 0.58)
Platelet count	x1000 cells/mm3	107 (66, 158)	67 (30, 113)	0.99 (0.99, 0.99)
Albumin:Globulin ratio	-	0.529 (0.500, 0.562)	0.524 (0.481, 0.554)	0.01 (0.00, 0.05)
AST:Platelet ratio	(IU/L)/(x1000 cells/mm3)	0.764 (0.346, 2.052)	2.370 (0.727, 6.792)	1.06 (1.04, 1.08)
AST:ALT ratio	-	1.854 (1.358, 2.493)	2.064 (1.631, 2.772)	1.14 (1.04, 1.26)
Day of illness	day	4 (3, 5)	4 (3, 5)	0.99 (0.90, 1.09)

Note: AST: Aspartate Transaminase, ALT: Alanine Transaminase.

*Data are presented as median (min, max).

Table S12-2: Numeric variables from symptoms and biological parameters with medians (inter-quartile ranges) and odds ratios for DSS and Non-DSS categories for Study day 1 data.

Description	Unit	Non-DHF	DHF	Odds Ratio
**Daily blood pressure**				
Systolic	mmHg	100 (93, 107)	100 (90, 100)	0.98 (0.96, 1.01)
Diastolic	mmHg	60 (59, 69)	60 (60, 62)	1.00 (0.97, 1.03)
**Daily pulse pressure**				
Minimum	mmHg	36 (30, 40)	30 (30, 40)	0.89 (0.85, 0.93)
**Daily fingertip hematocrit**				
Minimum	%	38 (35, 40)	38 (35, 40)	1.01 (0.95, 1.07)
Maximum	%	40 (37, 42)	41 (38, 45)	1.10 (1.04, 1.17)
Average	%	38 (36, 41)	40 (36, 42)	1.06 (1.00, 1.13)
Range	%	1 (0, 3)	3 (0, 5)	1.20 (1.12, 1.29)
**Fluid intake and output**				
Difference between fluid intake and output	ml	200 (0, 600)	0 (0, 425)	1.00 (1.00, 1.00)

Continued on next page

Description	Unit	Non-DHF	DHF	Odds Ratio
Daily maximum difference between fluid intake and output	ml	200 (0, 470)	0 (0, 322)	1.00 (1.00, 1.00)
**Daily pulse rate**				
Minimum	beats/minute	92 (84, 100)	90 (80, 100)	0.98 (0.96, 1.00)
Maximum	beats/minute	110 (100, 120)	108 (97, 120)	1.00 (0.98, 1.02)
Average	beats/minute	101 (93, 110)	99 (91, 108)	0.99 (0.97, 1.01)
Range	beats/minute	14 (8, 22)	16 (6, 30)	1.02 (1.00, 1.04)
**Daily body temperature**				
Minimum	˚C	37.6 (37.0, 38.2)	37.5 (36.7, 38.0)	0.88 (0.67, 1.13)
Maximum	˚C	39.3 (38.7, 39.8)	39.2 (38.3, 39.8)	0.71 (0.55, 0.93)
Average	˚C	38.5 (38.0, 39.0)	38.4 (37.7, 38.8)	0.71 (0.51, 0.98)
Range	˚C	1.6 (0.9, 2.3)	1.4 (0.6, 2.2)	0.87 (0.69, 1.10)
Abdominal circumference	cm	56.5 (50.0, 63.0)	58.0 (52.0, 60.5)	0.99 (0.97, 1.01)
Liver size	cm	0.0 (0.0, 0.0)	0.0 (0.0, 1.0)	1.70 (1.37, 2.09)
Body weight	kg	30.0 (21.6, 41.5)	26.0 (21.0, 36.0)	0.98 (0.96, 1.00)
Venipuncture hematocrit	%	37.6 (35.0, 40.0)	37.0 (34.1, 40.9)	0.98 (0.92, 1.04)
Atypical lymphocyte count*	x1000 cells/mm3	0.000 (0.000, 0.128)	0.000 (0.000, 0.107)	846.17 (1.85, 346591.69)
Band cell count*	x1000 cells/mm3	0.000 (0.000, 0.165)	0.000 (0.000, 0.107)	394.82 (0.18, 397512.67)
Basophil count*	x1000 cells/mm3	0.000 (0.000, 0.100)	0.000 (0.000, 0.079)	94.69 (0.00, 1516072.85)
Eosinophil count*	x1000 cells/mm3	0.000 (0.000, 0.128)	0.000 (0.000, 0.075)	64.41 (0.01, 205414.50)
Lymphocyte count*	x1000 cells/mm3	0.104 (0.000, 0.163)	0.103 (0.075, 0.169)	7.51 (0.00, 359798169.60)
Monocyte count*	x1000 cells/mm3	0.081 (0.000, 0.133)	0.074 (0.000, 0.136)	0.00 (0.00, 12.54)
Polymorphonuclear Leukocyte count*	x1000 cells/mm3	0.114 (0.000, 0.190)	0.114 (0.087, 0.178)	26.11 (0.00, 7528452.19)
White blood cell count*	x1000 cells/mm3	3.5 (3.4, 3.6)	3.5 (3.4, 3.7)	1.45 (0.49, 4.17)
Albumin	g/dL	3.6 (3.4, 3.9)	3.3 (2.9, 3.7)	0.18 (0.11, 0.27)
ALT	IU/L	41 (29, 62)	58 (40, 102)	1.00 (1.00, 1.01)
AST	IU/L	71 (47, 118)	119 (69, 194)	1.00 (1.00, 1.00)
Total protein	g/dL	6.7 (6.4, 7.2)	6.3 (5.7, 6.8)	0.35 (0.26, 0.46)
Platelet count	x1000 cells/mm3	133 (94, 178)	81 (52, 123)	0.99 (0.98, 0.99)
Albumin:Globulin ratio	-	0.536 (0.507, 0.567)	0.527 (0.500, 0.551)	0.00 (0.00, 0.07)

Continued on next page

Description	Unit	Non-DHF	DHF	Odds Ratio
AST:Platelet ratio	(IU/L)/(x1000 cells/mm3)	0.550 (0.288, 1.233)	1.460 (0.582, 4.528)	1.09 (1.06, 1.14)
AST:ALT ratio	-	1.848 (1.325, 2.467)	1.958 (1.527, 2.587)	1.16 (0.95, 1.37)
Day of illness	day	3 (2, 4)	3 (2, 4)	1.20 (0.98, 1.46)

Note: AST: Aspartate Transaminase, ALT: Alanine Transaminase.

*Data are presented as median (min, max).

Table S12-3: Numeric variables from symptoms and biological parameters with medians (inter-quartile ranges) and odds ratios for DSS and Non-DSS categories for Study day 2 data.

Description	Unit	Non-DHF	DHF	Odds Ratio
**Daily blood pressure**				
Systolic	mmHg	96 (90, 100)	90 (84, 100)	0.89 (0.85, 0.92)
Diastolic	mmHg	60 (55, 60)	60 (51, 60)	0.97 (0.93, 1.01)
**Daily pulse pressure**				
Minimum	mmHg	30 (30, 37)	30 (20, 30)	0.82 (0.78, 0.86)
**Daily fingertip hematocrit**				
Minimum	%	37 (35, 40)	38 (35, 40)	0.98 (0.91, 1.05)
Maximum	%	40 (37, 42)	42 (39, 44)	1.12 (1.05, 1.19)
Average	%	38 (36, 41)	40 (37, 42)	1.07 (0.99, 1.15)
Range	%	2 (1, 4)	4 (2, 6)	1.31 (1.20, 1.43)
**Fluid intake and output**				
Difference between fluid intake and output	ml	500 (0, 1070)	550 (0, 1120)	1.00 (1.00, 1.00)
Daily maximum difference between fluid intake and output	ml	380 (90, 600)	380 (0, 650)	1.00 (1.00, 1.00)
**Daily pulse rate**				
Minimum	beats/minute	84 (80, 92)	80 (80, 90)	0.98 (0.96, 1.01)
Maximum	beats/minute	108 (100, 116)	112 (100, 120)	1.02 (1.00, 1.04)
Average	beats/minute	96 (90, 104)	98 (90, 103)	1.00 (0.98, 1.03)
Range	beats/minute	20 (16, 28)	26 (20, 34)	1.05 (1.02, 1.07)
**Daily body temperature**				
Minimum	°C	36.8 (36.5, 37.4)	36.9 (36.3, 37.4)	0.98 (0.67, 1.40)
Maximum	°C	39.2 (38.5, 39.7)	39.2 (38.6, 40.0)	1.08 (0.82, 1.43)
Average	°C	38.0 (37.5, 38.5)	38.1 (37.6, 38.5)	1.05 (0.74, 1.51)
Range	°C	2.1 (1.5, 2.6)	2.1 (1.5, 2.7)	1.11 (0.82, 1.51)
Abdominal circumference	cm	57.0 (51.0, 64.0)	56.0 (52.0, 61.8)	0.99 (0.97, 1.02)
Liver size	cm	0.0 (0.0, 1.0)	1.0 (0.0, 2.0)	1.46 (1.21, 1.74)

Continued on next page

Description	Unit	Non-DHF	DHF	Odds Ratio
Body weight	kg	29.8 (22.0, 41.5)	25.0 (22.0, 36.0)	0.98 (0.96, 1.00)
Venipuncture hematocrit	%	37.0 (34.0, 39.0)	38.0 (35.0, 41.0)	1.05 (0.98, 1.12)
Atypical lymphocyte count*	x1000 cells/mm3	0.058 (0.000, 0.130)	0.063 (0.000, 0.120)	36.15 (0.05, 28327.28)
Band cell count*	x1000 cells/mm3	0.000 (0.000, 0.133)	0.000 (0.000, 0.101)	12.93 (0.00, 45258.80)
Basophil count*	x1000 cells/mm3	0.000 (0.000, 0.087)	0.000 (0.000, 0.085)	511.04 (0.00, 56918730.44)
Eosinophil count*	x1000 cells/mm3	0.000 (0.000, 0.131)	0.000 (0.000, 0.091)	0.72 (0.00, 5423.49)
Lymphocyte count*	x1000 cells/mm3	0.106 (0.063, 0.160)	0.107 (0.069, 0.150)	1.17139e+03 (0.00000e+00, 1.14414e+11)
Monocyte count*	x1000 cells/mm3	0.075 (0.000, 0.144)	0.079 (0.000, 0.126)	105.19 (0.00, 10386800.92)
Polymorphonuclear Leukocyte count*	x1000 cells/mm3	0.105 (0.056, 0.176)	0.110 (0.077, 0.149)	2.328283e+08 (3.638000e+01, 1.059259e+15)
White blood cell count*	x1000 cells/mm3	3.4 (3.3, 3.6)	3.5 (3.4, 3.7)	3.15 (0.91, 10.77)
Albumin	g/dL	3.5 (3.2, 3.7)	3.1 (2.8, 3.5)	0.18 (0.10, 0.30)
ALT	IU/L	41 (30, 65)	55 (40, 98)	1.00 (1.00, 1.00)
AST	IU/L	74 (48, 122)	107 (66, 166)	1.00 (1.00, 1.00)
Total protein	g/dL	6.5 (6.2, 6.9)	6.2 (5.6, 6.8)	0.51 (0.38, 0.68)
Platelet count	x1000 cells/mm3	104 (65, 151)	69 (27, 116)	0.99 (0.98, 0.99)
Albumin:Globulin ratio	-	0.529 (0.500, 0.561)	0.527 (0.486, 0.554)	0.00 (0.00, 0.08)
AST:Platelet ratio	(IU/L)/(x1000 cells/mm3)	0.754 (0.354, 1.930)	1.786 (0.644, 6.879)	1.06 (1.03, 1.09)
AST:ALT ratio	-	1.849 (1.354, 2.478)	1.909 (1.527, 2.642)	1.10 (0.85, 1.36)
Day of illness	day	4 (3, 5)	4 (3, 5)	1.04 (0.83, 1.30)

Note: AST: Aspartate Transaminase, ALT: Alanine Transaminase.

*Data are presented as median (min, max).

Table S12-4: Numeric variables from symptoms and biological parameters with medians (inter-quartile ranges) and odds ratios for DSS and Non-DSS categories for Study day 3 data.

Description	Unit	Non-DHF	DHF	Odds Ratio
**Daily blood pressure**				
Systolic	mmHg	94 (90, 100)	90 (85, 94)	0.89 (0.84, 0.93)

Continued on next page

Description	Unit	Non-DHF	DHF	Odds Ratio
Diastolic	mmHg	60 (55, 60)	60 (52, 60)	0.95 (0.90, 1.00)
**Daily pulse pressure**				
Minimum	mmHg	30 (30, 35)	29 (20, 30)	0.70 (0.64, 0.77)
**Daily fingertip hematocrit**				
Minimum	%	37 (35, 40)	37 (35, 40)	0.99 (0.91, 1.08)
Maximum	%	40 (37, 43)	42 (40, 47)	1.12 (1.04, 1.21)
Average	%	38 (36, 41)	40 (38, 42)	1.07 (0.98, 1.16)
Range	%	3 (1, 4)	5 (3, 8)	1.34 (1.21, 1.48)
**Fluid intake and output**				
Difference between fluid intake and output	ml	430 (0, 1000)	785 (0, 1750)	1.00 (1.00, 1.00)
Daily maximum difference between fluid intake and output	ml	350 (100, 600)	475 (0, 776)	1.00 (1.00, 1.00)
**Daily pulse rate**				
Minimum	beats/minute	82 (78, 90)	81 (72, 92)	1.00 (0.96, 1.03)
Maximum	beats/minute	103 (96, 112)	106 (100, 112)	1.03 (1.00, 1.05)
Average	beats/minute	94 (87, 100)	94 (87, 102)	1.02 (0.98, 1.05)
Range	beats/minute	20 (14, 26)	24 (20, 32)	1.05 (1.01, 1.08)
**Daily body temperature**				
Minimum	°C	36.7 (36.3, 37.0)	36.8 (36.1, 37.3)	1.17 (0.70, 1.88)
Maximum	°C	38.6 (37.8, 39.4)	38.8 (38.0, 39.6)	1.18 (0.86, 1.64)
Average	°C	37.6 (37.1, 38.2)	37.7 (37.2, 38.4)	1.23 (0.80, 1.90)
Range	°C	1.8 (1.2, 2.4)	1.8 (1.4, 2.3)	1.16 (0.79, 1.71)
Abdominal circumference	cm	57.0 (51.0, 64.0)	54.5 (51.0, 61.1)	0.97 (0.94, 1.01)
Liver size	cm	0.5 (0.0, 2.0)	1.8 (0.0, 3.0)	1.33 (1.07, 1.63)
Body weight	kg	29.7 (22.0, 41.5)	25.2 (19.9, 33.3)	0.98 (0.95, 1.00)
Venipuncture hematocrit	%	37.0 (34.9, 40.0)	37.8 (34.8, 39.2)	1.01 (0.94, 1.10)
Atypical lymphocyte count*	x1000 cells/mm3	0.069 (0.000, 0.163)	0.067 (0.000, 0.140)	13.64 (0.01, 45466.46)
Band cell count*	x1000 cells/mm3	0.000 (0.000, 0.124)	0.000 (0.000, 0.118)	5.09 (0.00, 133304.48)
Basophil count*	x1000 cells/mm3	0.000 (0.000, 0.088)	0.000 (0.000, 0.062)	0.00 (0.00, 36729.54)
Eosinophil count*	x1000 cells/mm3	0.000 (0.000, 0.121)	0.000 (0.000, 0.059)	0.00 (0.00, 0.50)
Lymphocyte count*	x1000 cells/mm3	0.109 (0.000, 0.195)	0.107 (0.077, 0.148)	0.00 (0.00, 7774.34)
Monocyte count*	x1000 cells/mm3	0.077 (0.000, 0.153)	0.075 (0.000, 0.115)	1.32 (0.00, 1385067.22)

Continued on next page

Description	Unit	Non-DHF	DHF	Odds Ratio
Polymorphonuclear Leukocyte count*	x1000 cells/mm3	0.105 (0.064, 0.187)	0.104 (0.078, 0.149)	3.450000e+01 (0.000000e+00, 2.791904e+10)
White blood cell count*	x1000 cells/mm3	3.5 (3.4, 3.6)	3.5 (3.3, 3.6)	0.53 (0.12, 2.41)
Albumin	g/dL	3.4 (3.1, 3.6)	3.1 (2.7, 3.4)	0.22 (0.12, 0.42)
ALT	IU/L	43 (31, 70)	56 (44, 82)	1.00 (1.00, 1.01)
AST	IU/L	81 (54, 137)	141 (87, 179)	1.00 (1.00, 1.00)
Total protein	g/dL	6.4 (6.0, 6.8)	6.2 (5.3, 6.6)	0.60 (0.43, 0.85)
Platelet count	x1000 cells/mm3	84 (50, 132)	51 (26, 89)	0.99 (0.98, 1.00)
Albumin:Globulin ratio	-	0.522 (0.492, 0.558)	0.500 (0.472, 0.545)	0.00 (0.00, 0.32)
AST:Platelet ratio	(IU/L)/(x1000 cells/mm3)	1.082 (0.447, 2.812)	2.781 (1.076, 6.111)	1.05 (1.01, 1.08)
AST:ALT ratio	-	1.851 (1.408, 2.525)	2.067 (1.720, 2.777)	1.21 (0.91, 1.54)
Day of illness	day	5 (4, 5)	5 (4, 5)	0.93 (0.70, 1.24)

Note: AST: Aspartate Transaminase, ALT: Alanine Transaminase.

*Data are presented as median (min, max).

Table S12-5: Numeric variables from symptoms and biological parameters with medians (inter-quartile ranges) and odds ratios for DSS and Non-DSS categories for Study day 4 data.

Description	Unit	Non-DHF	DHF	Odds Ratio
**Daily blood pressure**				
Systolic	mmHg	93 (90,100)	90 (88,90)	0.89 (0.82,0.95)
Diastolic	mmHg	60 (54,60)	60 (54,61)	1.03 (0.96,1.11)
**Daily pulse pressure**				
Minimum	mmHg	30 (30,36)	24 (20,30)	0.68 (0.61,0.76)
**Daily fingertip hematocrit**				
Minimum	%	37 (35,40)	38 (35,40)	1.03 (0.91,1.16)
Maximum	%	40 (37,42)	44 (41,47)	1.19 (1.07,1.32)
Average	%	38 (36,41)	41 (38,43)	1.13 (1.01,1.28)
Range	%	2 (1,4)	6 (3,10)	1.51 (1.29,1.80)
**Fluid intake and output**				
Difference between fluid intake and output	ml	350 (0,900)	1085 (380,2200)	1.00 (1.00,1.00)
Daily maximum difference between fluid intake and output	ml	300 (100,550)	575 (332,885)	1.00 (1.00,1.00)

Continued on next page

Description	Unit	Non-DHF	DHF	Odds Ratio
**Daily pulse rate**				
Minimum	beats/minute	82 (78,90)	80 (72,90)	0.98 (0.94,1.03)
Maximum	beats/minute	104 (96,112)	112 (106,124)	1.06 (1.02,1.10)
Average	beats/minute	93 (88,101)	97 (92,104)	1.03 (0.99,1.08)
Range	beats/minute	20 (14,26)	32 (23,36)	1.09 (1.04,1.14)
**Daily body temperature**				
Minimum	°C	36.7 (36.3,37.0)	36.9 (36.5,37.0)	1.14 (0.52,2.34)
Maximum	°C	38.5 (37.9,39.3)	38.9 (38.5,39.6)	1.55 (0.95,2.58)
Average	°C	37.5 (37.1,38.1)	37.8 (37.7,38.3)	1.57 (0.81,3.03)
Range	°C	1.8 (1.2,2.4)	1.9 (1.7,2.6)	1.65 (0.95,2.85)
Abdominal circumference	cm	57.0 (51.0,65.0)	56.0 (54.0,59.2)	0.98 (0.93,1.02)
Liver size	cm	1.0 (0.0,2.0)	2.0 (1.8,3.0)	1.63 (1.18,2.24)
Body weight	kg	29.6 (22.0,42.0)	26.8 (24.0,35.0)	0.99 (0.95,1.02)
Venipuncture hematocrit	%	37.0 (34.0,39.5)	40.0 (36.1,41.8)	1.11 (0.99,1.25)
Atypical lymphocyte count*	x1000 cells/mm3	0.070 (0.000,0.158)	0.070 (0.000,0.106)	2.65 (0.00,756566.59)
Band cell count*	x1000 cells/mm3	0.000 (0.000,0.123)	0.000 (0.000,0.071)	3.69 (0.00,10000928.90)
Basophil count*	x1000 cells/mm3	0.000 (0.000,0.084)	0.000 (0.000,0.070)	3.915719e+05 (0.000000e+00,8.052415e+13)
Eosinophil count*	x1000 cells/mm3	0.000 (0.000,0.107)	0.000 (0.000,0.089)	0.00 (0.00,24.30)
Lymphocyte count*	x1000 cells/mm3	0.108 (0.000,0.168)	0.107 (0.076,0.117)	0.00 (0.00,396.53)
Monocyte count*	x1000 cells/mm3	0.075 (0.000,0.163)	0.069 (0.058,0.104)	1.23580e+02 (0.00000e+00,7.53084e+11)
Polymorphonuclear Leukocyte count*	x1000 cells/mm3	0.104 (0.064,0.160)	0.104 (0.085,0.133)	1.210000e+00 (0.000000e+00,2.597002e+12)
White blood cell count*	x1000 cells/mm3	3.5 (3.3,3.6)	3.4 (3.3,3.5)	0.28 (0.03,2.29)
Albumin	g/dL	3.3 (3.0,3.6)	2.9 (2.6,3.3)	0.16 (0.06,0.40)
ALT	IU/L	48 (32,75)	76 (46,122)	1.01 (1.00,1.01)
AST	IU/L	90 (58,146)	211 (96,341)	1.00 (1.00,1.00)
Total protein	g/dL	6.4 (5.9,6.8)	5.7 (4.6,6.3)	0.44 (0.27,0.69)
Platelet count	x1000 cells/mm3	86 (45,128)	38 (19,69)	0.98 (0.97,0.99)
Albumin:Globulin ratio	-	0.520 (0.491,0.556)	0.532 (0.485,0.560)	0.71 (0.00,2395.07)
AST:Platelet ratio	(IU/L)/(x1000 cells/mm3)	1.228 (0.510,3.343)	7.486 (1.847,23.778)	1.11 (1.06,1.16)
AST:ALT ratio	-	1.932 (1.400,2.599)	2.597 (1.978,3.389)	1.84 (1.23,2.69)
Day of illness	day	5 (4,6)	5 (4,6)	0.96 (0.63,1.42)

Note: AST: Aspartate Transaminase, ALT: Alanine Transaminase.

*Data are presented as median (min, max).

Table S12-6: Numeric variables from symptoms and biological parameters with medians (inter-quartile ranges) and odds ratios for DSS and Non-DSS categories for Study day 5 data.

Description	Unit	Non-DHF	DHF	Odds Ratio
**Daily blood pressure**				
Systolic	mmHg	94 (90, 100)	90 (86, 94)	0.90 (0.78, 1.03)
Diastolic	mmHg	60 (52, 60)	61 (60, 63)	1.16 (0.97, 1.44)
**Daily pulse pressure**				
Minimum	mmHg	30 (30, 37)	26 (20, 30)	0.73 (0.60, 0.86)
**Daily fingertip hematocrit**				
Minimum	%	37 (34, 38)	39 (32, 42)	1.10 (0.91, 1.40)
Maximum	%	39 (36, 42)	44 (38, 47)	1.29 (1.04, 1.66)
Average	%	38 (36, 40)	40 (36, 45)	1.21 (0.97, 1.56)
Range	%	2 (1, 4)	6 (4, 7)	1.48 (1.10, 2.06)
**Fluid intake and output**				
Difference between fluid intake and output	ml	355 (0, 800)	1520 (1460, 1648)	1.00 (1.00, 1.00)
Daily maximum difference between fluid intake and output	ml	330 (0, 500)	905 (638, 1090)	1.00 (1.00, 1.00)
**Daily pulse rate**				
Minimum	beats/minute	84 (80, 90)	83 (74, 86)	0.93 (0.84, 1.02)
Maximum	beats/minute	104 (96, 114)	111 (104, 118)	1.04 (0.97, 1.11)
Average	beats/minute	95 (88, 101)	97 (92, 100)	1.00 (0.92, 1.08)
Range	beats/minute	20 (12, 26)	38 (29, 40)	1.12 (1.04, 1.24)
**Daily body temperature**				
Minimum	°C	36.6 (36.3, 37.0)	36.5 (36.1, 37.1)	1.50 (0.31, 6.12)
Maximum	°C	38.3 (37.7, 39.2)	38.0 (37.3, 39.1)	0.84 (0.34, 1.97)
Average	°C	37.5 (37.0, 38.0)	37.2 (36.7, 38.1)	0.93 (0.25, 3.12)
Range	°C	1.6 (1.0, 2.3)	1.5 (1.1, 1.8)	0.67 (0.21, 1.80)
Abdominal circumference	cm	56.0 (50.0, 64.0)	58.0 (54.5, 60.0)	0.99 (0.90, 1.07)
Liver size	cm	1.0 (0.5, 2.0)	3.0 (1.5, 3.0)	1.99 (1.03, 4.06)
Body weight	kg	28.0 (20.5, 38.8)	31.8 (26.2, 41.0)	1.01 (0.95, 1.07)
Venipuncture hematocrit	%	36.5 (34.0, 39.3)	39.6 (36.8, 42.3)	1.13 (0.93, 1.41)
Atypical lymphocyte count*	x1000 cells/mm3	0.073 (0.000, 0.125)	0.079 (0.056, 0.113)	4.121628e+07 (0.000000e+00, 1.543118e+23)

Continued on next page

Description	Unit	Non-DHF	DHF	Odds Ratio
Band cell count*	x1000 cells/mm3	0.000 (0.000, 0.114)	0.000 (0.000, 0.107)	2.232720e+03 (0.000000e+00, 3.782991e+13)
Basophil count*	x1000 cells/mm3	0.000 (0.000, 0.084)	0.000 (0.000, 0.000)	0.00 (NA, Inf)
Eosinophil count*	x1000 cells/mm3	0.000 (0.000, 0.103)	0.000 (0.000, 0.067)	0.00 (0.00, 77360.03)
Lymphocyte count*	x1000 cells/mm3	0.113 (0.000, 0.162)	0.112 (0.089, 0.132)	0.000000e+00 (0.000000e+00, 2.611443e+20)
Monocyte count*	x1000 cells/mm3	0.081 (0.000, 0.123)	0.077 (0.000, 0.126)	0.00 (0.00, 417075.76)
Polymorphonuclear Leukocyte count*	x1000 cells/mm3	0.106 (0.071, 0.159)	0.110 (0.086, 0.144)	3.277698e+06 (0.000000e+00, 8.878039e+26)
White blood cell count*	x1000 cells/mm3	3.5 (3.4, 3.7)	3.5 (3.3, 3.7)	1.01 (0.02, 50.18)
Albumin	g/dL	3.3 (3.0, 3.6)	3.0 (2.8, 3.1)	0.32 (0.07, 1.58)
ALT	IU/L	58 (35, 101)	96 (66, 122)	1.00 (0.98, 1.01)
AST	IU/L	102 (60, 194)	173 (152, 344)	1.00 (1.00, 1.01)
Total protein	g/dL	6.4 (5.9, 6.8)	5.6 (4.9, 6.3)	0.71 (0.40, 1.51)
Platelet count	x1000 cells/mm3	81 (51, 142)	42 (28, 57)	0.98 (0.94, 1.00)
Albumin:Globulin ratio	-	0.516 (0.486, 0.554)	0.490 (0.482, 0.552)	0.91 (0.00, 1430282.93)
AST:Platelet ratio	(IU/L)/(x1000 cells/mm3)	1.732 (0.513, 3.878)	4.111 (3.422, 6.740)	1.03 (0.85, 1.12)
AST:ALT ratio	-	1.888 (1.350, 2.472)	2.325 (2.042, 3.147)	2.04 (0.85, 4.94)
Day of illness	day	5 (5, 6)	6 (5, 6)	1.10 (0.51, 2.10)

Note: AST: Aspartate Transaminase, ALT: Alanine Transaminase.

*Data are presented as median (min, max).
